# Three near-complete genome assemblies reveal substantial centromere dynamics from diploid to tetraploid in Brachypodium genus

**DOI:** 10.1186/s13059-024-03206-w

**Published:** 2024-03-04

**Authors:** Chuanye Chen, Siying Wu, Yishuang Sun, Jingwei Zhou, Yiqian Chen, Jing Zhang, James A. Birchler, Fangpu Han, Ning Yang, Handong Su

**Affiliations:** 1 National Key Laboratory of Crop Genetic Improvement, Hubei Hongshan Laboratory, Shenzhen Institute of Nutrition and Health, Huazhong Agricultural University, Wuhan, 430070 China; 2 State Key Laboratory of Plant Cell and Chromosome Engineering, Institute of Genetics and Developmental Biology, Innovation Academy for Seed Design, Chinese Academy of Sciences, Beijing, 100101 China; 3 University of the Chinese Academy of Sciences, Beijing, 100049 China; 4 Division of Biological Sciences, University of Missouri, Columbia, MO 65211 USA; 5 Shenzhen Branch, Guangdong Laboratory for Lingnan Modern Agriculture, Genome Analysis Laboratory of the Ministry of Agriculture, Agricultural Genomics Institute at Shenzhen, Chinese Academy of Agricultural Sciences, Shenzhen, China

**Keywords:** Centromere, Near-complete genome, Satellite repeat, CENH3, *Brachypodium*, Alloploidization

## Abstract

**Background:**

Centromeres are critical for maintaining genomic stability in eukaryotes, and their turnover shapes genome architectures and drives karyotype evolution. However, the co-evolution of centromeres from different species in allopolyploids over millions of years remains largely unknown.

**Results:**

Here, we generate three near-complete genome assemblies, a tetraploid *Brachypodium hybridum* and its two diploid ancestors,* Brachypodium distachyon* and *Brachypodium stacei*. We detect high degrees of sequence, structural, and epigenetic variations of centromeres at base-pair resolution between closely related *Brachypodium* genomes, indicating the appearance and accumulation of species-specific centromere repeats from a common origin during evolution. We also find that centromere homogenization is accompanied by local satellite repeats bursting and retrotransposon purging, and the frequency of retrotransposon invasions drives the degree of interspecies centromere diversification. We further investigate the dynamics of centromeres during alloploidization process, and find that dramatic genetics and epigenetics architecture variations are associated with the turnover of centromeres between homologous chromosomal pairs from diploid to tetraploid. Additionally, our pangenomes analysis reveals the ongoing variations of satellite repeats and stable evolutionary homeostasis within centromeres among individuals of each *Brachypodium* genome with different polyploidy levels.

**Conclusions:**

Our results provide unprecedented information on the genomic, epigenomic, and functional diversity of highly repetitive DNA between closely related species and their allopolyploid genomes at both coarse and fine scale.

**Supplementary Information:**

The online version contains supplementary material available at 10.1186/s13059-024-03206-w.

## Background

Centromeres are essential for the accurate segregation of replicated chromosomes during cell division [1, 2]. In many plants, centromeres consist of large arrays of species-specific satellite repeats, interspersed with long-terminal repeat (LTR) retrotransposons [3]. These repetitive DNA sequences are packaged around CENH3 nucleosomes, a centromeric-specific histone H3 variant that determines centromere identity and function epigenetically [4, 5]. Despite the conserved function of centromeres, the underling repetitive sequences can rapidly and substantially evolve across different species [6, 7]. Centromere variations can shape eukaryotic karyotypes and contribute to genomic evolution in closely related organisms, ultimately leading to reproductive isolation and speciation [8–12]. Understanding the mechanisms driving centromere evolution and their impact on genome structure and function is crucial for unraveling the processes of eukaryotic evolution and speciation.

Allopolyploidy, arising from wide hybridization and whole-genome duplication (WGD), is a significant driving force in the diversification and speciation of higher plants [13]. However, when two sets of parental chromosomes merge within a single nucleus, the presence of centromeres from different sources can result in genetic conflicts, leading to genome elimination and karyotype instability in multiple polyploid systems [14–17]. Therefore, the adaptability of centromeres during this process is important for maintaining genomic stability and preventing aneuploidy. However, genomic variations in centromeres and their contributions to the adaptation of polyploidization process remains an ongoing area of research.

Recent advancements in ultra-long DNA sequencing technologies and computational tools have enabled detailed analyses of structural variations within repetitive centromeric regions at a high resolution [18, 19]. Several model species have had their gap-free reference genomes assembled, revealing abundant genomic variations within these previously hidden regions [20–24]. These studies have provided valuable insights into the genetic and epigenetic landscape of centromeres, proposing a layered expansion model that sheds light on the mechanisms driving centromere evolution [22, 23, 25]. Satellite homogenization and retrotransposon invasions have been identified as opposing force driving centromere evolution in *Arabidopsis* and *Erianthus rufipilus* [21, 24]. Study in *Arabidopsis* population reported centromere diversity, and rapid cycles of transposon invasion and purging through satellite homogenization promote centromere evolution [26]. However, the mechanism that drives centromere differentiations between different species remain unclear.

*Brachypodium* is a genus of small annual grass that encompasses multiple ploidy levels. Cytological and comparative optical mapping studies have shown that the allotetraploid *B. hybridum* (2n = 4x = 30) is originated from a hybridization event between two diploid progenitor species, *B. distachyon* (2n = 2x = 10) and *B. stacei* (2n = 2x = 20) [27, 28]. The presence of nested insertions of whole chromosomes, specifically within centromeric regions, contributing to the formation of chromosomes from a common ancestor in the *Brachypodium* genus [29, 30]. Due to their small genome sizes and amenability to experimental investigations, these species are excellent models for studying centromere evolution in allopolyploids [31–33]. Previous studies have proposed the division of *Brachypodium* genus into two distinct lineages based on the presence or absence of centromeric-specific retrotransposons [34]. However, the detailed composition of centromeres and their evolution in the *Brachypodium* genus has remained elusive. In this study, we constructed three near-complete genome assemblies for *B. hybridum*, *B. distachyon*, and *B. stacei*, and provided the first comprehensive comparison of previously inaccessible centromeric regions across species with different polyploidy levels. Our findings offer novel scientific insights into the sequences, structural, and epigenetic variations in centromeres of closely related species, and their potential roles during polyploidy adaptions at base-pair resolution.

## Results

### Three near-complete genome assemblies in Brachypodium genus with different ploidy levels

**Fig. 1:**
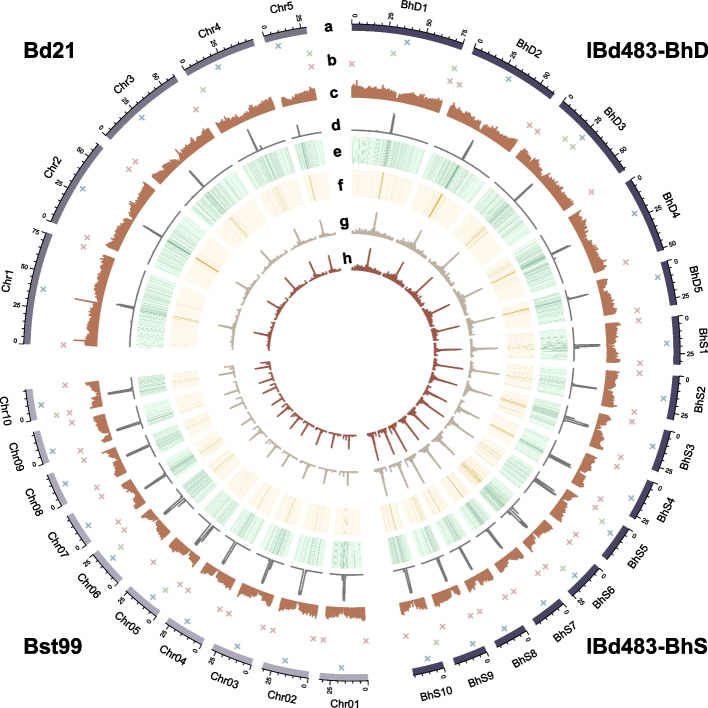
Three near-complete genome assemblies in *Brachypodium* genus. Circos plot of the IBd483-CEN, Bd21-CEN and Bst99-CEN assemblies. *a* chromosome; Quantitative tracks (labeled *b* to *h*) are aggregated in 100-kb bins, and independent *y*-axis labels are given as follows: *b* chromosome with centromeres shown in blue, telomeres (brown) and gaps (green). *c* Genes density; *d* Centromeric tandem repeats; *e* Transposable elements density; *f* Centromere retrotransposon; *g,h* CENH3 enrichment [log_2_(ChIP/Input)], IBd163, and IBd483 lines for IBd483-CEN assembly; Adi-3 and Bd21 lines for Bd21-CEN assembly; Bst92 and Bst99 lines for Bst99-CEN assembly

We generated three high-quality reference genomes for lines of *B. hybridum* (IBd483), *B. distachyon* (Bd21), and *B. stacei* (Bst99) utilizing 39.6 Gb, 19.7 Gb, and 19.4 Gb PacBio HiFi reads, and the polished contigs exhibited N50 values of 29.7, 60.3, and 22.9 Mb, respectively (see “Methods” section; Additional file 2: Table S1). The contigs from Bd21 were directly anchored into 5 pseudochromosomes, while those from IBd483 and Bst99 were anchored into 15 and 10 pseudochromosomes, with the aid of approximately 135–140 × high-throughput chromatin conformation capture (Hi-C) data (Additional file 2: Table S2). The final improved assemblies yielded 535.10, 274.75, and 258.94 Mb of sequences for IBd483, Bd21, and Bst99 lines, respectively, with only 8, 2, and 3 gaps (Fig. 1; Table 1; Additional file 2: Table S3). We designated the five chromosomes from the BhD subgenome of IBd483 as BhD1 through BhD5, and ten chromosomes from the BhS subgenome as BhS1 through BhS10; with their corresponding chromosomes as Chr1 to Chr5 in Bd21, and Chr01 to Chr10 in Bst99. The completeness of these three new genome assemblies was verified by their high mapping ratio (99.98–100%) of HiFi reads. The average chromosome size of D sub/genomes (55.78 Mb in BhD of IBd483; 54.95 Mb in Bd21) was twice that of the S sub/genomes (25.62 Mb in BhS of IBd483; 25.80 Mb in Bst99) (Fig. 1a). Additionally, plant-specific telomeric repeats (TTTAGGG)n were annotated at the ends of most chromosomes (53/60) in the three genomes (Fig. 1b; Table 1; Additional file 2: Table S4). Our Hi-C maps confirmed the high accuracy of the chromosome structure across IBd483 and Bst99 reference genomes (Additional file 1: Fig. S1). Moreover, the new Bd21 assembly exhibited high concordant with the previously reported Bd21 (v3.1) genome [35], but the IBd483 and Bst99 assemblies showed less synteny with the previously assembled reference genomes of ABR113 (v1.1) and ABR114 (v1.1), respectively [30] (Additional file 1: Fig. S2). Partial translocations and inversions that were validated by Hi-C interaction data were detected in the new IBd483 and Bst99 assemblies (Additional file 1: Fig. S2b, c). They could be caused by assembly quality or lineage specific. Overall, we observed that the majority of the two subgenomes in the IBd483 assembly were collinear with their corresponding diploid ancestral Bd21 and Bst99 (Additional file 1: Fig. S3a, b). These results underscore the gradual adaptation to allopolyploidy in the *Brachypodium* genus [30].

We annotated a total of 69,991, 34,305, and 29,801 non- transposable element (TE) gene models for IBd483, Bd21, and Bst99, respectively (Fig. 1c; Table 1), by mapping models from the previously well-annotated reference genomes [30]. Our three new assemblies achieved 98.6–99.5% completeness according to the Benchmarking Universal Single-Copy Orthologs (BUSCO) assessment (Table 1). However, it is worth noting that some genes may be missing in the *B. hybridum* and *B. stacei* genomes due to intraspecific variations. Additionally, we identified 285.69, 132.23, and 122.03 Mb of repetitive sequences in the IBd483, Bd21, and Bst99 genome assemblies, respectively, accounting for 53.39, 48.13, and 47.13% of the genomes. LTRs constituted 19.22, 20.98 and 15.53%, of the three genomes, with LTR/Gypsy elements accounting for 13.25, 15.72, and 9.03%, and LTR/Copia elements accounting for 5.16, 4.34, and 5.34%, respectively (Fig. 1e; Additional file 2: Table S5). Analysis of the insertion time of full-length LTRs in the three genomes revealed gradual TE modifications occurring at or after the WGD event (Additional file 1: Fig. S3c). Compared to previously published genome assemblies [30, 35], we added approximately 22.59, 4.43, and 16.53 Mb of novel sequences within or near centromeres in the new genomes (Additional file 1: Fig. 2). Moreover, only four gaps were detected within the centromeric regions, and 18 chromosomes were wholly resolved in the three assemblies (Fig. 1b; Additional file 2: Table S3, S4). These results demonstrate that we have achieved three near-complete genome assemblies, designed as IBd483-CEN, Bd21-CEN, and Bst99-CEN, in the *Brachypodium* genus, encompassing both tetraploid and diploid levels.

**Table 1:** Statistics for three genome assemblies in *Brachypodium* genus

Genomic feature	IBd483-CEN	Bd21-CEN	Bst99-CEN
Contig N50 (bp)	29,691,028	60,349,883	22,922,459
Scaffold N50 (bp)	32,250,776	60,349,883	26,108,307
Total length (bp)	535,099,749	274,751,009	258,935,550
Number of Gaps	8	2	3
Number of telomeres	27	7	19
Number of non-TE gene model	69,991	34,305	29,801
Complete BUSCOs (%)	99.5	98.6	99.0

**Fig. 2:**
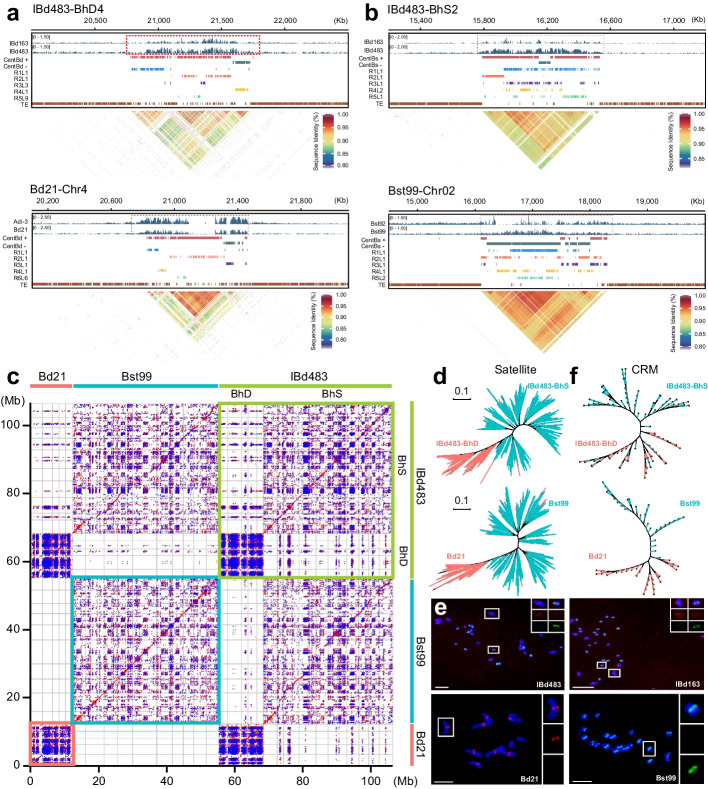
Comprehensive map of centromeres in *Brachypodium* genus. **a**,** b** Characteristics of the centromeres in *Brachypodium* genome assemblies. CENH3 enrichment [log_2_(ChIP/Input)] plotted over centromere BhD4 (ChIP samples from IBd163 and IBd483) in IBd483-CEN and CEN4 (ChIP samples from Adi-3 and Bd21) in Bd21-CEN assembled genome (**a**), and centromere BhS2 in IBd483-CEN and CEN02 (ChIP samples from Bst92 and Bst99) in Bst99-CEN assembled genome (**b**). Different layers demonstrate the CENH3 enrichment, CentBd or CentBs distribution with forward- (red) or reverse- (green) strand orientations, the structure of top five ranked frequent HORs (higher-order repeats), TE (transposon element) annotations, and heatmap of pairwise CentBd or CentBs satellite sequence similarity, respectively. **c** Dot plots comparing the centromeres from distinct genome assemblies using a search window of 300 bp. Light red, cyan, and light green lines/boxes represent the centromeric regions from Bd21-CEN, Bst99-CEN, and IBd483-CEN genomes. Red and blue points indicate forward- and reverse-strand similarity, respectively. **d** Phylogenetic tree of all CentBd (red) and CentBs (blue) repeats with branches colored by subgenomes in IBd483-CEN (top), or by Bd21-CEN and Bst99-CEN genomes (bottom). **e** Oligo-FISH mapping of satellite repeats on metaphase chromosomes of IBd483, IBd163, Bd21, and Bst99 lines. Oligo-probes (probes 1a + 2a for CentBd (red) and probes 1b + 2b for CentBs (green), as illustrated in Additional file 1: Fig. S11b) are hybridized simultaneously. The boxed insets show higher-magnification views of chromosomes. Chromosomes are colored in blue with 4’,6-diamidino-2-phenylindole (DAPI). Scale Bar = 10 μm. **f** Phylogenetic tree of all CRM retrotransposons within the core centromeres. The branches are colored by subgenomes in IBd483-CEN (red for BhD subgenome and blue for BhS subgenome, top), or by Bd21-CEN (red) and Bst99-CEN (blue) genome (bottom)

### Comprehensive maps of centromere in Brachypodium genus with different ploidy levels

To investigate the centromere landscape within the *Brachypodium* genus, we conducted ChIP-seq experiments using specific anti-CENH3 antibodies on two inbred lines from each species (Additional file 1: Fig. S4; Additional file 2: Table S6. IBd163 and IBd483 for *B. hybridum*; Adi-3 and Bd21 for *B. distachyon*; Bst92 and Bst99 for *B. stacei*). CENH3 occupancy was leveraged to accurately define the boundaries of core centromeres on each chromosome within the three genomes (Figs. 1g, h and 2a, b; Additional file 1: Fig. S5-S7; Additional file 2: Table S7). Most centromeres in the three assemblies exhibited no breakpoints of HiFi reads in CENH3-enriched regions, indicating the high quality and reliability of these centromeres (Additional file 1: Fig. S8). The sizes of centromeres in the BhS subgenome (ranging from 0.80 to 2.40 Mb) were generally larger than those in the BhD subgenome (ranging from 0.55 to 1.30 Mb) in the IBD483-CEN assembly, consistent with their two respective diploid progenitors. In Bst99-CEN, centromere sizes ranged from 2.50 Mb on Chr05 down to 1.40 Mb on Chr10, and in Bd21-CEN, they ranged from 1.25 Mb on Chr1 down to 0.74 Mb on Chr4 (Additional file 2: Table S7). These findings suggest that smaller chromosomes in the S sub/genomes (BhS subgenome of *B. hybridum* and *B. stacei*) tend to possess larger centromeres compared to the D sub/genomes (BhD subgenome of *B. hybridum* and *B. distachyon*), potentially providing stronger kinetochore strength for proper chromosome segregation [36]. Furthermore, the genomic landscape of centromeres differs substantially between the D and S sub/genomes in the *Brachypodium* genus (Fig. 2c). Each centromere exhibits a unique sequence and structural characteristics on the chromosome of three genome assemblies, and distinct CENH3 profiles were also observed between the two lines within each species (Additional file 1: Fig. S5-S7, S9). The syntenic chromosomes relationships between *B. distachyon* and the corresponding *B. stacei* and *O. sativa* revealed that *Brachypodium* chromosome evolved from an ancestral chromosome through a series of nested chromosomes insertions, chromosomes fission, or collinearity of chromosomes (Additional file 1: Fig. S10a), as previously reported [30]. These results clearly demonstrate centromere divergences between rice and *B. stacei* or between *B. stacei* and *B. distachyon*, suggesting post-speciation turnover of centromeres. Furthermore, the centromere loss was illustrated during the nested insertions of chromosomes or fusion of chromosomes between *O. sativa* and *Brachypodium* genus. Conserved centromeres were shown between Bd21 or Bst99 chromosomes with those chromosomes of D- and S- subgenomes of *B. hybridum*, respectively (Additional file 1: Fig. S10b). Other chromosome regions exhibit a predominantly collinear pattern in the syntenic relationship maps, indicating different evolutionary paths between the centromeres and chromosome-arm regions. Comparative analysis of syntenic centromere sequences among these species indicated significant difference across species (Additional file 1: Fig. S11). These results demonstrate that rapid interspecies centromere evolution and complete turnover have occurred during or after speciation.

Centromeres in *B. distachyon* are characterized by a head-to-tail arrangement of a 156-bp satellite repeat [34]. To determine the composition of centromeres in the other two *Brachypodium* species, we performed a de novo construction using ChIP-seq reads and identified two types of satellite DNAs with consensus repeat sequences of 156-bp and 157-bp in the tetraploid lines (Additional file 1: Fig. S12; Additional file 2: Table S8). These two types of satellite repeats, with 19 SNPs and 1 INDEL, were associated with CENH3 nucleosomes on each centromere (Fig. 1d; Additional file 1: Fig. S12b). Interestingly, they were identical to those detected in their corresponding diploid lines (Additional file 1: Fig. S12a-c). Phylogenetic analysis of the satellite repeats derived from the IBd483-CEN genome showed that they could be divided into two subclades based on their subgenome sources, and the satellite monomers from the Bd21-CEN were separated from those of the Bst99-CEN. Only a few individual repeats from the D or S sub/genomes were intermingled (Fig. 2d). These results indicate that two types of centromeric satellite variants arise through point mutations, deletions, or insertions across closely related *Brachypodium* species. We designated these satellites as CentBd and CentBs according to their sub/genomic sources, respectively.

Cross-alignments of ChIP/Input-seq reads to the CentBd or CentBs consensus sequence further confirmed the divergence of satellite repeats within the centromeres of D and S sub/genomes (Additional file 1: Fig. S12d, e). To confirm the distinct centromeric satellite variants, we designed oligo-probes targeting the different nucleotides between them, and the FISH signals clearly distinguished the sub/genomic centromeres (Fig. 2e), providing cytogenetic differentiation of satellite repeats within the centromeres of *Brachypodium* genus. The genomic abundance of CentBs was approximately 5.63-fold greater than that of CentBd repeats in IBd163 and IBd483 samples, and a similar abundance difference was observed between Bst99-CEN and Bd21-CEN genomes (Additional file 1: Fig. S12a, d, e), which was also reflected by the FISH signal intensity among these lines (Fig. 2e). Moreover, we observed divergent LTR elements in the centromeres between the D and S sub/genomes in the three species (Figs. 1f and 2f), as previously described [34]*.* Earlier research highlighted the presence of nested insertions of whole chromosomes into centromeres or other chromosome rearrangements that occurred in *Brachypodium* species originating from a common ancestor [29, 30]. We further compared the phylogenetically tree of satellite and LTR repeats between the syntenic centromeres across *O. sativa* and *Brachypodium* species. These results revealed that the repetitive sequences clustered together based on their species, suggesting that each species acquired private centromere satellite and CRM populations, and completely turnover of centromeres for these corresponding chromosomes post-speciation (Additional file 1: Fig. S13). In summary, our findings suggest substantial variations in the landscape of centromeres at multiple levels, including centromeric sequence contexts and abundances, as well as overall architectures between the two *Brachypodium* sub/genomes. This extensive turnover has resulted in the emergence of an exclusive repertoire of centromeric satellite and LTR populations in different species.

### Fine structure of satellite arrays in centromeres of Brachypodium genus

To achieve understanding of centromere structure within the *Brachypodium* genus at a higher resolution, we conducted a comprehensive search for tandem repeats in the three genomes to define satellite libraries. Our analysis revealed that all centromeric regions consist of extensive arrays of tandem repeat sequences spanning millions of base pairs. In the IBd483-CEN assembly, we identified approximately 16.27 Mb of satellite repeats, comprising 17,838 and 86,896 copies derived from the D and S subgenome, receptively (Additional file 2: Table S9). In the Bd21-CEN and Bst99-CEN assemblies, we identified a total of 2.18 and 15.98 Mb of satellite repeats, respectively, with copy numbers varying from 535 to 3890 per chromosome in Bd21-CEN, and from 7652 to 14,348 per chromosome in Bst99-CEN (Additional file 2: Table S10). These finding suggest that the expansion of centromeres in the S sub/genomes is largely attributed to the amplification of satellite repeats in *Brachypodium*.

**Fig. 3:**
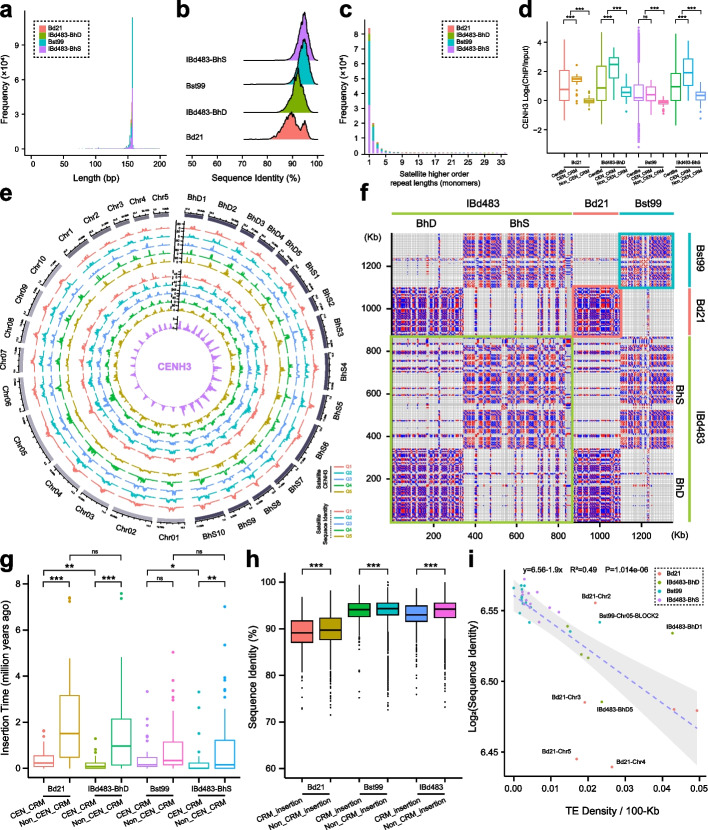
Fine structure of centromere repeat arrays in three species of *Brachypodium* genus. **a**,** b** Histograms of CentBd or CentBs monomer length (bp) (**a**) and sequences identity (**b**) relative to the genome-wide consensus in Bd21-CEN (light red), Bst99-CEN (cyan), BhD (green), and BhS (purple) subgenome of IBd483-CEN assemblies. **c** The length of HOR unit in monomer pattern from Bd21-CEN (light red), Bst99-CEN (cyan), BhD (green), and BhS (purple) subgenome of IBd483-CEN assemblies. **d** CENH3 enrichment level [log_2_(ChIP/Input)] around CentBd or CentBs satellite, centromeric intact CRM (*n* = 142 in IBd483-CEN; *n* = 39 in Bd21-CEN; *n* = 35 in Bst99-CEN) and non-centromeric intact CRM (*n* = 138 in IBd483-CEN; *n* = 68 in Bd21-CEN; *n* = 49 in Bst99-CEN) elements in Bd21, Bst99, and IBd483 lines. **e** Circos plot showing CentBd or CentBs satellite density grouped by decreasing CENH3 log_2_(ChIP/Input) (light red, cyan, blue, green, yellow), and satellite density grouped by decreasing sequence identity (light red, cyan, blue, green, yellow) in each chromosome of the three genomes. Q scale means the satellite quantile grouped by CENH3 occupancy (top) or satellite sequence identity compared with consensus repeat (bottom). CENH3 enrichments (purple) across the centromeres of the three assembled genomes were shown in the innermost circos plot. **f** Dot plot of centromeric intact CRM elements from three genome assemblies using a 75-bp search window. Light red, cyan, and light green lines/boxes represent the centromeric regions from Bd21-CEN, Bst99-CEN, and IBd483-CEN genomes. Red and blue points indicate forward- and reverse-strand similarly, respectively. **g** Comparison of the insertion time of intact CRM elements from centromere (CEN_CRM) and non-centromeres (Non_CEN_CRM) between the D and S sub/genomes in Bd21-CEN, Bst99-CEN, and IBd483-CEN genomes. **h** Comparison of the sequence identities of satellite repeats near CRM insertions (CRM_insertion) and the interior regions of centromeres (Non_CRM_insertion) in all three assembles genomes. **i** A negative relationship between LTR density/100-kb (*x* axis) and satellite monomer identity (*y*-axis) in all the chromosomes from the three genomes. Light red, cyan, light green, and purple points represent the centromeric region from Bd21-CEN, Bst99-CEN, BhD, and BhS subgenome of IBd483-CEN genomes. Several large subblocks of satellite arrays in BhS4, BhS7, Chr05, Chr07, Chr08, and Chr1 were far apart and analyzed separately (as illustrated in Additional file 1: Fig. S18b-c). *T*-test, *P < 0.05, **P < 0.01, ***P < 0.001

In most cases, centromeres in *Brachypodium* are constituted with several blocks of satellite arrays on opposite strands. However, we observed a different pattern in nine centromeres (BhD2, BhS1, BhS5, BhS10, Chr2, Chr5, Chr01, Chr04 and Chr10), where the satellite arrays were predominantly found on the same strand (Additional file 1: Fig. S5-S7). The satellite monomers in the D sub/genomes were typically around 156 bp in length, whereas the centromeres of S sub/genomes contained more 157-bp repeat monomers (Fig. 3a; Additional file 1: Fig. S14a, S15a). Additionally, we observed a minor class of CEN100 repeats on centromeres BhS1 and Chr01 (Additional file 1: Fig. S14a, S15a). We found that there were no pairwise satellite repeats shared between the D and S sub/genomes in our three assemblies (Additional file 2: Table S11), whereas there was a high degree of repetition of satellite monomers within the same chromosome in each genome, with a higher proportion of intrachromosome duplications in the D sub/genomes (53.81–66.03% in IBd483-BhD and 40.68–57.93% in Bd21-CEN) compared to the S sub/genomes (39.45–60.42% in IBd483-BhS and 37.38–52.95% in Bst99-CEN) (Additional file 2: Table S12). Almost no identical copies of monomers shared among different chromosomes within each genome (Additional file 2: Table S12). Phylogenetic trees suggested that most of the satellite repeats from each chromosome were clustered into distinct branches (Additional file 1: Fig. S16, S17). These results suggested that the local homogenization of satellite repeats in centromeric regions of each *Brachypodium* species.

We found that the satellite repeats from the D sub/genomes displayed a higher degree of polymorphisms than those from the S sub/genomes (Fig. 3b; Additional file 1: Fig. S14b, S15b), as we observed that the CentBd repeats exhibited greater diversity and longer branch lengths, while the CentBs repeats were more homogeneous (Fig. 2d). Furthermore, the CentBd or CentBs satellites were found to organize into distinct higher-order repeats (HOR) arrays within each chromosome of the three genome assemblies (Fig. 2a, b; Additional file 1: Fig. S5-S7) using the HiCAT tool [37]. Unique monomer amplifications or monomic expansions were abundant in all centromeres of the genomes, and the block sizes of the HOR are highly variable in size and displayed a negative exponential distribution (Fig. 3c; Additional file 1: Fig. S18; Additional file 2: Table S13). These observations revealed distinct properties of centromeres between the D and S sub/genomes, with the satellite repeats of D sub/genomes exhibiting less sequence identity but more intrachromosome repetitions. This suggests that centromere homogenizations were accompanied by local satellite repeats bursting, yielding a private library of centromeric satellite variants within each species and each chromosome.

To further reveal the association between centromeric satellite repeats and CENH3 nucleosomes across *Brachypodium* species, we detected CENH3 enrichments within these satellites, and observed an average of 0.47–1.18 fold log_2_(ChIP/Input) enrichment in the IBd483, Bd21, and Bst99 lines (Fig. 3d). While the enrichment levels were relatively lower in the other three lines (Additional file 1: Fig. S19a), suggesting variations in centromeres between different lines within a species. The distribution of CENH3 enrichment levels varied greatly across the fully assembled centromeres, with pronounced variability and non-uniform CENH3 density (Additional file 1: Fig. S5-S7). We did not find a negative relationship between CENH3 enrichment level and satellite sequence identity in each genome (Fig. 3e; Additional file 1: Fig. S14c, d, S15c, d). Interestingly, we observed that some large CentBd arrays in the interior regions of centromeres (such as ~ 110 kb on Chr1, ~ 170 kb on Chr4, ~ 210 kb on BhD1 and ~ 120 kb on BhD3) showed a depletion of CENH3 association (Additional file 1: Fig. S5). Moreover, an interior-to-distal reduction of CENH3 enrichment on satellite repeats was observed in most centromeres of the S sub/genomes. These satellite repeats with lower CENH3 binding did not display high sequence diversity, and some were highly homogeneous on the contrary (Additional file 1: Fig. S6, S7). Furthermore, we found long tracts of CentBs satellite arrays outside of the centromeres in most chromosomes of the S sub/genomes (Additional file 2: Table S7). Together, these results indicated that the occupancy of CENH3 nucleosomes on centromeric satellites is not always preferential for those closer to the genome-wide consensus, and the complexity between the genetic and epigenetic contributions to CENH3 associations across *Brachypodium* genus. The differences in CENH3-association properties between the D and S sub/genomes may be related to their distinct centromere architectures.

### Frequency of retrotransposon invasions drove the degree of interspecies centromere diversifications in Brachypodium genus

We observed a higher continuity in the tandemly repeated arrays in the S sub/genomes compared to the D sub/genomes, and the satellite arrays were primarily disrupted by CRM retrotransposons (Fig. 2a, b; Additional file 1: Fig. S5-S7). We identified a total of 216 full-length CRM elements within the core centromeres (39 in Bd21, 60 in IBD483-BhD; 35 in Bst99, and 82 in IBD483-BhS), as well as 255 full-length CRM elements located outside the centromeres (68 in Bd21, 85 in IBD483-BhD; 49 in Bst99, and 53 in IBD483-BhS) in the three genomes (Additional file 2: Table S14). Additionally, we detected fragmented and nested LTR insertion events within the centromeres of the three genomes (Fig. 2; Additional file 1: Fig. S5-S7). The dot plot of centromeric CRM elements indicated that they differ widely in sequences between D and S sub/genomes (Fig. 3f), and the CRM elements within centromeres were classified into six clades and clustered with those located outside the centromeres (Additional file 1: Fig. S20), suggesting that these CRM elements were duplicated post-integration into the centromeres. We observed an overall higher enrichment level of CENH3 on the centromeric CRM retrotransposons relative to the satellite arrays, and the enrichment levels were higher than those CRMs located outside the centromeres in each genome (Fig. 3d; Additional file 1: Fig. S19a). All centromeres contained very young CRM retrotransposons, with an average estimated insertion times of less than 0.5 million years ago (Mya), while older CRMs were distributed outside the core centromeres in the three genomes (Fig. 3g). These observations support a model in which layered expansion of centromeric arrays and retrotransposon amplifications may promote this process [23, 25, 26].

We found that the sequence identities of satellite monomer near CRM insertions were significantly lower than those located in the interior arrays in each genome (Fig. 3h). The insertion frequency of LTR elements varies greatly between the D and S sub/genomes, with the S sub/genomes having very low LTR distributions, but the D sub/genomes had higher LTR density (Additional file 1: Fig. S5-S7). We observed a negative relationship between LTR density and satellite identity in the chromosomes of the three genomes, where D sub/genomes has a more pronounced satellite sequence divergence than those from S sub/genomes (Fig. 3i). The S sub/genomes had fewer LTR insertions and less satellites divergence, while the D sub/genomes had higher LTR density and a lower satellite identity distribution (Fig. 3i; Additional file 1: Fig. S19b, c). Several large sub-blocks of satellite arrays (BhS4, BhS7, Chr05, Chr07, Chr08, and Chr1) were far apart and interrupted by abundant LTR invasions (Additional file 1: Fig. S6, S7), and the monomers in each sub-block of CentBs arrays were consistent with the aforementioned results (Fig. 3i; Additional file 1: Fig. S19b, c; Additional file 2: Table S15). Together, these comparisons of completely assembled centromeres demonstrate that abundant LTR insertions exhibit a significantly higher degree of satellite DNA polymorphisms across the *Brachypodium* genus, and LTR invasion and purging may drive the interspecies diversity of centromere sequence variations and distinct centromere architectures [26].

### Genetic and epigenetic variation across centromeres from diploid to tetraploid in Brachypodium genus

**Fig. 4:**
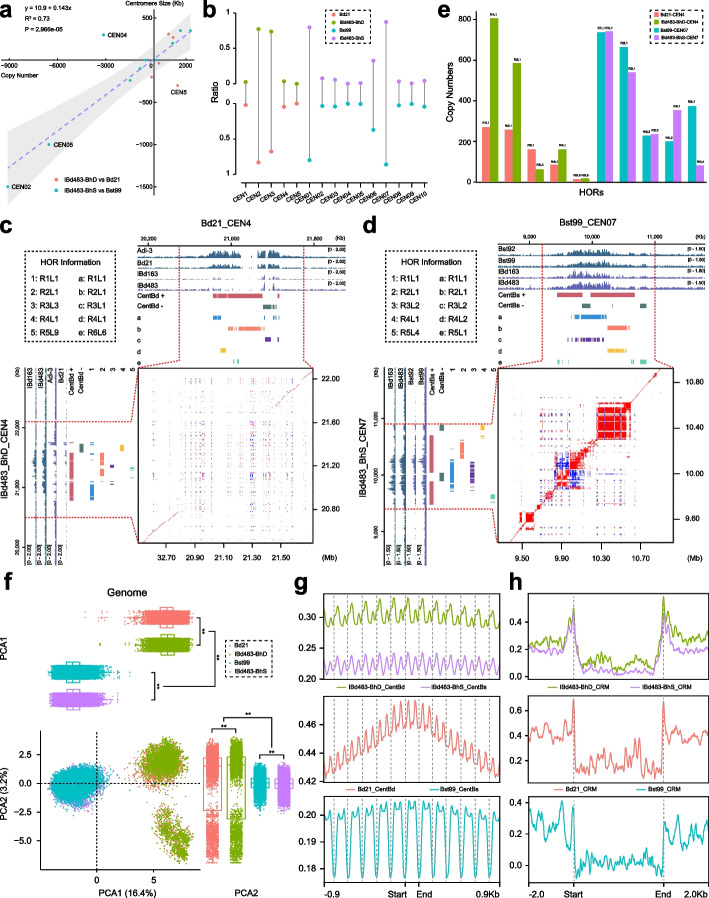
Genetic and epigenetic variation across centromeres from diploid to tetraploid in *Brachypodium*. **a** Comparison of the changes in centromere size and centromeric satellite copy number variations between homologous chromosome pairs from diploid to tetraploid in *Brachypodium*. Colored points represent the comparison between BhD-IBd483 and Bd21 (light red), as well as BhS-IBd483 and Bst99 (cyan). **b** Comparison of the proportion of satellite monomer duplications in the centromeres from homologous chromosome pairs between diploid to tetraploid in *Brachypodium*. Light red, cyan, light green, and purple points represent the sharing proportion of satellite repeats from Bd21-CEN, Bst99-CEN, BhD, and BhS subgenome of IBd483-CEN genomes. **c**,** d** CENH3 ChIP-seq mapping coverage from diploid lines Adi-3, Bd21 (**c**) or Bst92, Bst99 (**d**), and tetraploid IBd163, IBd483 lines to Bd21-CEN (**c**) or Bst99-CEN (**d**) genome with coordinates at top. The mapping coverage from IBd163, IBd483 and Adi-3, Bd21 (**c**) or Bst92, Bst99 lines (**d**) to IBd483-CEN genome with coordinates at left side. Forward- and reverse-strand satellite monomer sequences and top five ranked HOR arrays are annotated on CEN4 (**c**) and CEN07 (**d**) (Track 5–11). Dot plots compare the homologous centromeres between diploid to tetraploid *Brachypodium* using a search window of 156 bp. Red and blue points indicate forward- and reverse-strand similarity, respectively. **e** The structure and copy number of top five frequent HORs in the centromeres (CEN4 or CEN7) from homologous chromosomes between diploid to tetraploid *Brachypodium*. Light red, cyan, light green, and purple histograms represent the HOR structure from corresponding centromeres of Bd21-CEN, Bst99-CEN, BhD, and BhS subgenome of IBd483-CEN genomes. **f** PCA projection of 5-mer frequency vectors for centromeric satellite repeats (CentBd or CentBs) on PC1(first principal component) and PC2 (second principal component) from different assembled *Brachypodium* genomes. Each point represents an individual copy of satellite and colored according to the sub/genomes. Light red, cyan, light green, and purple points represent satellites from Bd21-CEN, Bst99-CEN, BhD, and BhS subgenome of IBd483-CEN genomes. Box plots on the top or right of PCA projection show the overall distribution of PC1 and PC2 scores between different sub/genome groups (*T*-test, ** *P* < 0.01). **g**, **h** Metaprofiles of CENH ChIP signals [log_2_(ChIP/Input)] around CentBd/CentBs satellite repeats (**g**) and centromeric intact CRM elements (**h**). The peaks reflect the distribution of CENH3 nucleosomes across the centromeric repetitive sequences. Different colored lines represent the CENH3 nucleosome distributions on CentBd/CRM repeat of Bd21 (light red, Bd21_CentBd/CRM), IBd483-BhD subgenome (light green, IBd483-BhD_CentBd/CRM), or on CentBs/CRM repeat of Bst99 (cyan, Bst99_CentBs/CRM), IBd483-BhS subgenome (purple, IBd483-BhS_CentBd/CRM)

To gain a deeper insight into the centromere dynamics during allopolyploidization in *Brachypodium* genus, we mapped ChIP-seq reads from tetraploid lines to diploid reference genomes and vice versa. This investigation revealed that there were no apparent changes in the overall location of centromeric regions between them (Additional file 1: Fig. S21, S22). Comparative analysis revealed that the majority of genes located in the pericentromeric regions of IBd483-CEN were collinear with their corresponding diploid ancestors (Additional file 1: Fig. S23). However, some centromeric genes were not found on the homologous centromeres between diploid and tetraploid genomes, revealing large-effect structural rearrangements, including inversions within these dark regions (Additional file 1: Fig. S23; Additional file 2: Table S16). We also examined the changes in centromere size and satellite copy number between the tetraploid and its diploid progenitors. Specifically, CEN02 and CEN05 in the S sub/genomes underwent significant size reductions from diploid to tetraploid levels due to a substantial decrease in satellite repeats (Fig. 4a; Additional file 1: Table S7). Interestingly, CEN5 in Bd21, despite having minimal amounts of CentBd repeats, displayed a larger centromere size compared to its corresponding centromere BhD5 in IBd483. This was attributed to the association of a large number of transposable elements (TEs) with CENH3 nucleosomes on the left side of CEN5 in Bd21 (Fig. 4a; Additional file 1: Fig. S5). The expansion of centromere BhS4 in IBd483 was due to the insertion of long stretches of TEs, disrupting the satellite arrays (Fig. 4a; Additional file 1: Fig. S6), as the insertion time of CRMs from the tetraploid genomes was much younger than that of the diploid genome (Fig. 3g). These results suggest that ongoing amplification of CRM elements within the centromeres in tetraploid IBd483. Overall, the changes in centromere size varied among different chromosomes after allopolyploidization, primarily due to the changes in satellite lengths.

The distributions of CentBd satellite sequence identity became more concentrated and less divergence from Bd21 to IBd483-BhD subgenome, with no significant differences detected for the sequence polymorphisms of CentBs copies between the S sub/genomes (Fig. 3b). The phylogenetic tree showed that centromeric satellite repeats from the IBd483-CEN intermingled within the same clade with those from corresponding diploid genomes (Additional file 1: Fig. S24). We further compared the satellite monomers between tetraploid and diploid genomes and found that very few identical monomers were shared between most of the homologous chromosome pairs (Fig. 4b). For example, CEN4 in Bd21 exhibited even greater dissimilarity compared to its counterpart centromere in IBd483, with each array displaying distinct characteristics (Fig. 4c). The CENH3 profiles in these centromere pairs exhibited dramatic changes, with the majority of peaks on the satellite repeats detected in diploids missing in tetraploids or vice versa (Fig. 4c; Additional file 1: Fig. S21, S22). Distinct HOR arrays tended to differ in sequence and structure between these diverse homologous centromeres (Fig. 4e). However, four pairs of chromosomes (Chr2 – BhD2, Chr3 – BhD3, Chr01 – BhS1, Chr07 – BhS7) showed a high degree of satellite repetition (67.58 ~ 87.52%), and approximately 30% duplicate monomers were detected between CEN06 in Bst99 and IBd483 (Fig. 4b; Additional file 2: Table S12). These chromosomes exhibited high consistency in terms of length, orientation, and overall centromere structure due to a high proportion of identical monomers (Additional file 1: Fig. S21, S22). For example, CEN07 in Bst99 showed closer similarity to BhS_CEN07 in IBd483, with four separated arrays encompassing the centromere by sharing the same orientation (Fig. 4d). Most of the HOR structures in size and orientation remained preserved between these chromosomes pairs from diploid and tetraploid (Fig. 4e; Additional file 1: Fig. S21, S22). We employed a principal component analysis (PCA) approach to quantitatively measure the dynamics of centromeric satellite repeats after alloploidization [38]. Using 5-mer nucleotide compositions from satellite repeats of the three assembled genomes, we observed that they could be classified into distinctive clusters base on their sub/genomic sources. The CentBd group displayed two small subgroups due to distinct principal component 2, which is consistent with the greater diversity of CentBd satellite repeats in the samples (Fig. 4f). Similar findings were obtained from high-throughput sequencing reads covering the satellite repeats from CENH3 ChIP/Input-seq data of different lines (Additional file 1: Fig. S25). The overall distributions of PC1 and PC2 scores exhibited continuous differences within each subgroup from diploid to tetraploid (Fig. 4f; Additional file 1: Fig. S25). Collectively, these observations suggested that the homologous centromere pairs exhibited extensive variations at the sequence, architecture, and organization level, and the structural rearrangements may drive rapid turnover of centromere variants during the *Brachypodium* alloploidazation process. Our findings also indicated that there was no intersubgenomic crosstalk of centromeric satellites after polyploidization in *B. hybridum*, which may be important for maintaining the homeostasis of polyploid.

Furthermore, we found that CENH3 nucleosomes exhibit a highly phased pattern of enrichments with centromeric satellites in the three genomes, predominantly occupying similar positioning sites on CentBd and CentBs monomers between diploid genomes (Fig. 4g; Additional file 1: Fig. 26a). Minor CENH3 peaks were detected with differences at the edges and interior of satellite repeat-containing nucleotide variations between CentBd and CentBs. The main peaks on CentBd and CentBs satellite repeats remained stable in the two subgenomes of IBd483, and only the minor peaks showed difference in their associations with CENH3 nucleosomes, compared to their diploid genomes (Fig. 4g; Additional file 1: Fig. 26a). Furthermore, CENH3 was associated with the entire length of the CRM elements, exhibiting different distribution patterns between the two diploid species (Fig. 4h; Additional file 1: Fig. 26b). CENH3 positioning sites over CRMs were apparent from diploid to tetraploid (Fig. 4h; Additional file 1: Fig. 26b). Slight variations in CENH3 nucleosomes positioning on centromeric repeats may have an impact on epigenetic modifications and the evolution of centromeric repetitive sequences. We concluded that CENH3 nucleosome remained relatively stable in their positions over centromeric satellite and CRM repeats after alloploidization in *Brachypodium* genomes, despite striking variations of centromere DNA sequences.

### Pan-centromere analysis revealed ongoing variations of satellite repeats in Brachypodium genomes with different polyploidy level

**Fig. 5:**
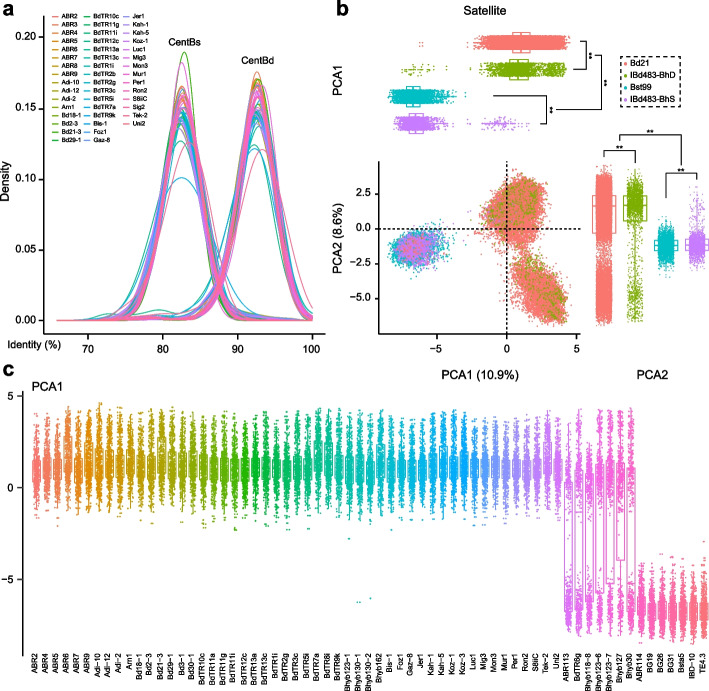
Pan-centromere analysis of the dynamic centromeric satellite repeats in *Brachypodium* genomes. **a** Sequence identity of merged fragments from WGS-seq reads to the CentBd or CentBs consensus sequence, as sampled in the pangenomes of *B. distachyon*. **b** PCA projection on PC1 and PC2 scores of sequencing fragments that cover satellite repeats from the pangenomes of different lines in the three *Brachypodium* species. The satellite fragments identified from the sequencing reads of different lines within the three *Brachypodium* genomes were merged, and 500 fragments were randomly selected for each genome. Box plots at the top and right of PCA projection show the overall distribution of PC1 and PC2 scores within each sub/genome group (*T*-test, ** *P* < 0.01). **c** Box plots show the overall distribution of PC1 scores among the individual line within the three *Brachypodium* species. ABR2 to Uni2 represents the lines from *B. distachyon*; ABR113 to Bhyb30 represents the lines from *B. hybridum*; ABR114 to TE4.3 represents the lines from *B. stacei*

To delve deeper into the origin and evolution of centromere repeats within *Brachypodium* genus, we extended our analysis to include publicly available pangenomic data of *B. distachyon*, *B. stacei*, and *B. hybridum* [30, 35]. All the centromeric satellite repeats were identified, and their sequence identities to the CentBd or CentBs consensus sequence were determined. The distributions of sequence identities revealed that all the centromeric repeats from different lines of *B. distachyon* displayed higher identity to CentBd compared to the CentBs consensus sequence (Fig. 5a). In contrast, the profiles of sequence identities from different lines of *B. hybridum* displayed two major identity peaks (Additional file 1: Fig. S27a). More satellite repeats were located in the higher peak when mapped to the CentBs consensus sequence, and more satellite repeats were found in the lower peaks when mapped to the CentBd consensus sequence (Additional file 2: Fig. S27a). This observation aligns with our previous findings that the CentBs occupied more portions in the tetraploid genome. These results suggest that divergent centromeric repeats are prevalent in the pangenome of *Brachypodium* lines, and intraspecific variations do not fully explain the diversity of centromeric satellite repeats.

To overcome the limitations of incomplete genomic assembly, we utilized publicly available raw high-throughput sequencing data from the pangenomes of the three *Brachypodium* species [30, 35], and identified all reads encompassing the centromeric CentBd/CentBs satellite repeats (Additional file 2: Table S17). The PCA projection illustrated that divergence in centromeric satellite repeats was a common occurrence among all lines within the D and S sub/genomes (Fig. 5b). The overall distributions of PCA1 and PCA2 scores also displayed significant differences between the CentBd and CentBs subgroups and pronounced variations occurred among the satellite repeats within each subgenome after alloploidization (Fig. 5b, c; Additional file 1: Fig. S27b), suggesting that ongoing variations of satellite repeats among individuals within each *Brachypodium* species. The diversity of centromeric satellite repeats was indeed influenced by intraspecific differences and geographic/climactic spreading, but maintaining a homeostasis of centromere function in each species. In summary, the ongoing and stable evolutionary pattern of centromeric satellite repeats, as revealed by *Brachypodium* pangenome studies, provide valuable new clues for a better understanding of centromere evolution among populations within a species or closely related species and after alloploidization.

## Discussion

**Fig. 6:**
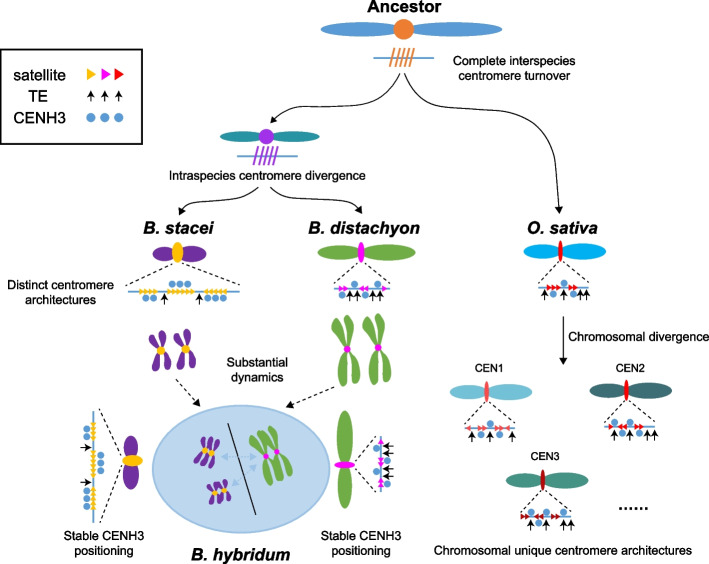
Evolution model of the dynamic centromeres in *Brachypodium* genus. Rooted in a common ancestral foundation, the absence of centromere sequences and structural conservations between these shared chromosomes of *O. sativa* and *Brachypodium* genus suggest rapid post-speciation differentiation and a complete turnover of centromeres. The centromere characteristics between *B. distachyon* and *B. stacei* exhibited notable distinctions at both localized and broad scales. *B. distachyon* exhibited lower copy numbers but greater diversity of satellite arrays, accompanied by a higher incidence of TE retrotransposon invasions within the centromeres. Conversely, *B. stacei* displayed higher copy numbers but lower polymorphisms of satellite arrays, with fewer TE invasions in the centromeres. In the tetraploid *B. hybridum*, centromeric repeats from its two diploid ancestors have merged but continue to exist independently without inter-subgenome transfer. The presence of chromosome-specific clusters of centromeric satellites suggests local chromosomal recombination-based centromere homogenization. Presumably, no intersubgenomic crosstalk appears to have occurred within the centromeric satellite repeats following allopolyploidization. The solid lines within the oval indicate the absence of crosstalk between the subgenomic centromeres in the tetraploid *Brachypodium*. Moreover, the positioning of CENH3 nucleosomes remains relatively stable, and epigenetic homeostasis of centromere is maintained within tetraploid *Brachypodium* species

Centromeres are comprised of highly repeated satellite arrays and specific retrotransposons that exhibit diverse genomic polymorphisms. The centromeric histone variant CENH3 is predominantly associated with these repetitive sequences and co-evolves as a functional unit [39, 40]. Nevertheless, when different genomes are merged and duplicated within a single nucleus, the mechanisms by which CENH3 histones from different sources are deposited and drive the evolution of centromeres after polyploidization remain largely unknown. In this study, we generated three nearly-complete genome assemblies for tetraploid* B. hybridum* and its two diploid ancestors, *B. distachyon* and *B. stacei*, using HiFi ultra-long reads (Fig. 1). Genomic analysis indicates that *B. hybridum* has two independent origins through different reciprocal crosses, occurring approximately 1.4 and 0.14 million years ago, respectively [30]. In addition, recent genome assembly studies have revealed gradual evolutionary characterizes that distinguish distinct lineages in allotetraploid *Brachypodium* [41]. Our study uncovered two subgenomic divergent centromeric satellite repeats with 19 SNPs and 1 INDEL in *B. hybridum*, which originated from its two diploid progenitors, rather than emerged after the alloploidization event (Fig. 2; Additional file 1: Fig. S11). The presence of different types of satellite repeats in diploid *Brachypodium* species suggests that they may have originated and been differential from a common ancestor over long evolutionary periods (Fig. 6). Previous results also revealed that the evolutionary young centromeric retrotransposons in *B. distachyon* (CRBds) were only detected within distinct lineages of *Brachypodium* species [34]. Based on the near-complete genome assemblies, we confirmed the divergence of CRM retrotransposons between the D and S sub/genomes (Figs. 2f and 3f; Additional file 1: Fig. S20). The deviated nucleotides within the centromeric satellite repeats and CRM sequences influence their positioning of CENH3 nucleosomes, suggesting a sequence-dependent variation of CENH3 positioning on centromeric repeats (Fig. 4g, h; Additional file 1: Fig. S26). Our findings may shed light on the initial steps of centromere repeat evolution and subsequent divergences among closely related species, providing a detailed understanding of the content of satellite repeats and retrotransposons in centromere divergence. No shared repeat satellite and transposon populations or structures were detected between syntenic centromeres of *O. sativa* and *Brachypodium* species, indicating rapid post-speciation differentiation and complete interspecies centromere turnover (Fig. 6; Additional file 1: Fig. S11 and S13). They both exhibit chromosome-specific clustered of centromere satellite populations, implying a local chromosomal recombination-based centromere homogenization in these species (Fig. 6). We also observed different evolutionary paths between the centromeres and other chromosome regions. These results are consistent with existing understanding that centromeres represent the most intricate genome structure and have evolved dramatically, generating complex genetic diversity over the course of evolution [6].

Our study also illuminates the comprehensive variation and evolution of centromeres within two closely related *Brachypodium* species and their allopolyploid at a base-pair resolution. We observed distinct centromere characteristics between the D and S sub/genomes at both localized and broad scales, encompassing sequence content, structural architecture, and epigenetic states (Fig. 2; Additional file 1: Fig. S5-S7). Centromeres from the D sub/genomes exhibited lower copy numbers but greater diversity in satellite arrays, while centromeres from the S sub/genomes displayed higher copy numbers but fewer polymorphisms in satellite arrays (Fig. 6). Additionally, we identified a higher proportion of intrachromosome duplication of satellite monomers in centromeres of the D sub/genomes compared to those of the S sub/genomes (Additional file 2: Table S11, S12). Extensive variations in centromeric satellite repeated arrays, including sequence polymorphisms, size, and structure, have been documented in various organisms [8, 38, 42–46]. Similar observations have been made in maize and its wild relatives, indicating high levels of sequence identity in individual CentC copy [47]. These findings suggest the presence of specific mechanisms that both unify and diversify satellite repeats across centromeres in different species. Studies in *Arabidopsis* have provided insights into the distinct methylation patterns within centromeric satellite repeats [21]. This has shed light on the complex associations between CENH3 nucleosome, DNA methylation, and the diverse satellite variants [21]. The intriguing correlation has raised the possibility that CENH3-nucleosomes may influence epigenetic modifications and variation in satellite sequences. However, the precise causal relationship between variations in nucleosome positioning, the divergence of satellite sequences, and potential differences in DNA methylation remains elusive. Furthermore, young TE retrotransposons have been found to disrupt the genetic and epigenetic organization of centromeres within a species by breaking centromere satellite arrays [21, 24]. In our study, we observed variations in the density of CRM insertions within centromeres between the D and S sub/genomes. We detected a negative relationship between LTR density and polymorphisms of centromere satellite arrays between *Brachypodium* species (Fig. 3h, i). These results suggest that LTRs not only play a role in driving the diversity of centromere structure on different chromosomes of the same species, but also contribute to the interspecies differentiation of centromere sequences and structures.

To expand our understanding within a broader evolutionary context, we embarked on the scrutiny of satellite repeat sequences across various species (including *O. sativa*, *B. stacei*, *B. distachyon*; Additional file 1: Fig. S13a). The detailed syntenic relationship maps of *O. sativa*, *B. stacei*, *B. distachyon*, and *B. hybridium* highlight the lost, divergent, and conserved centromeres. Our analysis has reinforced the concept that satellite sequences within centromeres exhibit rapid turnover, rooted in a common ancestral foundation during the evolution, and populate all of the centromeres within a species (Fig. 6). The properties of centromeres are distinct between the D and S sub/genomes, suggesting each species and each chromosome possesses its private library of centromeric satellite variants. Most of the homologous centromere pairs between the diploid and tetraploid exhibit extensive variations in terms of sequence, architecture, and organization (Fig. 4c, d; Additional file 1: Fig S21, S22). These structural rearrangements are associated with the rapid turnover of centromere variants during the alloploidization process. Only chromosome pairs with high consistency in length, orientation, and overall structure for centromeres exhibit a high proportion of identical satellite monomers (Fig. 4; Additional file 1: Fig. S21, S22). Ongoing variations in centromeric DNA sequences within each subgenome maintain the relatively continuous positioning of CENH3 nucleosomes over these repeats in tetraploid *B. hybridum*, compared to their corresponding diploid progenitors (Fig. 4g, h). These results indicate the presence of stable structural and organizational features of centromeric repeats that wrap around CENH3 nucleosomes following allotetraploidization. These ongoing variations, while maintaining relatively stable subgenomic homeostasis, may play a vital role in ensuring the stability of tetraploid karyotypes. However, it remains unclear whether the transfer of satellite repeats occurs between non-homologous chromosomes of subgenomes in polyploid species. In our previous work, we found evidence of intersubgenomic dispersion of centromeric satellite repeats between different subgenomes in hexaploid wheat, where centromeric satellite repeats abundant in the B-subgenome were also present in the other two subgenomes [45, 48]. In contrast, we observed that the diverged centromeric satellites from diploid progenitors remain separate between subgenomes in *B. hybridum*. Although some individual satellite repeats from the D sub/genomes were classified into the S sub/genomes in *B. hybridum*, we confirmed that they already exist in the diploid species (Fig. 2d; Additional file 1: Fig. S24). This observation may be attributed to random mutations that resulted in several individual satellite copies in *B. distachyon* being similar to those in *B. stacei*. These results demonstrate common properties of centromere evolution in tetraploid *Brachypodium*. The epigenetic plasticity and homeostasis of centromere features within each subgenome may play a crucial role in conferring adaptation and genome stability to polyploid species [49].

Although the centromere is typically considered a region of suppressed recombination during meiosis, evidence suggests that meiotic double-strand breaks and crossovers can occur within centromeric repeats. This potentially occurrence may drive the evolution of these repetitive sequences and facilitate crosstalk between centromeric satellite DNA on different chromosomes through mechanisms like unequal crossover or transposon-mediated exchange [18, 50–54]. Centromeres in many plant species are known to form associations prior to meiotic chromosome pairing, creating a close spatial proximity that may promote inter-homoeologous crossovers over evolutionary timescales [55–57]. To investigate the potential mechanisms of non-intersubgenomic exchange of centromeric satellites during meiosis in *B. hybridum*, we examined the behavior of centromere pairings using subgenomic-specific oligo-probes. Our results showed that centromeres from homologous chromosomes paired well, but no centromere associations were detected between non-homologous subgenomes during the early stage of meiosis I (Additional file 1: Fig. S28). The lack of intersubgenomic exchange of centromeric satellites in *B. hybridum* may be explained by the absence of spatial proximity between intersubgenomic centromeres during meiosis. In contrast, polyploid wheat exhibits a transformation of centromere pairing from non-homologous to homologous during the early stage of meiosis [58]. Common wheat, which originated from a hybridization event between emmer wheat (BBAA) and *Aegilops tauschii* (DD) at approximately < 0.4 Mya, falls between the dates of the ancient and recent *B. hybridum* clades [30, 59]. Thus, the absence or presence of intersubgenomic exchange of centromere repeats in polyploid species is not solely determined by the age of the polyploid species. These observations suggest that spatial proximity of intersubgenomic centromeres during meiosis may facilitate centromere rearrangements in polyploidy, as reported in *Candida*, where homology and proximity guided centromere-proximal translocations that drive karyotype evolution and centromere type transitions [60, 61]. These findings provide new insights into the mechanisms underlying the stability and evolution of centromeric satellite repeats in polyploid species.

Genomic diversity is a valuable resource for understanding molecular-level biodiversity. The concept of the pangenome, which consists a “core genome containing genes present in all strains and a dispensable genome composed of genes absent” [62], provides novel insights into the full range of genetic diversity. Pangenomes have been generated in various species, including bacteria, humans, and plants [63–65]. In plants, repetitive DNA sequences can account for up to 90% of the genome size, such as wheat, making them a rich source of functional variations [66, 67]. However, current pangenome definitions primarily focus on gene content, while information within repetitive and non-coding sequences is largely overlooked. In our study, we utilized a collection of DNA sequencing reads from the pangenome of *Brachypodium* with different polyploidy levels to capture the entire genomic diversity of centromeric satellite repeats. Subsequently, we introduced pan-centromeres to include the highly repetitive sequences that serve as important components of chromosome structure (Fig. 5). Ongoing variations of satellite repeats among the individuals within each *Brachypodium* species indicate geographic/climactic factors may indeed influence the diversity of centromeres. These results contribute to our understanding of centromere biology in the context of polyploidization and speciation at the population level, and can be applied to other systems to gain new perspectives on the integration of centromeric satellite DNA into comparative studies. Furthermore, long-read sequencing, which has emerged as a powerful tool in plant genomic studies, with an average read length of approximately 20 kb with > 99.9% accuracy, offers the capability to explore complex and diverse genomic regions, especially previously inaccessible centromere regions [68]. Recent studies have pushed the limits of HiFi assemblies and revealed natural centromere diversity between two *A. thaliana* genomes [69]. In the future, conducting multiple comparisons of centromeres across different species using gap-free genomes will provide insights into the origin and evolution of centromeres. The detailed maps of highly repetitive centromere repeats will also provide precise genome editing sites for generating distinct karyotypes and synthetic genomes [5, 70].

## Conclusion

We present three near-complete genome assemblies within *Brachypodium* genus with different polyploidy level. By comprehensively analyzing the centromeres in closely related species at both large and small length scales, we reveal striking variations of centromeric repetitive sequences driven by different level of transposable element (TE) invasions, along with their influence on the structural and epigenetics associations with CENH3 nucleosomes. Our analysis also demonstrates dramatic genetic and epigenetic architecture variations were associated with the turnover of centromeres between homologous chromosomal pairs from diploid to tetraploid. Relatively stable positioning of CENH3 nucleosomes and epigenetic homeostasis of centromere were maintained within each subgenome of *B. hybridum*, which may important for conferring adaptation and genomes stability to polyploid species. Our pan-centromere analysis also provide genomic evidence for ongoing variations, while preserving inherent stability in centromeric DNA sequences within individuals of each *Brachypodium* species. Our findings shed light on the initial steps of centromere evolution and subsequent divergences among closely related species and provide unprecedented information on the (epi)genomic and functional diversity of highly repetitive DNAs.

## Methods

### Plant materials

The *Brachypodium* lines utilized in this study were generously provided by Prof. Zhiyong Liu (Institute of Genetics and Developmental Biology of the Chinese Academy of Sciences). The IBd483 and IBd163 lines are S-type (S-plastotype accession) *B. hybridum* forming approximately 1.4 Mya. All the *Brachypodium* seeds were germinated at room temperature for 2 to 3 days until the root tips grew to 2–3 cm long. The plants were then transferred to the tissue culture room under optimal conditions (16 h day/8 h night: 24 °C/18 °C; 200 µmol/m^2^/s light). Approximately 10–20 g of leaf tissue was collected from 4-week-old seedlings for chromatin immunoprecipitation. Meiotic chromosome squashes were prepared from pollen mother cells of anther tissue at 7–8 weeks after planting. All the lines employed for cytogenetic analysis and sequencing in this study are listed in Additional file 2: Table S6, and the lines used for pangenome analysis in *B. distachyon* and *B. hybridum* are listed in Additional file 2: Table S17.

### PacBio sequencing in Brachypodium

Fresh young leaf tissue was collected from IBd483, Bd21, and Bst99 plants. We constructed SMRTbell libraries using a method described in a previous study [71]. Genomic DNA was isolated and sheared by Megaruptor (to approximately 15–20 kb), followed by damage repair, end repair, ligation with known adapters, enzyme digestion, size selection, and generation of SMRTbell structure library. The qualified libraries were sequenced on the PacBio Sequel II platform (Pacific Biosciences) at BGI-ShenZhen Company, generating approximately 39.6, 19.7, and 19.4 Gb of PacBio HiFi reads for the IBd483, Bd21, and Bst99 genome, respectively.

### Hi-C tissue fixation and library preparation

For Hi-C library construction and sequencing, we collected 3–5 g young fresh leaves tissue from IBd483 and Bst99 lines and sent to BGI-ShenZhen Company for Hi-C library construction and sequencing. Two Hi-C libraries were prepared from cross-linked chromatins using a standard Hi-C protocol as described previously [24] and sequenced on the Illumina HiSeq platform. In total, approximately 72.16 and 36.25 Gb of Hi-C reads were generated with approximately 135–140 × coverage.

### Genome assembly in Brachypodium

For Bd21, we employed HiFi sequencing data and conducted the assembly using hifiasm (v0.16.1-r375) pipeline [72]. We aligned the resulting contigs to the Bd21 (v3.1) genome [35], adjusted their order and positions, and merged them to generate scaffolds representing chromosome lengths. Regarding IBd483 and Bst99, we utilized HiFi sequencing data along with paired-end Hi-C sequencing data for the assembly process, with parameters of “hifiasm –h1 –h2.” Subsequently, we generated a non-redundant Hi-C contact matrix using juicer (v1.6) [73] and utilized it as input for 3d-dna (v180922) [74]. This yielded.hic and.assembly files suitable for visualization in juicebox. Additionally, we fine-tuned the scaffolds to achieve chromosome-length super-scaffolds for re-3d-dna pipeline, ultimately resulting in a high-quality genome assembly.

### Annotation of the Brachypodium genome

The non-transposable element (TE) gene models were annotated by mapping models from previously annotated Bd21 reference genomes [30, 35]. We conducted a basic gene mapping using GMAP (v2020-03–12) software with the following parameters: "-n 0 –nofails –split-large-introns –no-chimeras –gff3-add-separators = 0" [75]. RepeatMasker (v4.1.0; http://www.repeatmasker.org/) was used to identify telomeres on each chromosome of the genome, using the parameters: "-pa 4 -poly -html -gff".

De novo transposable element (TE) annotation using EDTA (v2.0.0) were performed with the parameters: "–sensitive 1 –anno 1 –evaluate 1" [76]. Subsequently, based on the results obtained from EDTA, we employed TEsorter (v1.4.6) for the identification of subfamily-level transposable elements [77, 78]. We also conducted a count of the copy numbers for centromere-specific transposable elements (CRM).

### Synteny analysis

Syntenic blocks were identified using the default parameters in the jcvi software [79]. Genes were used as queries to search for the best matching pairs in the different genomes. In order to assess the assembly of centromere regions, we conducted sequence alignments of three genome sets using the MUMmer (v4.0.0beta2) software [80]. The alignment process utilized the following parameters: "nucmer –maxmatch -l 100 -c 500". Subsequently, we applied R scripts to filter and visualize the short sequences. The genome sets included assembled IBd483-CEN and ABR113 (v1.1, https://phytozome-next.jgi.doe.gov/info/Bhybridum_v1_1); Bd21-CEN and Bd21, (v3.1, https://phytozome-next.jgi.doe.gov/info/Bdistachyon_v3_1), and Bst99-CEN and ABR114 (v1.1, https://phytozome-next.jgi.doe.gov/info/Bstacei_v1_1) [30, 35].

### Identification of structure variations

The three new assembled genome datasets were aligned with their previously references with the minimap2 tool (version 2.23) using the parameters: "-ax asm5 –eqx -t 6" [81]. Following the alignment, syri (version 1.1) was used to pinpoint the Inversions and Translocations [82]. Anchrowave (version 1.2.1) was employed to detect the variants across the three genomes using the "proali" alignment method, accompanied by parameters "-R 1 -Q 1" [83]. Furthermore, Assemblytics (version 1.2) was utilized for calling structural variations including deletions and insertions within the three genome sets [84].

### ChIP library preparation and ChIP-seq analysis

About 10–20 g leaf tissue was harvested and frozen in liquid nitrogen, then finely ground with pre-chilled mortars and pestles. The native ChIP protocol was adopted from a previously described method with micrococcal nuclease digestion of the DNA [85]. The anti-rabbit CENH3 polyclonal antibodies were raised against the peptides corresponding to the shared C-terminus of *Brachypodium* CENH3 proteins (KDIQLARRISGHRGY). The polyclonal antibodies were generated with the previous described method [86] provided by GL Biochem (Shanghai) Ltd. The digested chromatin without CENH3 enrichment was treated as Input. ChIPed and Input DNA were then used for preparing Illumina sequencing libraries to generate 101-nucleotide paired-end reads.

The raw paired-end reads were filtered using fastp for quality profiling [87], and the trimmed reads were mapped to the corresponding reference genomes with bwa mem method [88]. The centromere size was defined as the length of the DNA regions with the highest CENH3 intensity, and the enrichment level was defined as the ratio of the mean CENH3 intensity in centromere region to the genome-wide average intensity. The ChIP-seq signal distribution on CentBd/CentBs or CRM repeats was analyzed by using deepTools, and MetaProfiles were generated using the plotProfile function of deeptools [89]. Artificial tetraploid reference genome was generated by combining Bd21-CEN and Bst99-CEN reference genomes. To compare centromere changes, the ChIP-seq reads from tetraploid lines were mapped to the merged reference genome, and ChIP-seq reads from diploid lines were also mapped conversely to the subgenomes of IBd483-CEN reference genome, respectively.

### Identification and characterization of centromeric repeat sequences

Repetitive clusters were determined with the RepeatExplore2 website tool for each sample using the Input-seq reads [90]. The enrichment on each repeat cluster was calculated with the relative numbers of the reads located in CENH3 and Input data. The cluster with ratio value of CENH3/Input large than 2.6 and genome partitions great than 0.5% were treated as centromeric repeat clusters. Satellite repeat sequences were detected using Tandem Repeat Analyzer (TAR) [91].

### Phylogenetic tree of centromeric repeats in Brachypodium genomes

All the individual CentBd or CentBs satellite repeats were extracted using LASTZ (http://www.bx.psu.edu/~rsharris/lastz/) [92] for each *Brachypodium* assembled genomes. The repeat satellite units were used for further phylogenetic analysis. All the CRM elements were extracted from each assemblied genome. The CRM repeats were divided into the location inside or outside the core centromere. Multiple sequence alignments with different genomes or randomly selected subsamples were performed using MAFFT software, and phylogenetic analysis were generated with FastTree (-nt) software using the maximum likelihood method [93, 94]. The phylogenetic tree with branches as colored by sub/genomes was annotated and visualized with iTOL tool [95].

### Characterization of diversity within centromeric satellite repeats in Brachypodium genomes

The distribution of sequence identity of all satellite monomers to the CentBd or CentBs consensus was generated from the LASTZ results and plotted with ggplot2 using either boxplot or density plot. The comparisons of satellite sequence similarity within each sub/genome were performed as previously reported [20]. The dot plot of the centromere comparison between different sub/genomes or self-comparison was performed using the Redotable (https://www.bioinformatics.babraham.ac.uk/projects/redotable/). The pangenomes of *Brachypodium* were downloaded from available website (https://phytozome-next.jgi.doe.gov/brachypan) [30, 35]. These sequence data were produced by the US Department of Energy Joint Genome Institute.

The merged fragments from paired-end reads with length ranging from 146 to 166 bp were used to characterize diversity of satellites. The filtered fragments were BLASTed with a cut-off E-value of 10^−5^ to the CentBd or CentBs consensus sequence. The approach of principal component analysis (PCA) to reduce the space complexity of satellite repeats and to enable data visualization was adopted from other organism [38, 43]. The satellite repeats from reference genomes or high-throughput sequencing reads were used to generate a frequency table of 5-mer composition within a unit repeat, and the repeats were classified into groups based on (sub)genomes or a hierarchical clustering method (HCA) from first 100 principal components of the PCA [96]. A small randomly selected sequence samples were chosen to display the PCA projection on a two-dimensional plot by principal components 1 and 2. *T*-test, *P < 0.05, **P < 0.01, ***P < 0.001.

### FISH (fluorescence in situ hybridization)

Root tip cells and pollen mother cells of anther tissue from different lines were prepared for FISH as described previously [45]. The probes were designed to target the nucleotide differences between the CentBd and CentBs consensus sequences (CentBd_a1 + b1: TCAAATGGACACG + TGGTCTAGTCTTG; CentBs_a2 + b2: CCAAGTGCGCCCCA + TGTTCTCGTCGCG), and the resulting signal was labeled with either Alexa Fluor-594–5-dUTP (red) or Alexa Fluor-488-dUTP (green) for visualization. The chromosome preparations from different lines were exposed to equal amounts of probes and images using confocal microscopy (Cell Observer spinning disk confocal microscope, Zeiss) using the same exposure time. The resulting images were processed with Photoshop CS 6.0 (Adobe) to generate clear and accurate representations of the FISH signal.

### Supplementary Information

**Additional file 1.** This file contains Figures S1-S28.

**Additional file 2.** This file contains Tables S1-S17.

**Additional file 3.** Review history.

## Data Availability

The datasets generated during the current study are available in the Genome Sequence Archive (GSA) database in the BIG Data Center under accession numbers CRA011125 and project number PRJCA017149 [97]. The whole-genome sequence data reported in this paper have been deposited in the Genome Warehouse in National Genomics Data Center [98, 99], Beijing Institute of Genomics, and Chinese Academy of Sciences/China National Center for Bioinformation, under accession number GWHDUCW00000000 (IBd483), GWHDUCY00000000 (Bd21), and GWHDUCX00000000 (Bst99) that is publicly accessible at https://ngdc.cncb.ac.cn/gwh [100–102]. The ChIP-seq data generated in this study are available at the Gene Expression Omnibus (GEO) repository under accession number GSE157143 [103]. The ABR113, Bd21, and ABR114 genome sets are available at Phytozome (https://phytozome-next.jgi.doe.gov/) [30, 35, 104]. *Brachypodium* pangenomes used in this study were produced by the US Department of Energy Joint Genome Institute [30].
