# Supplementary material for: Three near-complete genome assemblies reveal substantial centromere dynamics from diploid to tetraploid in Brachypodium genus
Source: Genome Biol. 2024 Mar 4;25:63. doi: 10.1186/s13059-024-03206-w (PMC10910784; doi:10.1186/s13059-024-03206-w)
Supplement: Supplementary file 1 — Additional file 1. This file contains Figures S1-S28. [file 13059_2024_3206_MOESM1_ESM.pdf]

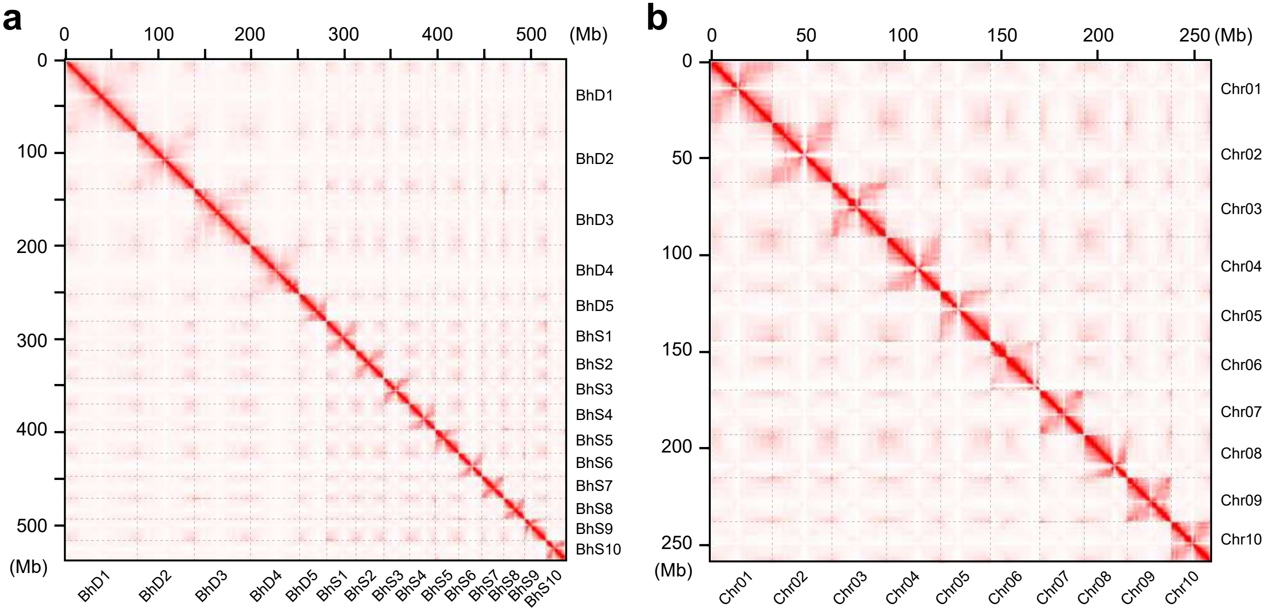
a
0
100
200
300
400
500
(Mb)
BhD1
BhD2
BhD3
BhD4
BhD5
BhS1
BhS2
BhS3
BhS4
BhS5
BhS6
BhS7
BhS8
BhS9
BhS10
b
0
50
100
150
200
250
(Mb)
Chr01
Chr02
Chr03
Chr04
Chr05
Chr06
Chr07
Chr08
Chr09
Chr10

Fig. S1 Genome-wide chromatin interactions in IBd483-CEN (a) and Bst99-CEN (b) genome assembly at 1000-Kb resolution.

The intensity of pixels represents the links between 1000-kb windows on all chromosomes. Darker red color indicates a higher contact probability, and the white space represents little or no contacts.

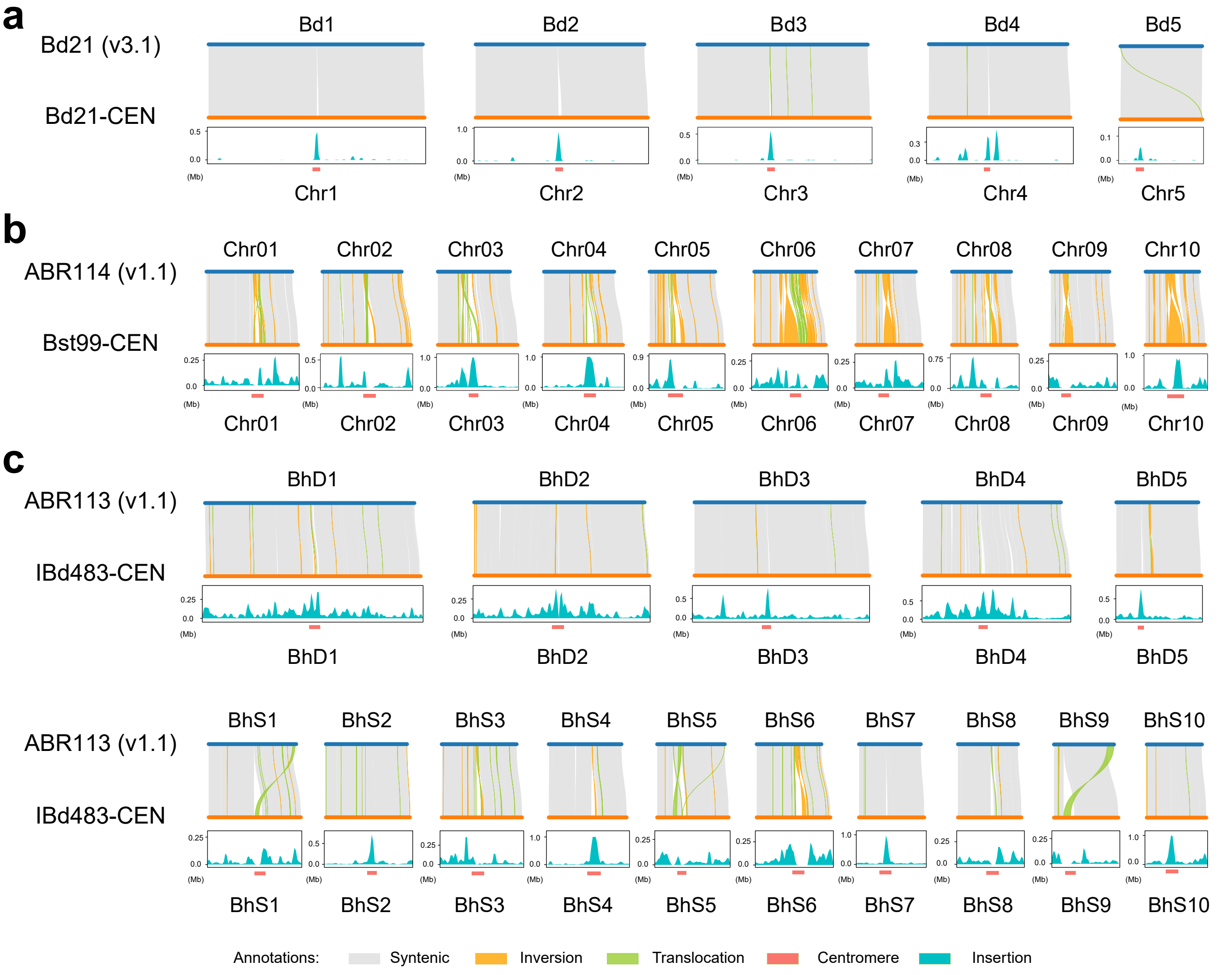
a
Bd21 (v3.1)
Bd21-CEN
Bd1
Bd2
Bd3
Bd4
Bd5
Chr1
Chr2
Chr3
Chr4
Chr5
(Mb)
b
ABR114 (v1.1)
Bst99-CEN
Chr01
Chr02
Chr03
Chr04
Chr05
Chr06
Chr07
Chr08
Chr09
Chr10
c
ABR113 (v1.1)
IBd483-CEN
BhD1
BhD2
BhD3
BhD4
BhD5
BhS1
BhS2
BhS3
BhS4
BhS5
BhS6
BhS7
BhS8
BhS9
BhS10
Annotations:
Syntenic
Inversion
Translocation
Centromere
Insertion

Fig. S2 Syntenic alignment and structural variation comparisons of new genome assemblies and their previous reference genomes.

a-c Alignments showing collinearity (gray), inversion (orange) and translocation (green) structural variations between Bd21 (v3.1) chromosomes and those of Bd21-CEN genome **(a)**, ABR114 (v1.1) chromosomes and those of Bst99-CEN genome **(b)**, ABR113 (v1.1) chromosomes and those of IBd483-CEN genome **(c)**. Distributions of insertion length were plotted (blue) along the chromosome in each genome. Centromere position was indicated with red box.

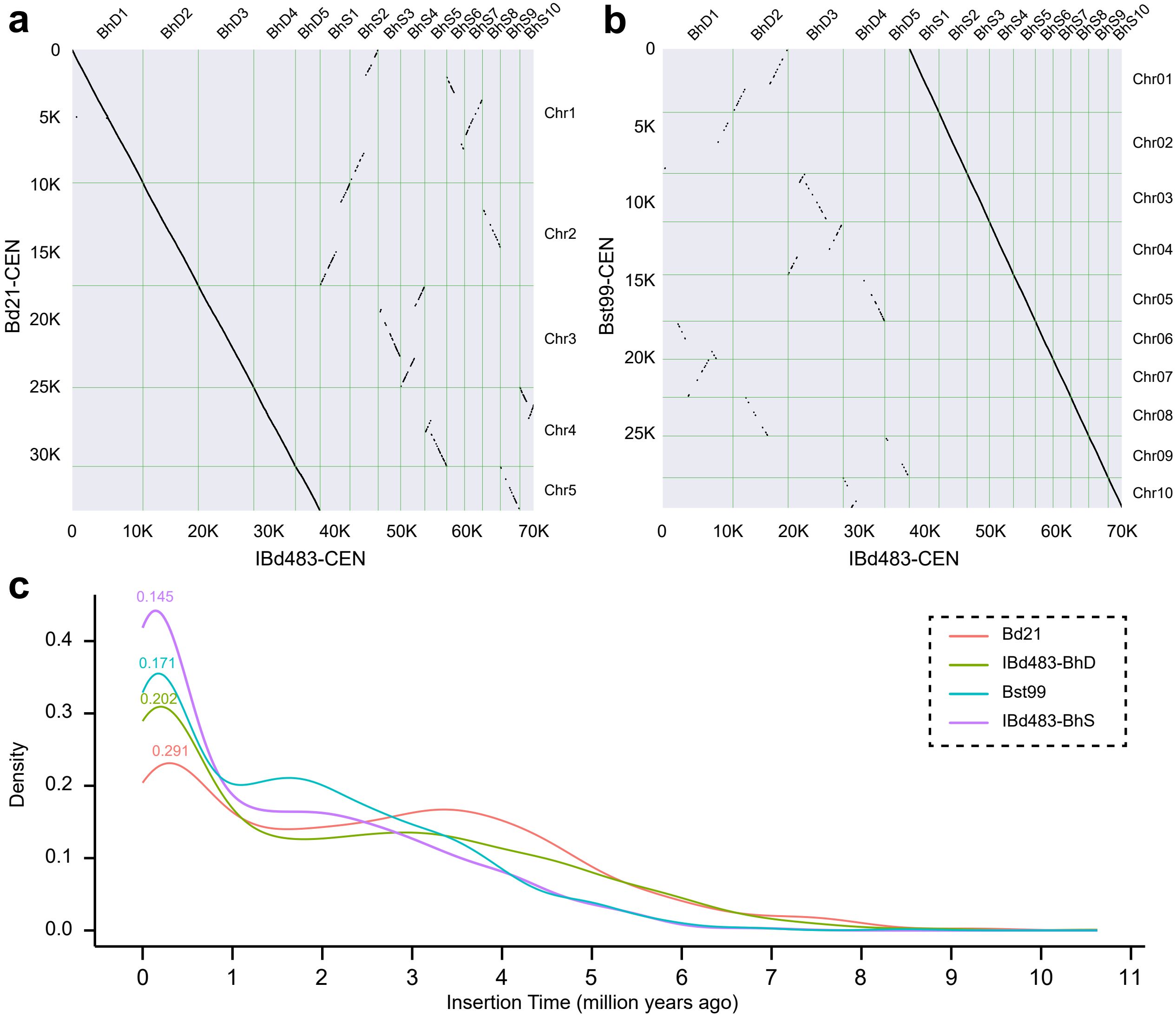

a
BhD1
BhD2
BhD3
BhD4
BhD5
BhS1
BhS2
BhS3
BhS4
BhS5
BhS6
BhS7
BhS8
BhS9
BhS10
Chr1
Chr2
Chr3
Chr4
Chr5
Bd21-CEN
IBd483-CEN
b
Chr01
Chr02
Chr03
Chr04
Chr05
Chr06
Chr07
Chr08
Chr09
Chr10
Bst99-CEN
IBd483-CEN
c
0.145
0.171
0.202
0.291
Bd21
IBd483-BhD
Bst99
IBd483-BhS
Density
Insertion Time (million years ago)

Fig. S3 The evolution of three species in *Brachypodium* genus.

a-b Alignments of IBd483-CEN chromosomes with those of Bd21-CEN **(a)** and Bst99-CEN **(b)**, respectively. The labels (0, 20 K, 40 K) on the x and y axes indicate the gene rank along the length of the chromosomes. **c** Distributions of intact LTR insertion time (million years ago) in three *Brachypodium* species.

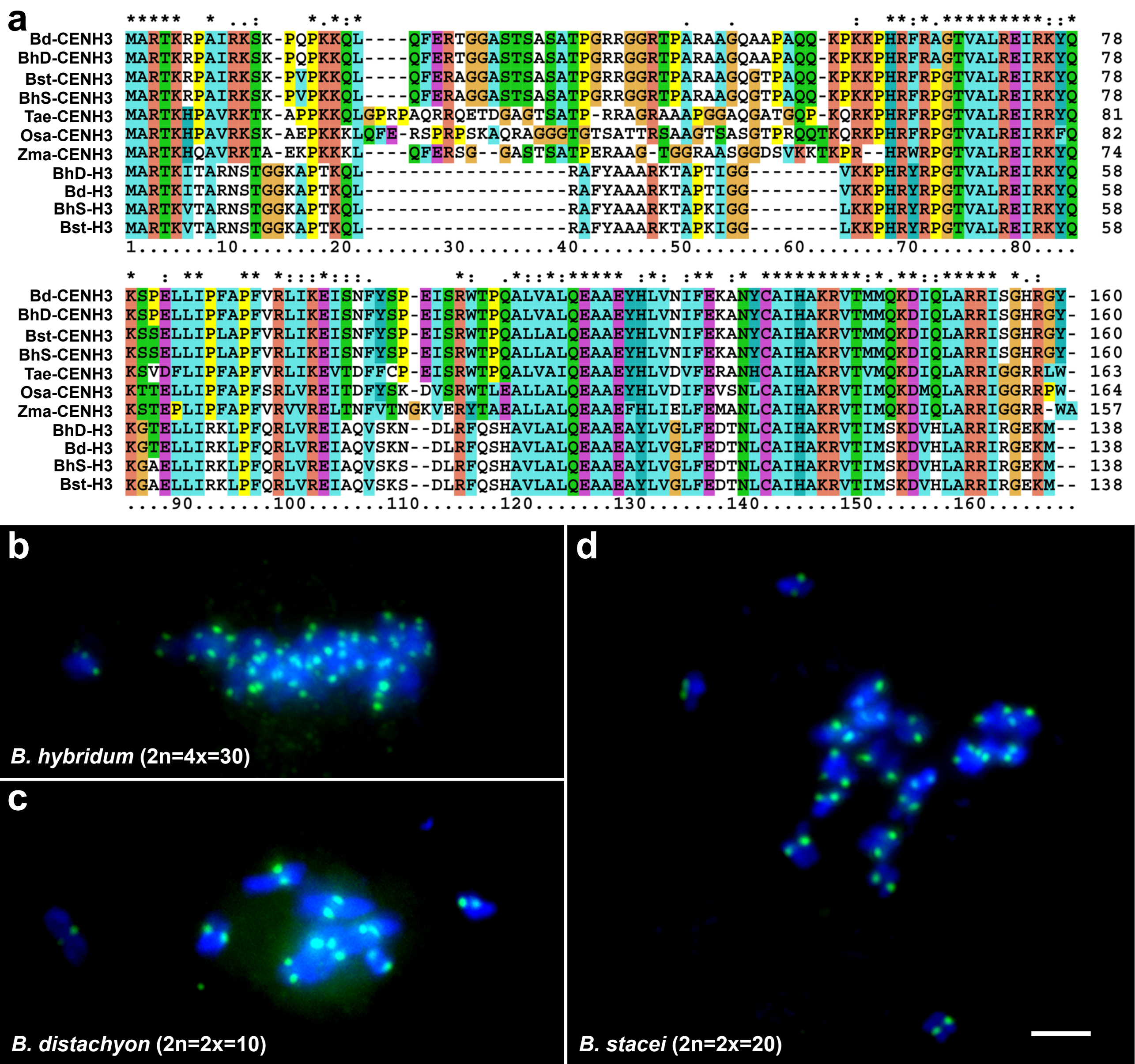
a
Bd-CENH3 MARTKRPAIRKSK-PQPKKQL----QFERTGGASTSASATPGRRGGRTPARAAGQAAPAQQ-KPKKPHRFRAGTVALREIRKYQ 78
BhD-CENH3 MARTKRPAIRKSK-PQPKKQL----QFERTGGASTSASATPGRRGGRTPARAAGQAAPAQQ-KPKKPHRFRAGTVALREIRKYQ 78
Bst-CENH3 MARTKRPAIRKSK-PVPKKQL----QFERAGGASTSASATPGRRGGRTPARAAGQGTPAQQ-KPKKPHRFRPGTVALREIRKYQ 78
BhS-CENH3 MARTKRPAIRKSK-PVPKKQL----QFERAGGASTSASATPGRRGGRTPARAAGQGTPAQQ-KPKKPHRFRPGTVALREIRKYQ 78
Tae-CENH3 MARTKHPAVRKTK-APPKKQLGPRPAQRRQETDGAGTSATP-RRAGRAAAPGGAQGATGQP-KQRKPHRFRPGTVALREIRRYQ 81
Osa-CENH3 MARTKHPAVRKSK-AEPKKKLQFE-RSPRPSKAQRAGGGTGTSATTRSAAGTSASGTPRQQTKQRKPHRFRPGTVALREIRKFQ 82
Zma-CENH3 MARTKHQAVRKTA-EKPKKKL----QFERSG--GASTSATPERAAG-TGGRAASGGDSVKKTKPR--HRWRPGTVALREIRKYQ 74
BhD-H3 MARTKITARNSTGGKAPTKQL------------------RAFYAAARKTAPTIGG--------VKKPHRYRPGTVALREIRKYQ 58
Bd-H3 MARTKITARNSTGGKAPTKQL------------------RAFYAAARKTAPTIGG--------VKKPHRYRPGTVALREIRKYQ 58
BhS-H3 MARTKVTARNSTGGKAPTKQL------------------RAFYAAARKTAPKIGG--------LKKPHRYRPGTVALREIRKYQ 58
Bst-H3 MARTKVTARNSTGGKAPTKQL------------------RAFYAAARKTAPKIGG--------LKKPHRYRPGTVALREIRKYQ 58
Bd-CENH3 KSPELLIPFAPFVRLIKEISNFYSP-EISRWTPQALVALQEAAEYHLVNIFEKANYCAIHAKRVTMMQKDIQLARRISGHRGY- 160
BhD-CENH3 KSPELLIPFAPFVRLIKEISNFYSP-EISRWTPQALVALQEAAEYHLVNIFEKANYCAIHAKRVTMMQKDIQLARRISGHRGY- 160
Bst-CENH3 KSSELLIPLAPFVRLIKEISNFYSP-EISRWTPQALLALQEAAEYHLVNIFEKANYCAIHAKRVTMMQKDIQLARRISGHRGY- 160
BhS-CENH3 KSSELLIPLAPFVRLIKEISNFYSP-EISRWTPQALLALQEAAEYHLVNIFEKANYCAIHAKRVTMMQKDIQLARRISGHRGY- 160
Tae-CENH3 KSVDFLIPFAPFVRLIKEVTDFFCP-EISRWTPQALVAIQEAAEYHLVDVFERANHCAIHAKRVTVMQKDIQLARRIGGRRLW- 163
Osa-CENH3 KTTELLIPFAPFSRLVREITDFYSK-DVSRWTLEALLALQEAAEYHLVDIFEVSNLCAIHAKRVTIMQKDMQLARRIGGRRPW- 164
Zma-CENH3 KSTEPLIPFAPFVRVVRELTNFVTNGKVERYTAEALLALQEAAEFHLIELFEMANLCAIHAKRVTIMQKDIQLARRIGGRR-WA 157
BhD-H3 KGTELLIRKLPFQRLVREIAQVSKN--DLRFQSHAVLALQEAAEAYLVGLFEDTNLCAIHAKRVTIMSKDVHLARRIRGEKM-- 138
Bd-H3 KGTELLIRKLPFQRLVREIAQVSKN--DLRFQSHAVLALQEAAEAYLVGLFEDTNLCAIHAKRVTIMSKDVHLARRIRGEKM-- 138
BhS-H3 KGAELLIRKLPFQRLVREIAQVSKS--DLRFQSHAVLALQEAAEAYLVGLFEDTNLCAIHAKRVTIMSKDVHLARRIRGEKM-- 138
Bst-H3 KGAELLIRKLPFQRLVREIAQVSKS--DLRFQSHAVLALQEAAEAYLVGLFEDTNLCAIHAKRVTIMSKDVHLARRIRGEKM-- 138
b
B. hybridum (2n=4x=30)
c
B. distachyon (2n=2x=10)
d
B. stacei (2n=2x=20)

Fig. S4 Characterization of CENH3 proteins in three *Brachypodium* species.

a Multiple alignments of CENH3 and H3 protein homologs from different plant lineages, including *B. distachyon* (Bd), *B. stacei* (Bst), *B. hybridum* (Bhy), *Triticum aestivum* (Tae), *Oryza sativa* (Osa) and *Zea mays* (Zma). The red line on the amino acids indicates the peptide used to yield anti-CENH3 antibodies. **b-d** Immunostaining of somatic metaphase chromosomes in different lines using the anti-CENH3 antibodies. Scale Bar = 10 μm. CENH3 signals are colored in green. Chromosomes counterstained with DAPI are in blue.

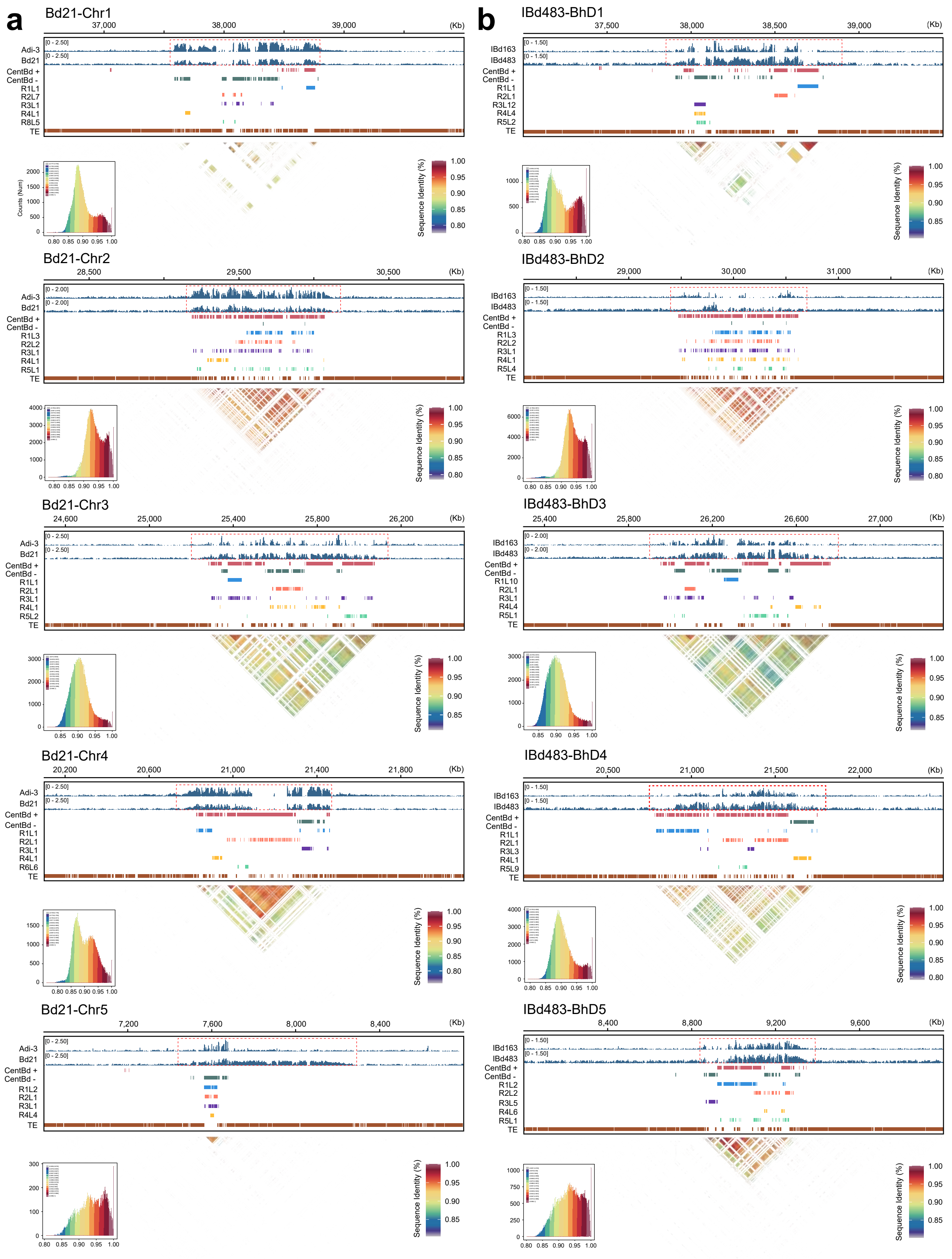

a
Bd21-Chr1
37,000
38,000
39,000
(Kb)
Adi-3
Bd21
[0 - 2.50]
CentBd +
CentBd -
R1L1
R2L7
R3L1
R4L1
R8L5
TE
Counts (Num)
Sequence Identity (%)
Bd21-Chr2
28,500
29,500
30,500
[0 - 2.00]
R1L3
R2L2
R3L1
R4L1
R5L1
Bd21-Chr3
24,600
25,000
25,400
25,800
26,200
R1L1
R2L1
R3L1
R4L1
R5L2
Bd21-Chr4
20,200
20,600
21,000
21,400
21,800
R1L1
R2L1
R3L1
R4L1
R6L6
Bd21-Chr5
7,200
7,600
8,000
8,400
R1L2
R2L1
R3L1
R4L4
b
IBd483-BhD1
37,500
38,000
38,500
39,000
IBd163
IBd483
[0 - 1.50]
R1L1
R2L1
R3L12
R4L4
R5L2
IBd483-BhD2
29,000
30,000
31,000
R1L3
R2L2
R3L1
R4L1
R5L4
IBd483-BhD3
25,400
25,800
26,200
26,600
27,000
[0 - 2.00]
R1L10
R2L1
R3L1
R4L4
R5L1
IBd483-BhD4
20,500
21,000
21,500
22,000
R1L1
R2L1
R3L3
R4L1
R5L9
IBd483-BhD5
8,400
8,800
9,200
9,600
R1L2
R2L2
R3L5
R4L6
R5L1

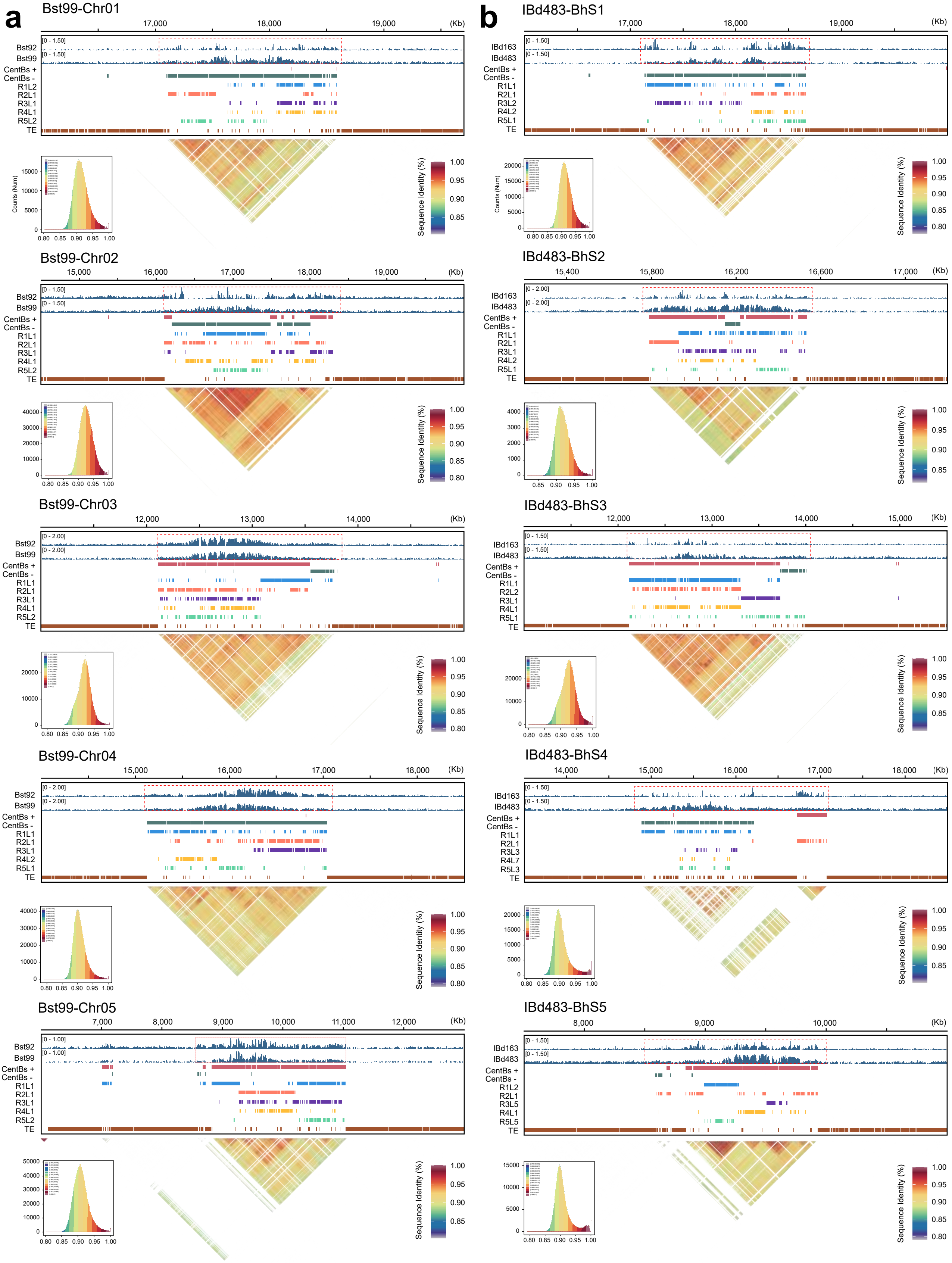

a
Bst99-Chr01
17,000
18,000
19,000
(Kb)
[0 - 1.50]
Bst92
Bst99
CentBs +
CentBs -
R1L2
R2L1
R3L1
R4L1
R5L2
TE
Counts (Num)
Sequence Identity (%)
Bst99-Chr02
15,000
16,000
17,000
18,000
19,000
R1L1
R2L1
R3L1
R4L1
R5L2
Bst99-Chr03
12,000
13,000
14,000
[0 - 2.00]
Bst99-Chr04
15,000
16,000
17,000
18,000
R4L2
R5L1
Bst99-Chr05
7,000
8,000
9,000
10,000
11,000
12,000
[0 - 1.00]
b
IBd483-BhS1
IBd163
IBd483
R3L2
R4L2
R5L1
IBd483-BhS2
15,400
15,800
16,200
16,600
17,000
IBd483-BhS3
15,000
R2L2
IBd483-BhS4
14,000
R3L3
R4L7
R5L3
IBd483-BhS5
R1L2
R3L5
R5L5
0.80
0.85
0.90
0.95
1.00

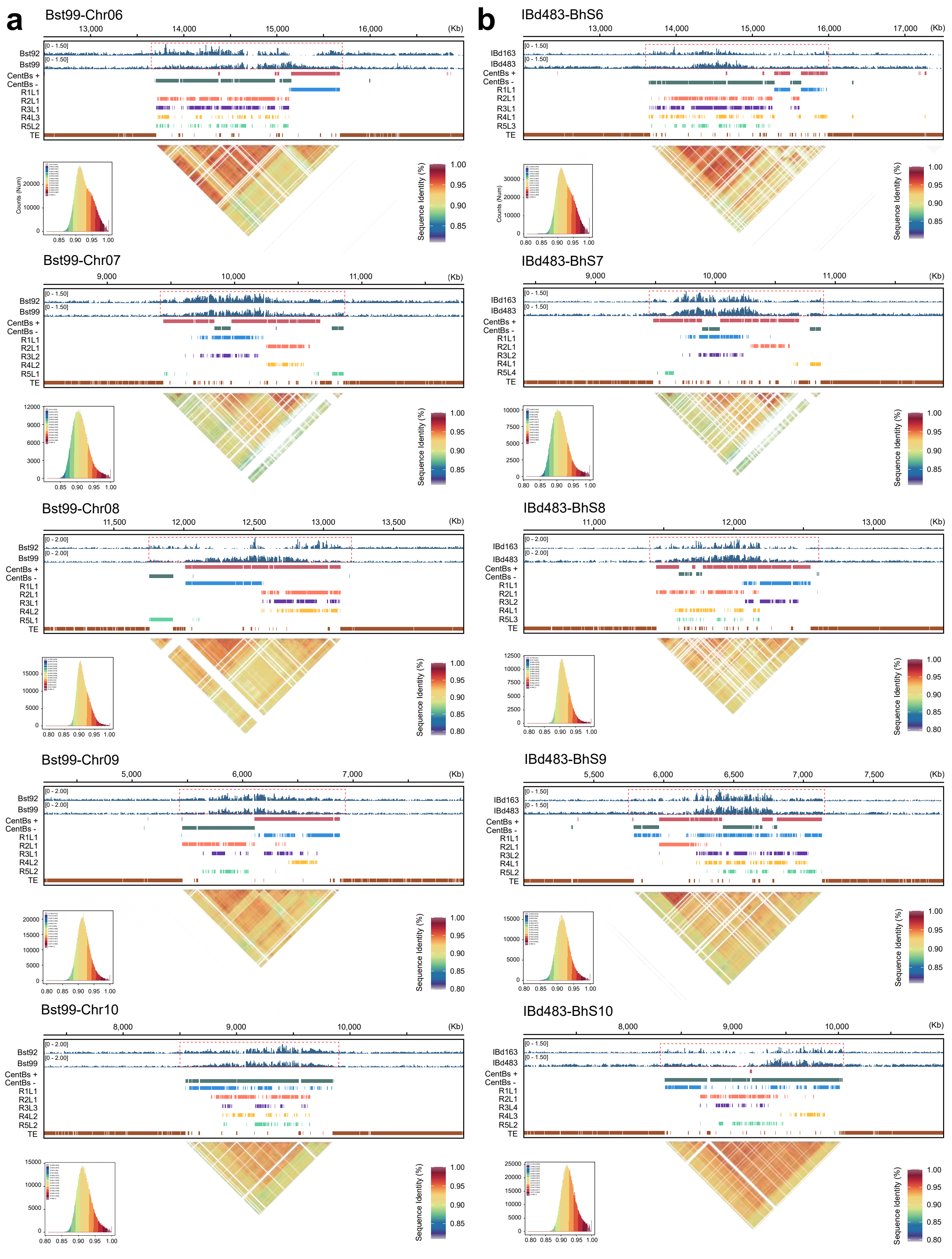

a
Bst99-Chr06
Bst99-Chr07
Bst99-Chr08
Bst99-Chr09
Bst99-Chr10
b
IBd483-BhS6
IBd483-BhS7
IBd483-BhS8
IBd483-BhS9
IBd483-BhS10
Bst92
Bst99
IBd163
IBd483
CentBs +
CentBs -
R1L1
R2L1
R3L1
R3L2
R3L3
R3L4
R4L1
R4L2
R4L3
R5L1
R5L2
R5L3
R5L4
TE
(Kb)
Counts (Num)
Sequence Identity (%)

Fig. S5-S7 Comprehensive map of centromeres in *Brachypodium* genus.

CENH3 enrichment [log_2(ChIP/input)] plotted over centromeres between Bd21-CEN and BhD subgenome of IBd483-CEN **(Fig. S5)**, and between Bst99-CEN and BhS subgenome of IBd483-CEN **(Fig. S6-S7)**. The layers demonstrate the CentBd or CentBs distribution with forward (red) or reverse (green) strand orientations, the structure of High-order repeats (HORs, with the top 5 frequent HORs arranged in descending order, using colors blue, orange yellow, purple, earthy yellow, and light green, the numbers after "R" represents ranked HORs, the number after "L" represents the length of HOR units in monomer pattern), Transposable Elements (TEs, brown), respectively. Notably, Chr5 in the Bd21-CEN genome detected only four frequent HORs. The heatmaps display the pairwise similarity of CentBd satellite repeats within all non-overlapping 1-kb regions of each chromosome. The histogram illustrates the statistics distribution of the similarity of these satellite repeats.

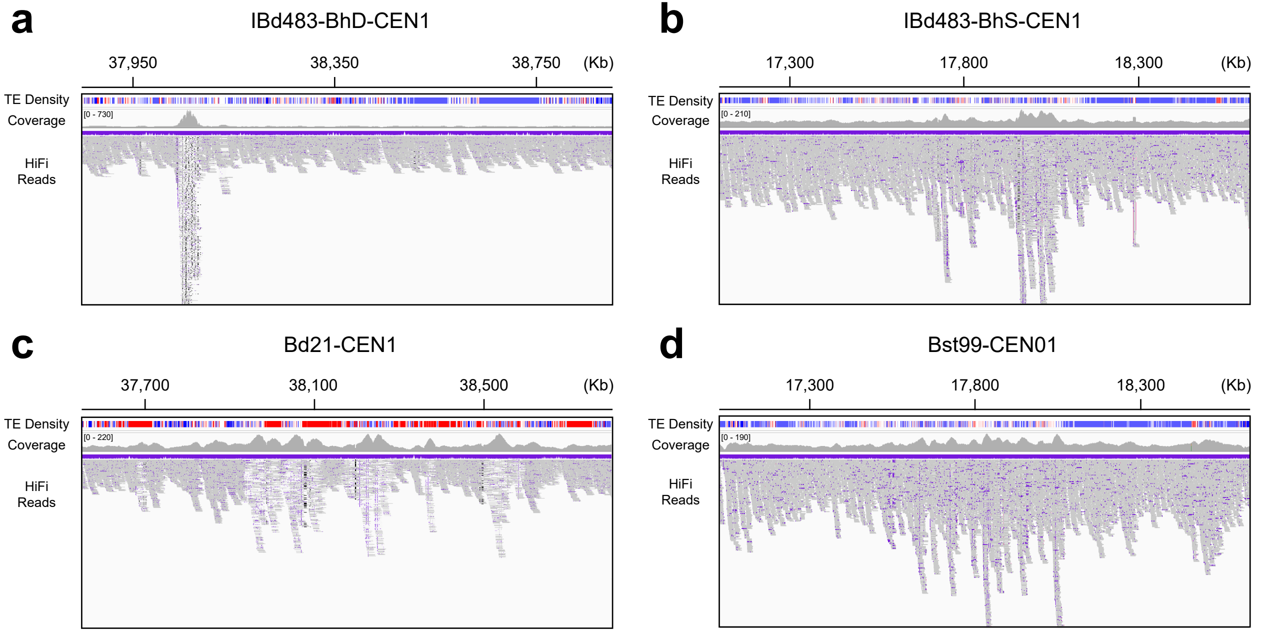
a
IBd483-BhD-CEN1
37,950
38,350
38,750
(Kb)
TE Density
Coverage
[0 - 730]
HiFi
Reads
b
IBd483-BhS-CEN1
17,300
17,800
18,300
(Kb)
TE Density
Coverage
[0 - 210]
HiFi
Reads
c
Bd21-CEN1
37,700
38,100
38,500
(Kb)
TE Density
Coverage
[0 - 220]
HiFi
Reads
d
Bst99-CEN01
17,300
17,800
18,300
(Kb)
TE Density
Coverage
[0 - 190]
HiFi
Reads

Fig. S8 IGV screenshots in the centromere regions of three *Brachypodium* species. **a** IGV screenshot shows the centromere region of BhD1 (37,850,000-38,900,000) in IBd483-CEN genome. The top track shows the density of TE, quantified as the number of transposons per kilobase (1-kb). Upon integration into the IGV platform for heatmap scale normalization, data values equal to or below -1.5 are represented in a deep blue hue, whereas values equal to or surpassing 1.5 are depicted in a deep red. Values spanning from -0.1 to 0.1 are correspondingly mapped onto a white color spectrum, providing a nuanced visualization of the genomic data. The middle track displays the coverage of PacBio HiFi reads. The alignment of PacBio HiFi reads (lower track) is shown. The purple marks indicate insertion. **b** The centromere region of BhS1 (17,100,000-18,700,000 in IBd483-CEN genome. **c** The centromere region of Chr1 (37,550,000-38,800,000) in Bd21-CEN genome. **d** The centromere region of Chr01 (17,035,000-18,630,000) in Bst99-CEN genome.

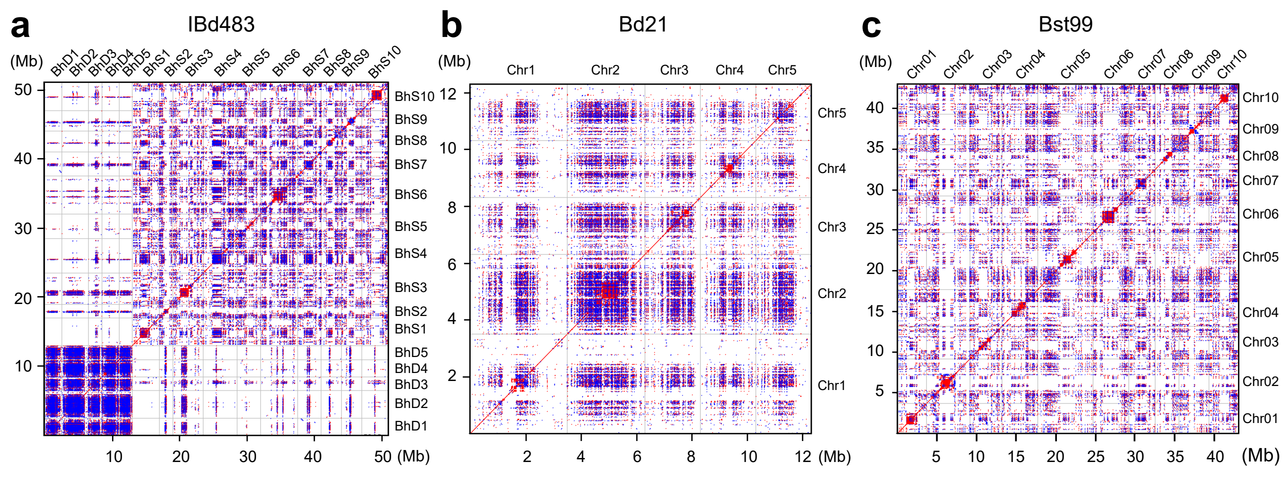
a
IBd483
(Mb)
BhD1
BhD2
BhD3
BhD4
BhD5
BhS1
BhS2
BhS3
BhS4
BhS5
BhS6
BhS7
BhS8
BhS9
BhS10
b
Bd21
Chr1
Chr2
Chr3
Chr4
Chr5
c
Bst99
Chr01
Chr02
Chr03
Chr04
Chr05
Chr06
Chr07
Chr08
Chr09
Chr10

Fig. S9 Dot plots comparing the centromeres within each assembled genome using a search window of 156-bp in *Brachypodium genus*.

a Dot plots comparing the fifteen centromeres of IBd483-CEN assembled genome (BhD1: 37,000,000-39,500,000, BhD2: 28,000,000-32,000,000, BhD3: 25,300,000-27,300,000, BhD4: 20,000,000-22,500,000, BhD5: 8,000,000-10,000,000, BhS1: 16,000,000-20,000,000, BhS2: 15,200,000-17,200,000, BhS3: 11,000,000-15,500,000, BhS4: 13,500,000-18,500,000, BhS5: 7,500,000-11,000,000, BhS6: 12,000,000-17,500,000, BhS7: 8,400,000-11,900,000, BhS8: 10,500,000-13,500,000, BhS9: 5,000,000-8,000,000, BhS10: 7,000,000-11,000,000). **b** Dot plots comparing the five centromeres of Bd21-CEN assembled genome (Chr1: 36,500,000-40,000,000, Chr2: 28,200,000-31,000,000, Chr3: 24,500,000-26,500,000, Chr4: 20,100,000-22,100,000, Chr5: 6,800,000-8,800,000). **c** Dot plots comparing the five centromeres of Bst99-CEN assembled genome (Chr01: 16,000,000-19,700,000, Chr02: 14,500,000-20,000,000, Chr03: 11,000,000-15,000,000, Chr04: 14,000,000-18,500,000, Chr05: 6,000,000-13,000,000, Chr06: 12,500,000-17,000,000, Chr07: 8,500,000-11,800,000, Chr08: 11,000,000-14,000,000, Chr09: 4,200,000-8,000,000, Chr10: 7,300,000-11,000,000). Red and blue indicate forward- and reverse-strand similarity, respectively.

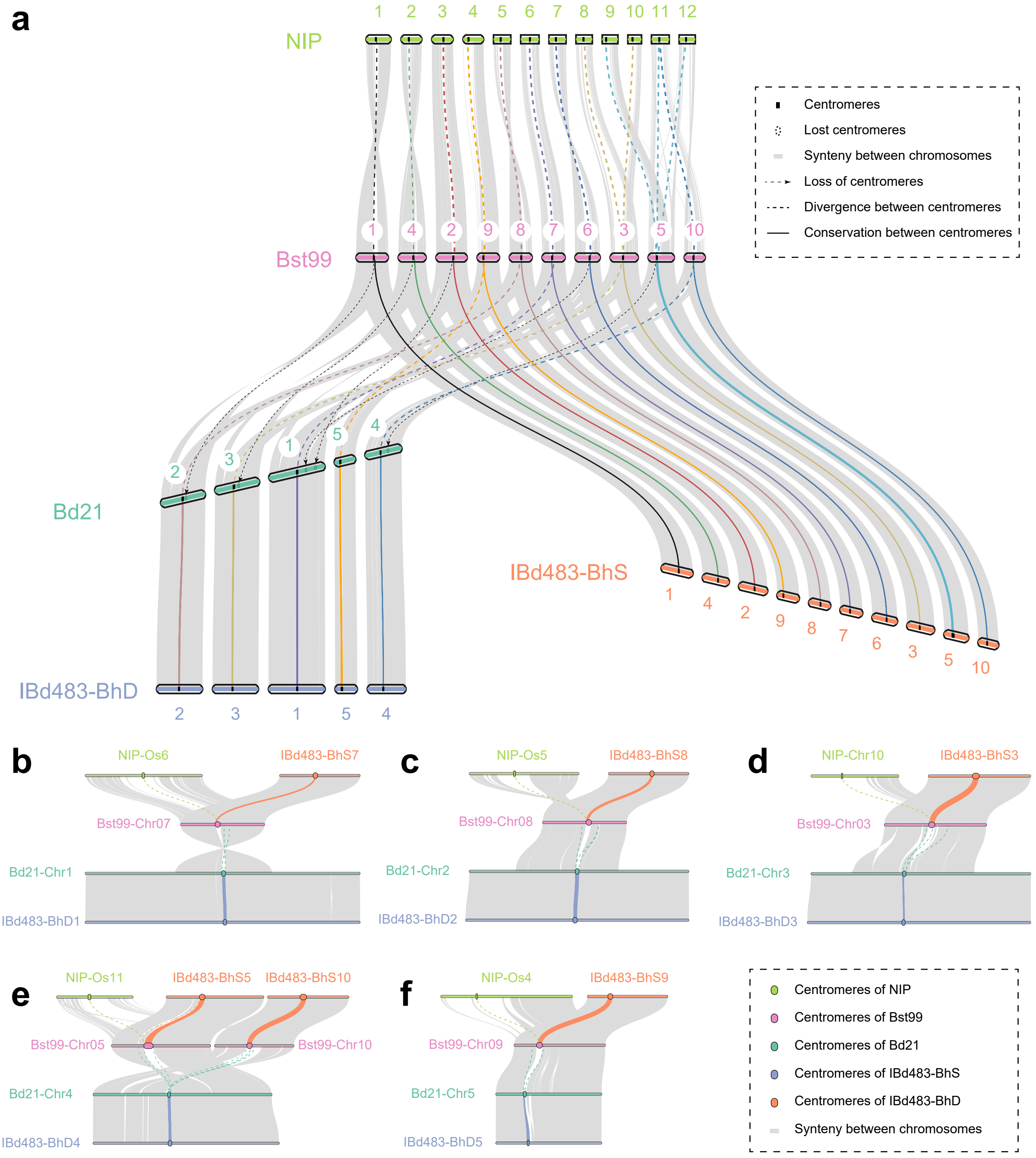

a
NIP
1 2 3 4 5 6 7 8 9 10 11 12
Bst99
1 4 2 9 8 7 6 3 5 10
Bd21
2 3 1 5 4
IBd483-BhS
1 4 2 9 8 7 6 3 5 10
IBd483-BhD
2 3 1 5 4
Centromeres
Lost centromeres
Synteny between chromosomes
Loss of centromeres
Divergence between centromeres
Conservation between centromeres
b
NIP-Os6
IBd483-BhS7
Bst99-Chr07
Bd21-Chr1
IBd483-BhD1
c
NIP-Os5
IBd483-BhS8
Bst99-Chr08
Bd21-Chr2
IBd483-BhD2
d
NIP-Chr10
IBd483-BhS3
Bst99-Chr03
Bd21-Chr3
IBd483-BhD3
e
NIP-Os11
IBd483-BhS5
IBd483-BhS10
Bst99-Chr05
Bst99-Chr10
Bd21-Chr4
IBd483-BhD4
f
NIP-Os4
IBd483-BhS9
Bst99-Chr09
Bd21-Chr5
IBd483-BhD5
Centromeres of NIP
Centromeres of Bst99
Centromeres of Bd21
Centromeres of IBd483-BhS
Centromeres of IBd483-BhD
Synteny between chromosomes

Fig. S10 Syntenic chromosome relationships and centromere evolution among *O. sativa*, *B. distachyon*, *B. stacei*, and *B. hybridum* genome.

a Syntenic chromosome relationships reveal that *B. distachyon* (Bd21) chromosomes evolved from an ancestral chromosome similar to *B. stacei* (Bst99) through a series of nested insertions of whole chromosomes into centromere of Chr1, Chr2, and Chr3, fission of two chromosomes into Chr4, and collinearity of one chromosome with Chr5. Bst99 chromosomes evolved from an ancestor similar to rice (*O. sativa,* NIP) through nested insertions into centromeric regions of Chr03 and Chr05, fusion of three chromosomes to Chr10, and collinearity of one chromosome with other chromosomes. Dotted lines denote centromere divergences between NIP and Bst99 or between Bst99 and Bd21, while dashed line with arrows represent centromere loss during the nested chromosome insertions or chromosome fissions between Bst99 and Bd21. Solid lines indicate that the centromeres of Bd21 and Bst99 chromosomes are collinear with the centromeres of the D- and S- subgenomes of *B. hybridum* (IBd483), respectively. Gray boxes denote synteny between other chromosome regions. **b-f** Detailed representation of the syntenic relationship between the centromeres of five Bd21 chromosomes and the corresponding Bst99 chromosomes, the subgenome of IBd483 chromosomes, and ancestral rice (NIP) chromosomes. Filled circles on each chromosome pinpoint the location of centromere. Dotted light green lines signify almost no collinearity between the centromeres of NIP and Bst99, and dotted dark green lines indicate slight collinearity between the centromeres of Bd21 and Bst99. Solid blue and orange lines indicate conserved centromeres between Bd21 or Bst99 and their corresponding subgenomes of IBd483.

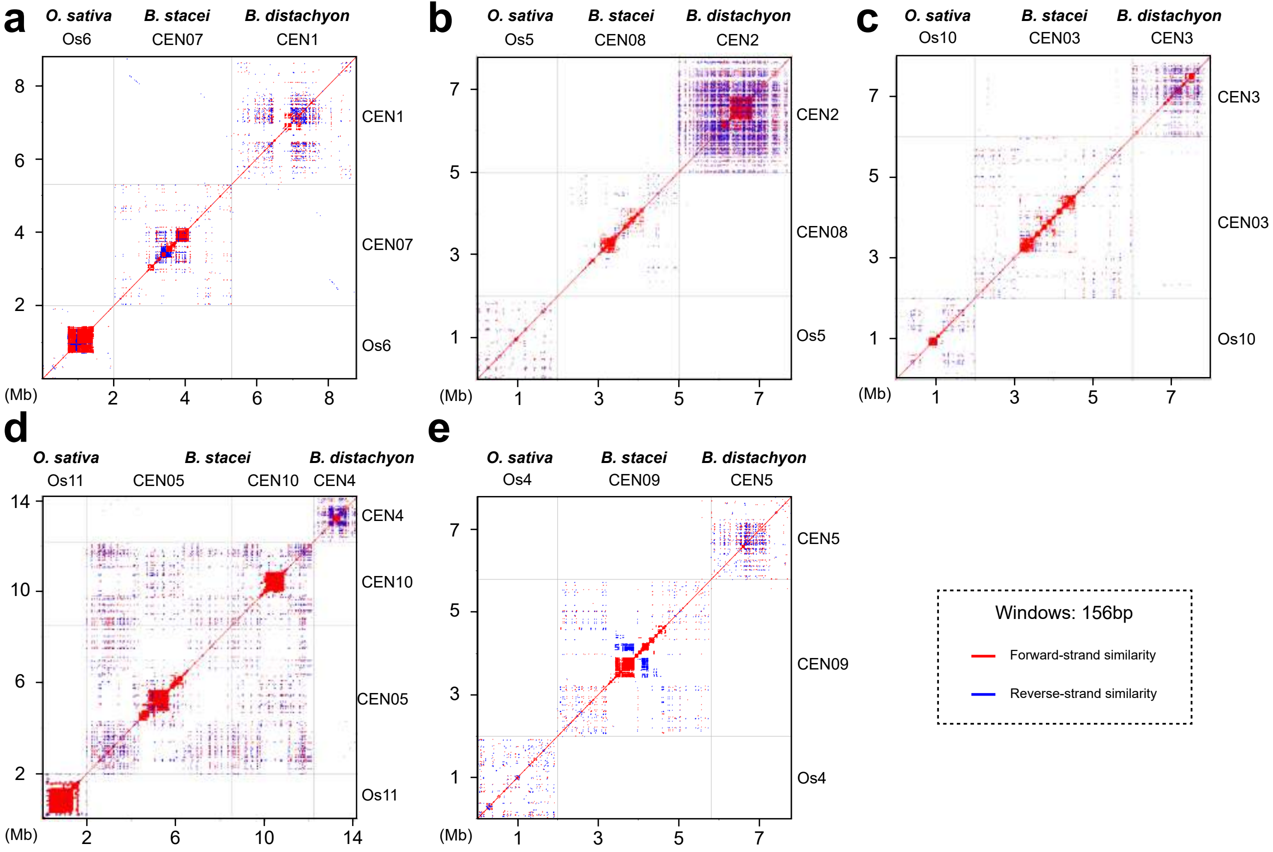

a
O. sativa
B. stacei
B. distachyon
Os6
CEN07
CEN1
(Mb)
b
O. sativa
B. stacei
B. distachyon
Os5
CEN08
CEN2
c
O. sativa
B. stacei
B. distachyon
Os10
CEN03
CEN3
d
O. sativa
B. stacei
B. distachyon
Os11
CEN05
CEN10
CEN4
e
O. sativa
B. stacei
B. distachyon
Os4
CEN09
CEN5
Windows: 156bp
Forward-strand similarity
Reverse-strand similarity

Fig. S11 Dot plots comparing syntenic centromeres among *O. sativa*, *B. stacei*, and *B. distachyon* genome using a search window of 156-bp.

The syntenic chromosomes relationships among *O. sativa*, *B. stacei*, and *B. distachyon* are illustrated in Figure S10. **a** Os6 (*O. sativa*, 14,900,000-16,900,000)-CEN07 (*B. stacei*, 8,500,000-11,800,000)-CEN1 (*B. distachyon*, 36,500,000-40,000,000); **b** Os5 (11,600,000-13,600,000)-CEN08 (11,000,000-14,000,000)-CEN2 (28,200,000-31,000,000); **c** Os10 (7,500,000-9,500,000)-CEN03 (11,000,000-15,000,000)-CEN3 (24,500,000-26,500,000); **d** Os11 (12,600,000-14,600,000)-CEN05/CEN10 (CEN05: 6,500,000-13,000,000, CEN10: 7,300,000-11,000,000)-CEN4 (20,100,000-22,100,000); **e** Os4 (9,000,000-11,000,000)-CEN09 (4,200,000-8,000,000)-CEN5 (6,800,000-8,800,000). Select the complete Nipponbare reference genome (NIP) as *O. sativa* genome (http://www.ricesuperpir.com/web/nip). Red and blue indicate forward- and reverse-strand similarity, respectively.

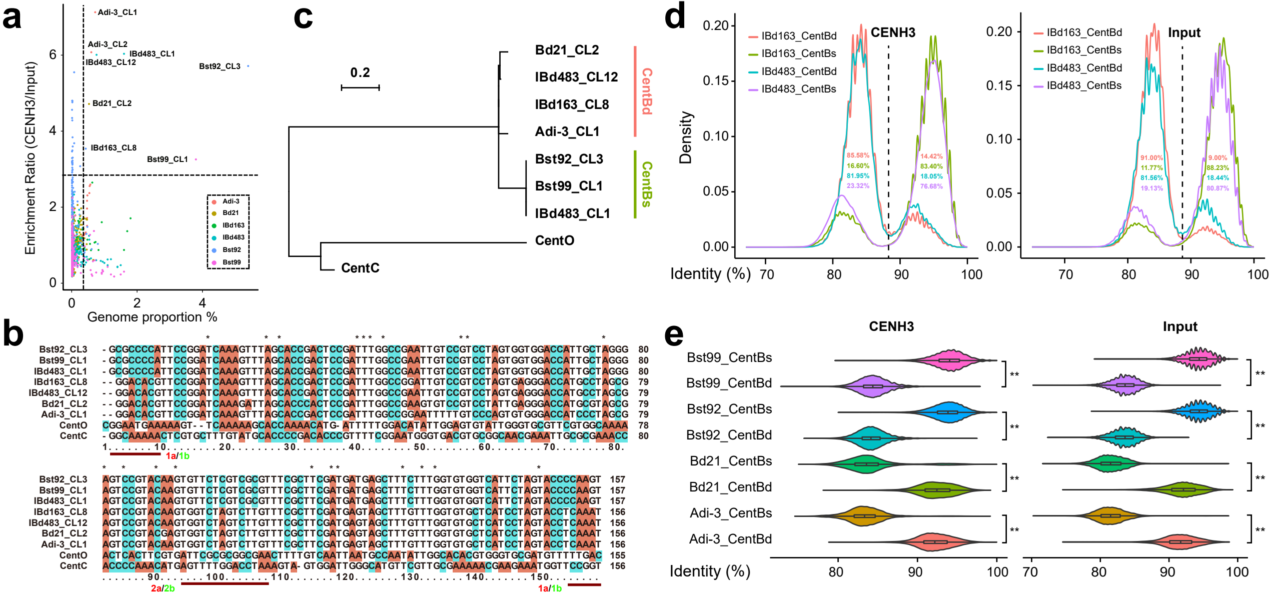
a
Enrichment Ratio (CENH3/Input)
Genome proportion %
Adi-3_CL1
Adi-3_CL2
IBd483_CL1
IBd483_CL12
Bst92_CL3
Bd21_CL2
IBd163_CL8
Bst99_CL1
Adi-3
Bd21
IBd163
IBd483
Bst92
Bst99
0
2
4
6
b
Bst92_CL3
Bst99_CL1
IBd483_CL1
IBd163_CL8
IBd483_CL12
Bd21_CL2
Adi-3_CL1
CentO
CentC
1a/1b
2a/2b
1a/1b
c
0.2
Bd21_CL2
IBd483_CL12
IBd163_CL8
Adi-3_CL1
Bst92_CL3
Bst99_CL1
IBd483_CL1
CentO
CentC
CentBd
CentBs
d
Density
Identity (%)
CENH3
Input
IBd163_CentBd
IBd163_CentBs
IBd483_CentBd
IBd483_CentBs
85.58%
16.60%
81.95%
23.32%
14.42%
83.40%
18.05%
76.68%
91.00%
11.77%
81.56%
19.13%
9.00%
88.23%
18.44%
80.87%
0.00
0.05
0.10
0.15
0.20
70
80
90
100
e
CENH3
Input
Bst99_CentBs
Bst99_CentBd
Bst92_CentBs
Bst92_CentBd
Bd21_CentBs
Bd21_CentBd
Adi-3_CentBs
Adi-3_CentBd
**
Identity (%)
70
80
90
100

Fig. S12 Identification and characterization of centromeric repeat sequences in *Brachypodium genus*.

a Identification of centromere-enriched repeat clusters in different lines of *B. hybridum, B. distachyon* and *B. stacei*. Dot plot shows the distribution of repeat clusters with genomic proportion (%, x axis) and their enrichment ratio (ChIP/Input, y axis). Only the repeat clusters with genome proportions >0.50% and the enrichment ratio >2.6-fold were selected as centromeric repeat. IBd163_CL8, IBd483_CL1 and IBd483_CL12 represent the clusters of satellite repeats identified from IBd163 and IBd483 lines using RepeatExplore. **b** Multiple alignment of centromeric satellite clusters identified from different lines in the *Brachypodium* genus. CentO and CentC indicate the centromeric satellite repeats from rice and maize. 1a/1b and 2a/2b labels represent the different nucleotides underlying the divergent repeats. **c** Phylogenetic tree of alignment centromeric satellite repeats in different plant species. **d** Sequence identity of merged fragments from ChIP-seq and Input-seq to the CentBd or CentBs satellite consensus sequence, as sampled in IBd163 and IBd483 lines. **e** Sequence identity of merged fragments from ChIP-seq and Input-seq reads to CentBd or CentBS consensus sequence, as sampled in Adi-3, Bd21, Bst92 and Bst99 lines. The x axis represents the sequence identity; the y axis represents the proportion of the satellites repeats. (T-test, ** $P<0.01$).

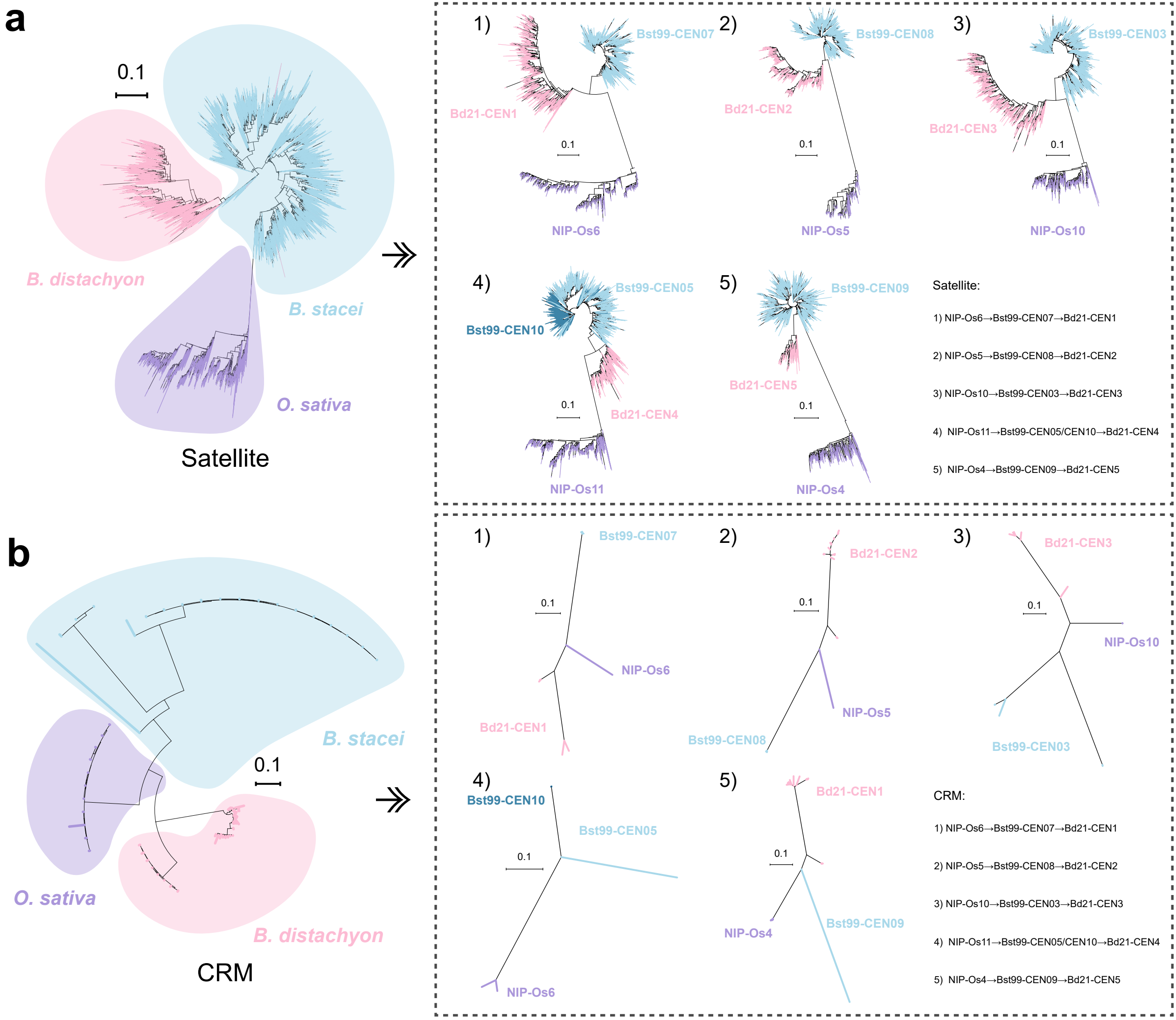
a
0.1
B. distachyon
B. stacei
O. sativa
Satellite
1)
Bd21-CEN1
Bst99-CEN07
NIP-Os6
2)
Bst99-CEN08
Bd21-CEN2
NIP-Os5
3)
Bst99-CEN03
Bd21-CEN3
NIP-Os10
4)
Bst99-CEN05
Bst99-CEN10
Bd21-CEN4
NIP-Os11
5)
Bst99-CEN09
Bd21-CEN5
NIP-Os4
Satellite:
1) NIP-Os6→Bst99-CEN07→Bd21-CEN1
2) NIP-Os5→Bst99-CEN08→Bd21-CEN2
3) NIP-Os10→Bst99-CEN03→Bd21-CEN3
4) NIP-Os11→Bst99-CEN05/CEN10→Bd21-CEN4
5) NIP-Os4→Bst99-CEN09→Bd21-CEN5
b
B. stacei
0.1
O. sativa
B. distachyon
CRM
1)
Bst99-CEN07
NIP-Os6
Bd21-CEN1
2)
Bd21-CEN2
NIP-Os5
Bst99-CEN08
3)
Bd21-CEN3
NIP-Os10
Bst99-CEN03
4)
Bst99-CEN10
Bst99-CEN05
NIP-Os6
5)
Bd21-CEN1
NIP-Os4
Bst99-CEN09
CRM:
1) NIP-Os6→Bst99-CEN07→Bd21-CEN1
2) NIP-Os5→Bst99-CEN08→Bd21-CEN2
3) NIP-Os10→Bst99-CEN03→Bd21-CEN3
4) NIP-Os11→Bst99-CEN05/CEN10→Bd21-CEN4
5) NIP-Os4→Bst99-CEN09→Bd21-CEN5

Fig. S13 Satellite repeats and retrotransposon elements library analyzed by syntenic centromeres between *O. sativa* and *Brachypodium* species.

Syntenic centromeres between chromosomes of *O. sativa* and *Brachypodium* species were listed in Fig. S10. Phylogenic trees were generated by neighbor-joining methods with FastTree tool. The pink, light blue, and purple indicate that the satellite repeats **(a)** or intact CRM retrotransposon elements **(b)** populations derived from *B. distachyon*, *B. stacei*, and *O. sativa*. The genetics tree on the left displays all the repeats from a species, and the dashed box on the right present detailed phylogenic trees between syntenic centromeres of *O. sativa* (NIP), *B. stacei* (Bst99) and *B. distachyon* (Bd21).

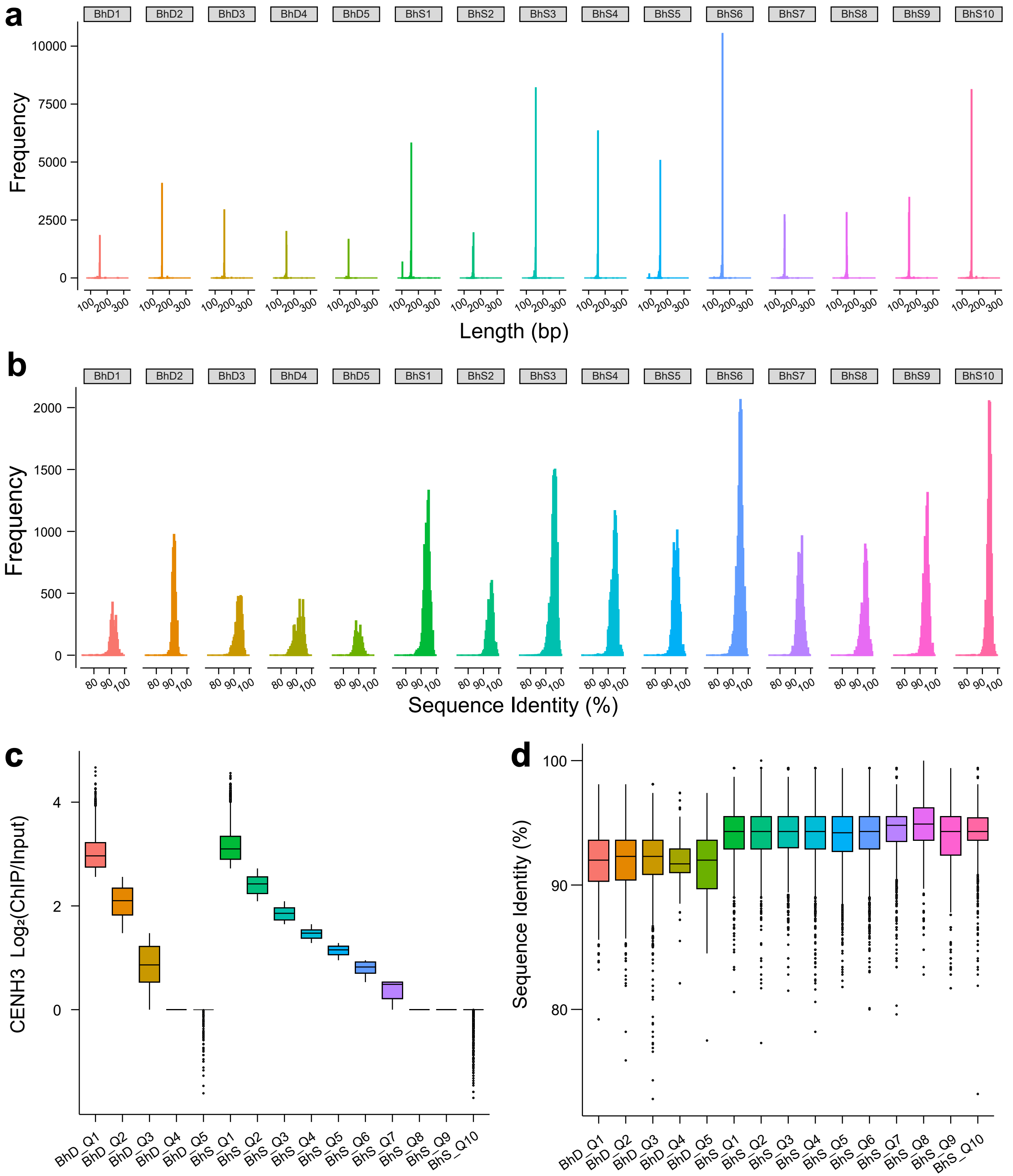
a
BhD1
BhD2
BhD3
BhD4
BhD5
BhS1
BhS2
BhS3
BhS4
BhS5
BhS6
BhS7
BhS8
BhS9
BhS10
Frequency
Length (bp)
b
Sequence Identity (%)
c
CENH3 Log₂(ChIP/Input)
BhD_Q1
BhD_Q2
BhD_Q3
BhD_Q4
BhD_Q5
BhS_Q1
BhS_Q2
BhS_Q3
BhS_Q4
BhS_Q5
BhS_Q6
BhS_Q7
BhS_Q8
BhS_Q9
BhS_Q10
d
Sequence Identity (%)

Fig. S14 The CentBd/CentBS satellite repeat library analyzed by chromosome in IBd483-CEN.

a-b Histograms of centromeric satellite monomer lengths (bp) **(a)** and sequences identity **(b)** relative to the genome-wide consensus, shown for each chromosome of IBd483-CEN genome. **c-d** Satellite density grouped by decreasing CENH3 $\log_2$(ChIP/Input) **(c)** and satellite density grouped by decreasing sequence identity **(d)** in IBd483-CEN. BhD subgenome was classified into five groups, and BhS subgenome was classified into ten groups.

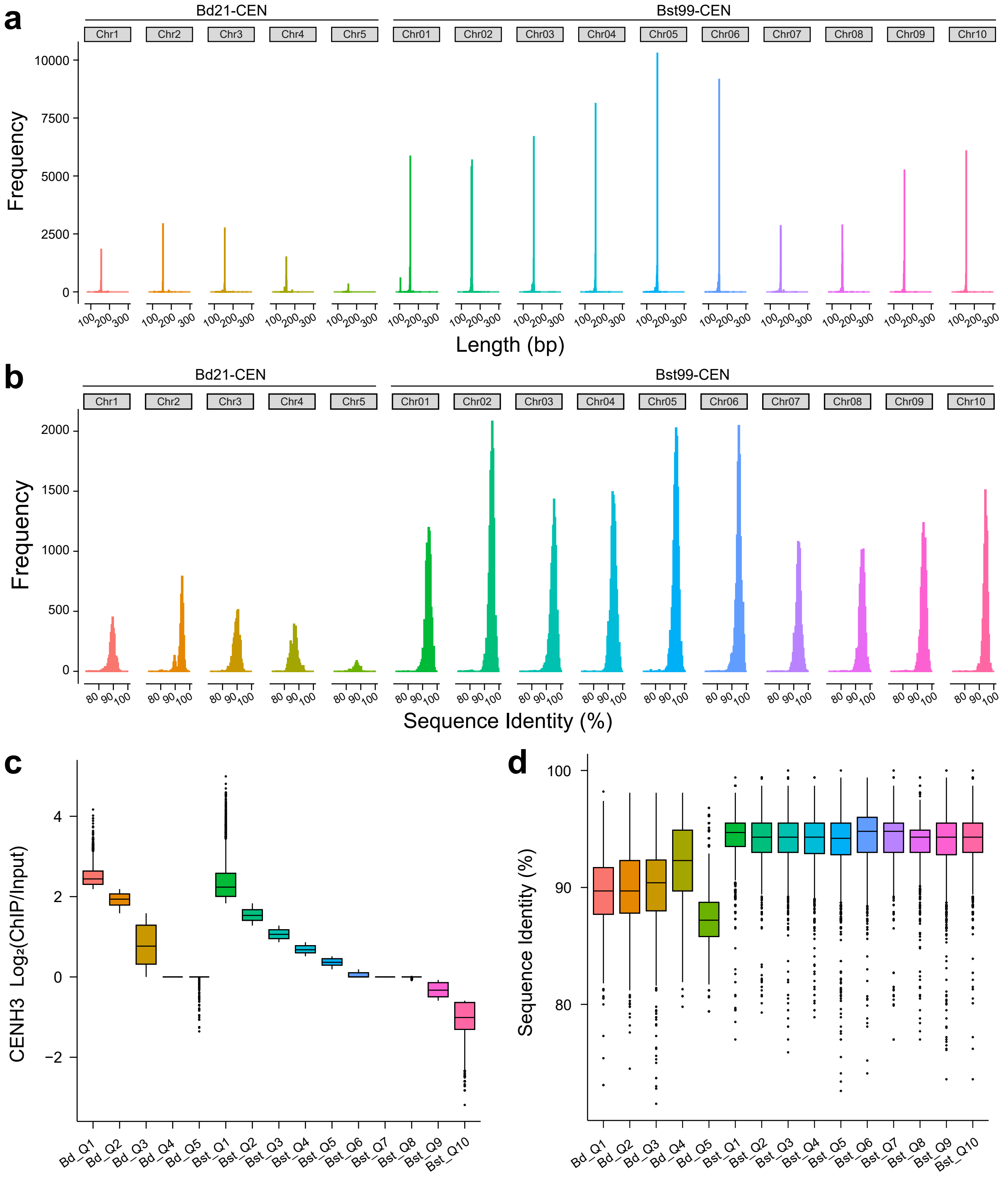
a
Bd21-CEN
Bst99-CEN
Chr1
Chr2
Chr3
Chr4
Chr5
Chr01
Chr02
Chr03
Chr04
Chr05
Chr06
Chr07
Chr08
Chr09
Chr10
Frequency
Length (bp)
b
Sequence Identity (%)
c
CENH3 Log₂(ChIP/Input)
Bd_Q1
Bd_Q2
Bd_Q3
Bd_Q4
Bd_Q5
Bst_Q1
Bst_Q2
Bst_Q3
Bst_Q4
Bst_Q5
Bst_Q6
Bst_Q7
Bst_Q8
Bst_Q9
Bst_Q10
d
Sequence Identity (%)

Fig. S15 The CentBd/CentBS satellite repeat libraries analyzed by chromosome in Bd21-CEN and Bst99-CEN.

a-b Histograms of CentBd or CentBs monomer lengths (bp) **(a)**, and sequence identities **(b)** relative to the genome-wide consensus, shown for each chromosome of Bd21-CEN and Bst99-CEN genome. **c-d** Satellite densities grouped by decreasing CENH3 $\log_2$(ChIP/Input) **(c)** and satellite density grouped by decreasing sequence identities **(d)** in Bd21-CEN and Bst99-CEN. Bd21-CEN genome was classified into five groups, and Bst99-CEN genome was classified into ten groups.

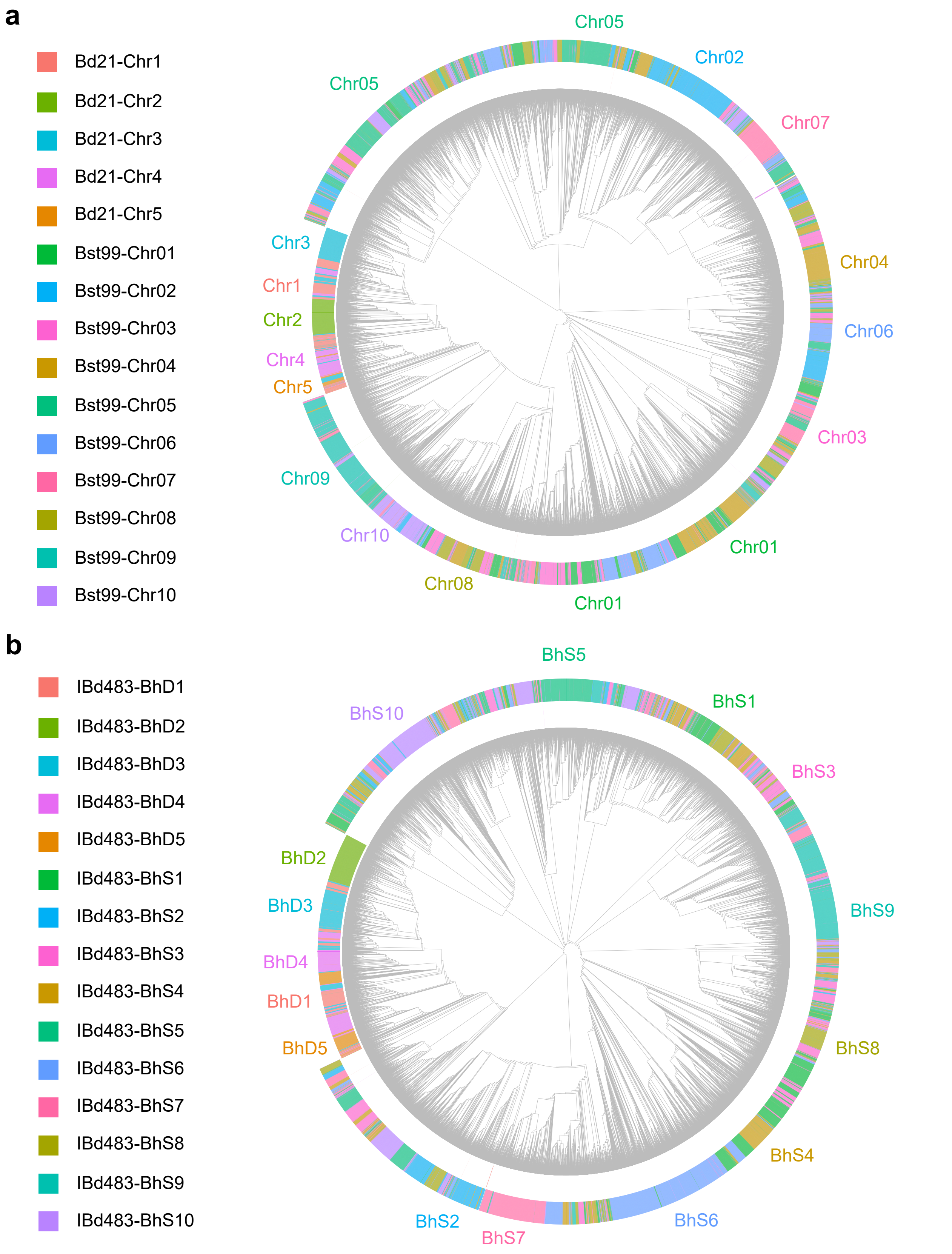

a
Bd21-Chr1
Bd21-Chr2
Bd21-Chr3
Bd21-Chr4
Bd21-Chr5
Bst99-Chr01
Bst99-Chr02
Bst99-Chr03
Bst99-Chr04
Bst99-Chr05
Bst99-Chr06
Bst99-Chr07
Bst99-Chr08
Bst99-Chr09
Bst99-Chr10
Chr05
Chr02
Chr07
Chr05
Chr04
Chr3
Chr1
Chr2
Chr06
Chr4
Chr5
Chr03
Chr09
Chr10
Chr01
Chr08
Chr01
b
IBd483-BhD1
IBd483-BhD2
IBd483-BhD3
IBd483-BhD4
IBd483-BhD5
IBd483-BhS1
IBd483-BhS2
IBd483-BhS3
IBd483-BhS4
IBd483-BhS5
IBd483-BhS6
IBd483-BhS7
IBd483-BhS8
IBd483-BhS9
IBd483-BhS10
BhS5
BhS10
BhS1
BhS3
BhD2
BhD3
BhS9
BhD4
BhD1
BhD5
BhS8
BhS4
BhS2
BhS7
BhS6

Fig. S16 The phylogenetic tree of CentBd or CentBs repeats in Bd21-CEN, Bst99-CEN and IBd483-CEN genome.

a-b Phylogenic comparisons were made between the genome of Bd21-CEN and Bst99-CEN **(a)** or the subgenomes of IBd483-CEN **(b)**. The phylogenic tree was generated by neighbor-joining methods. Strips outside the tree are colored by different chromosomes in each genome, which indicate the satellite repeats derived from specific chromosomes.

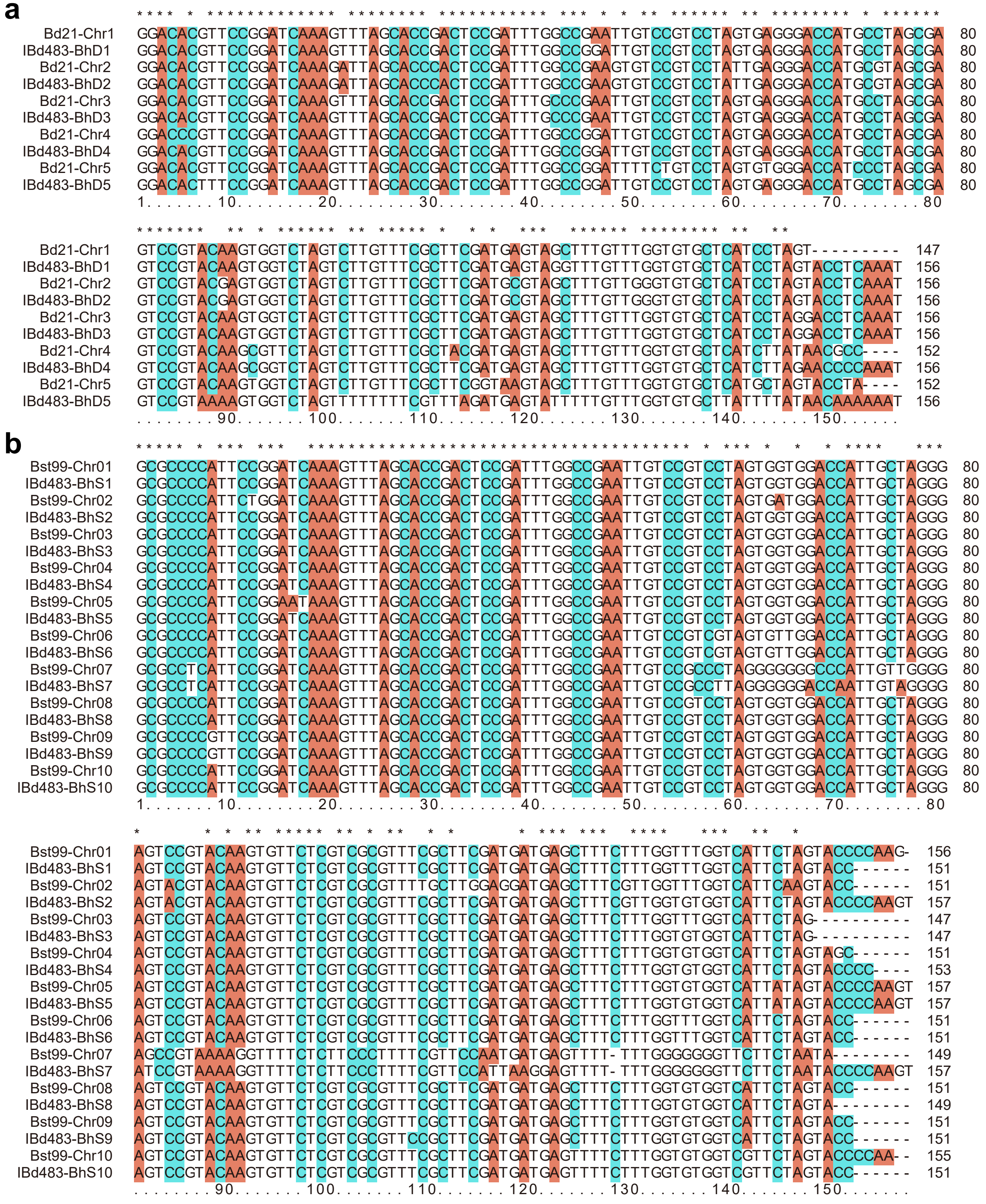
a
Bd21-Chr1
IBd483-BhD1
Bd21-Chr2
IBd483-BhD2
Bd21-Chr3
IBd483-BhD3
Bd21-Chr4
IBd483-BhD4
Bd21-Chr5
IBd483-BhD5
b
Bst99-Chr01
IBd483-BhS1
Bst99-Chr02
IBd483-BhS2
Bst99-Chr03
IBd483-BhS3
Bst99-Chr04
IBd483-BhS4
Bst99-Chr05
IBd483-BhS5
Bst99-Chr06
IBd483-BhS6
Bst99-Chr07
IBd483-BhS7
Bst99-Chr08
IBd483-BhS8
Bst99-Chr09
IBd483-BhS9
Bst99-Chr10
IBd483-BhS10

Fig. S17 Sequence alignment of representative CentBd (a) or CentBs (b) satellite monomers identified in each chromosome derived from IBd483-CEN, Bd21-CEN and Bst99-CEN genomes.

CentBd and CentBs satellite monomers with lengths of 146-160 bp were retained, and the pairwise sequence identity of satellite monomers was used to generate consensus sequences for each chromosome.

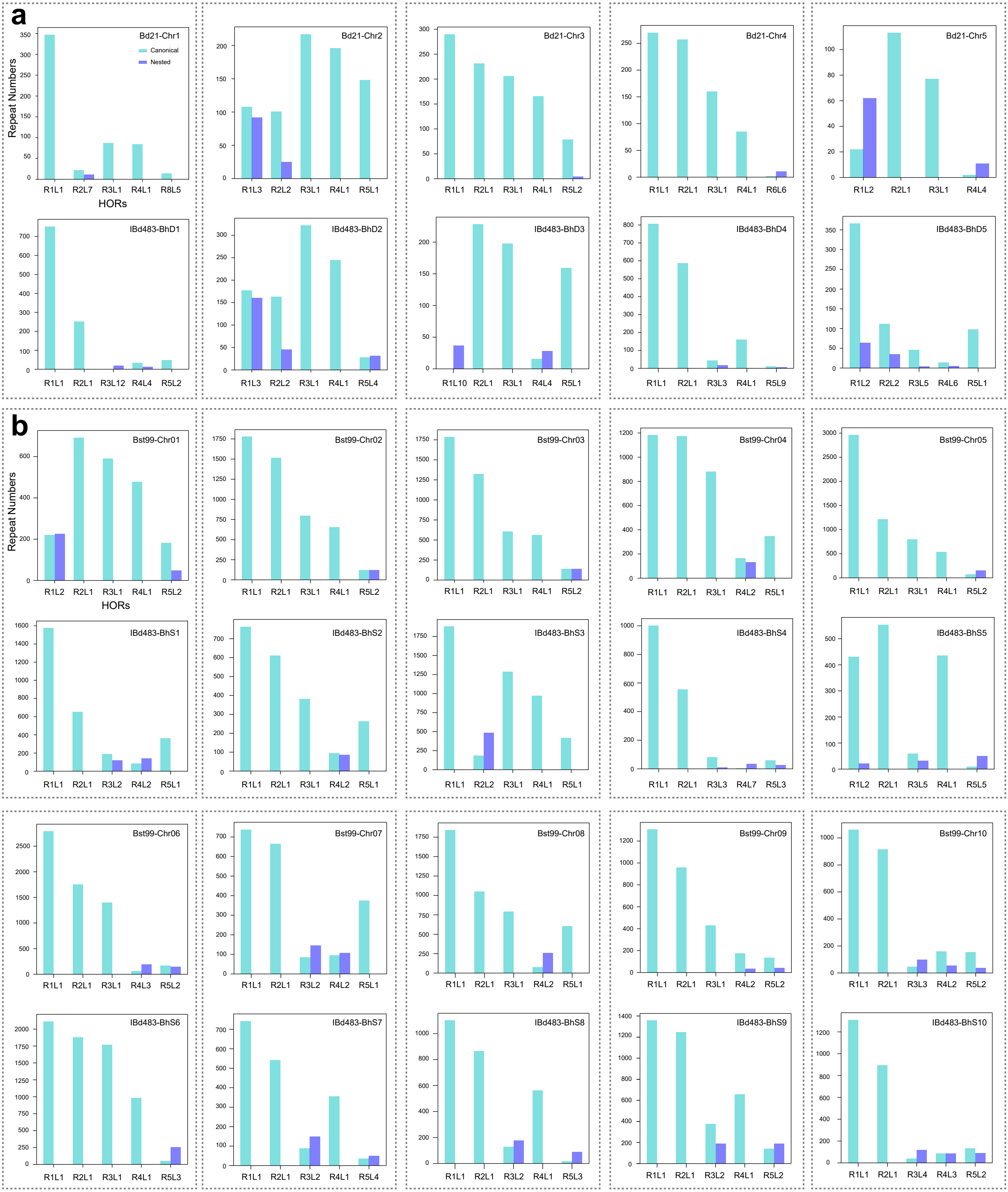
a
Repeat Numbers
HORs
Canonical
Nested
Bd21-Chr1
R1L1 R2L7 R3L1 R4L1 R8L5
Bd21-Chr2
R1L3 R2L2 R3L1 R4L1 R5L1
Bd21-Chr3
R1L1 R2L1 R3L1 R4L1 R5L2
Bd21-Chr4
R1L1 R2L1 R3L1 R4L1 R6L6
Bd21-Chr5
R1L2 R2L1 R3L1 R4L4
IBd483-BhD1
R1L1 R2L1 R3L12 R4L4 R5L2
IBd483-BhD2
R1L3 R2L2 R3L1 R4L1 R5L4
IBd483-BhD3
R1L10 R2L1 R3L1 R4L4 R5L1
IBd483-BhD4
R1L1 R2L1 R3L3 R4L1 R5L9
IBd483-BhD5
R1L2 R2L2 R3L5 R4L6 R5L1
b
Repeat Numbers
HORs
Bst99-Chr01
R1L2 R2L1 R3L1 R4L1 R5L2
Bst99-Chr02
R1L1 R2L1 R3L1 R4L1 R5L2
Bst99-Chr03
R1L1 R2L1 R3L1 R4L1 R5L2
Bst99-Chr04
R1L1 R2L1 R3L1 R4L2 R5L1
Bst99-Chr05
R1L1 R2L1 R3L1 R4L1 R5L2
IBd483-BhS1
R1L1 R2L1 R3L2 R4L2 R5L1
IBd483-BhS2
R1L1 R2L1 R3L1 R4L2 R5L1
IBd483-BhS3
R1L1 R2L2 R3L1 R4L1 R5L1
IBd483-BhS4
R1L1 R2L1 R3L3 R4L7 R5L3
IBd483-BhS5
R1L2 R2L1 R3L5 R4L1 R5L5
Bst99-Chr06
R1L1 R2L1 R3L1 R4L3 R5L2
Bst99-Chr07
R1L1 R2L1 R3L2 R4L2 R5L1
Bst99-Chr08
R1L1 R2L1 R3L1 R4L2 R5L1
Bst99-Chr09
R1L1 R2L1 R3L1 R4L2 R5L2
Bst99-Chr10
R1L1 R2L1 R3L3 R4L2 R5L2
IBd483-BhS6
R1L1 R2L1 R3L1 R4L1 R5L3
IBd483-BhS7
R1L1 R2L1 R3L2 R4L1 R5L4
IBd483-BhS8
R1L1 R2L1 R3L2 R4L1 R5L3
IBd483-BhS9
R1L1 R2L1 R3L2 R4L1 R5L2
IBd483-BhS10
R1L1 R2L1 R3L4 R4L3 R5L2

Fig. S18 The HOR repeat number in each chromosome of IBd483-CEN, Bd21-CEN and Bst99-CEN.

a The numbers of top five frequent HOR repeats in Bd21-CEN and BhD subgenome of IBd483-CEN. Specifically, Chr5 in Bd21-CEN genome has only four frequent HORs.
b The numbers of top five frequent HOR repeats in Bst99-CEN and BhS subgenome of IBd483-CEN. Here, "Canonical" represents canonical HORs, "Nested" represents nested HORs.

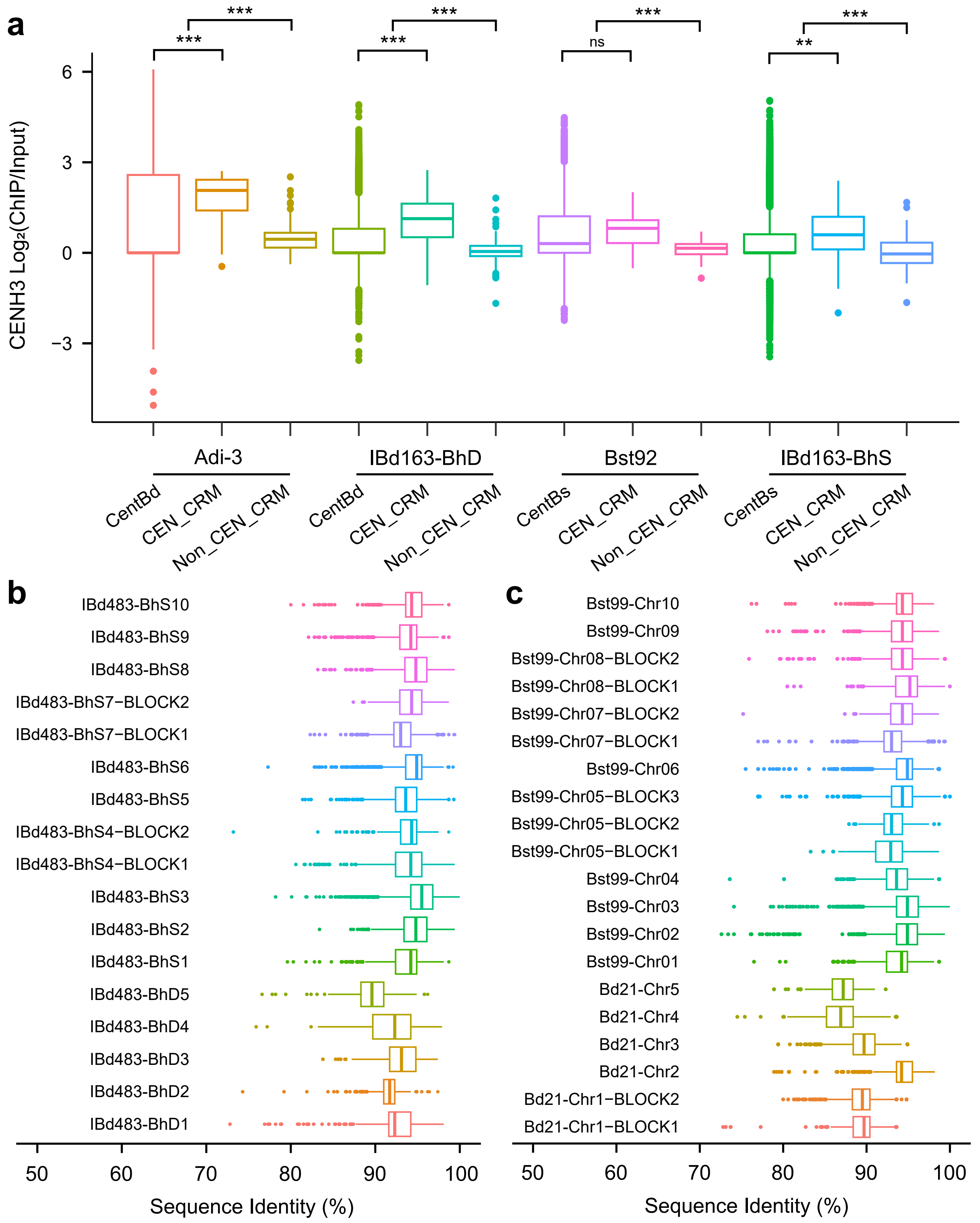
a
CENH3 Log₂(ChIP/Input)
***
***
ns
**
Adi-3
IBd163-BhD
Bst92
IBd163-BhS
CentBd
CEN_CRM
Non_CEN_CRM
CentBs
b
IBd483-BhS10
IBd483-BhS9
IBd483-BhS8
IBd483-BhS7−BLOCK2
IBd483-BhS7−BLOCK1
IBd483-BhS6
IBd483-BhS5
IBd483-BhS4−BLOCK2
IBd483-BhS4−BLOCK1
IBd483-BhS3
IBd483-BhS2
IBd483-BhS1
IBd483-BhD5
IBd483-BhD4
IBd483-BhD3
IBd483-BhD2
IBd483-BhD1
Sequence Identity (%)
c
Bst99-Chr10
Bst99-Chr09
Bst99-Chr08−BLOCK2
Bst99-Chr08−BLOCK1
Bst99-Chr07−BLOCK2
Bst99-Chr07−BLOCK1
Bst99-Chr06
Bst99-Chr05−BLOCK3
Bst99-Chr05−BLOCK2
Bst99-Chr05−BLOCK1
Bst99-Chr04
Bst99-Chr03
Bst99-Chr02
Bst99-Chr01
Bd21-Chr5
Bd21-Chr4
Bd21-Chr3
Bd21-Chr2
Bd21-Chr1−BLOCK2
Bd21-Chr1−BLOCK1
Sequence Identity (%)

Fig. S19 Characterization of centromere repeat arrays in *Brachypodium* genus.

a CENH3 enrichment level (log_2(ChIP/Input)) around CentBd/CentBs satellite, centromeric intact CRM and non-centromeric intact CRM elements in IBd163, Adi-3, and Bst92 lines. T-test, $*P < 0.05$, $**P < 0.01$, $***P < 0.001$. **b-c** Distribution of satellite sequence identity relative to the genome-wide consensus, shown for each chromosome or chromosome block in IBd483 **(b)** or Bd21-CEN and Bst99-CEN **(c)**. Block information of satellite repeat in IBd483-CEN, Bst99-CEN and Bd21-CEN centromeres can be found in Additional file 2: Table S15.

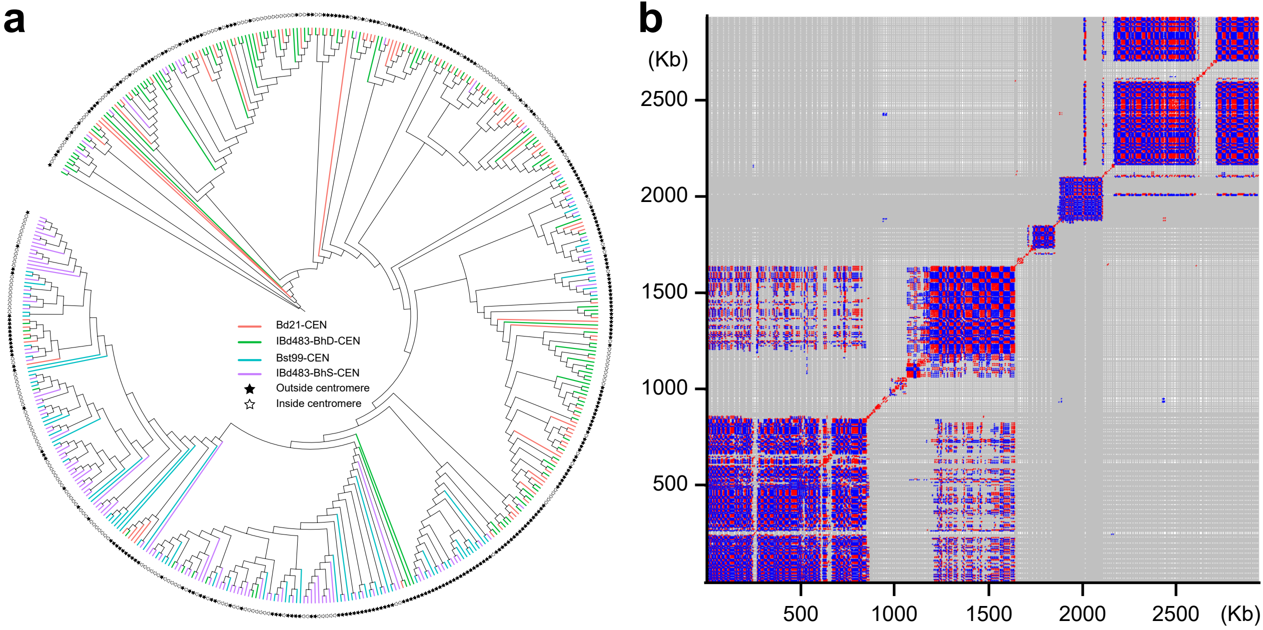
a
Bd21-CEN
IBd483-BhD-CEN
Bst99-CEN
IBd483-BhS-CEN
Outside centromere
Inside centromere
b
(Kb)
2500
2000
1500
1000
500
500
1000
1500
2000
2500
(Kb)

Fig. S20 Characterization of CRM retrotransposons in *Brachypodium* centromeres.

a Phylogenetic tree of intact CRM elements from Bd21-CEN (light red), Bst99-CEN (cyan), BhD (green) and BhS (purple) subgenome of IBd483-CEN assemblies. Stars at the branch tips indicate CRM inside (white) or outside (black) the centromeres. **b** Dot plot of centromeric CRM using a 156-bp search window. Red and blue indicate forward- and reverse-strand similarity, respectively.

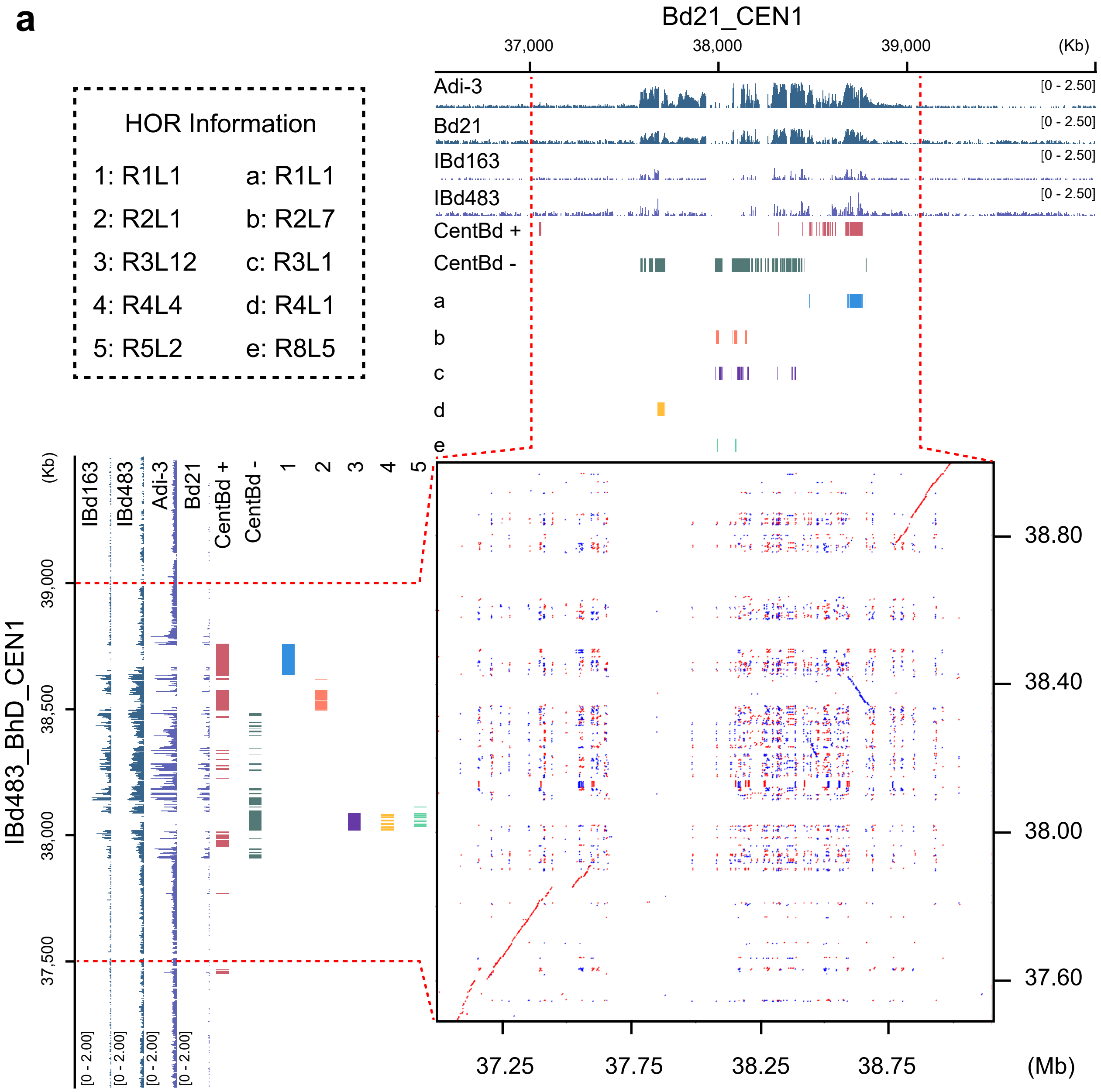
a
Bd21_CEN1
37,000
38,000
39,000
(Kb)
Adi-3
Bd21
IBd163
IBd483
CentBd +
CentBd -
a
b
c
d
e
[0 - 2.50]
HOR Information
1: R1L1
2: R2L1
3: R3L12
4: R4L4
5: R5L2
a: R1L1
b: R2L7
c: R3L1
d: R4L1
e: R8L5
IBd483_BhD_CEN1
(Kb)
39,000
38,500
38,000
37,500
IBd163
IBd483
Adi-3
Bd21
CentBd +
CentBd -
1
2
3
4
5
[0 - 2.00]
38.80
38.40
38.00
37.60
37.25
37.75
38.25
38.75
(Mb)

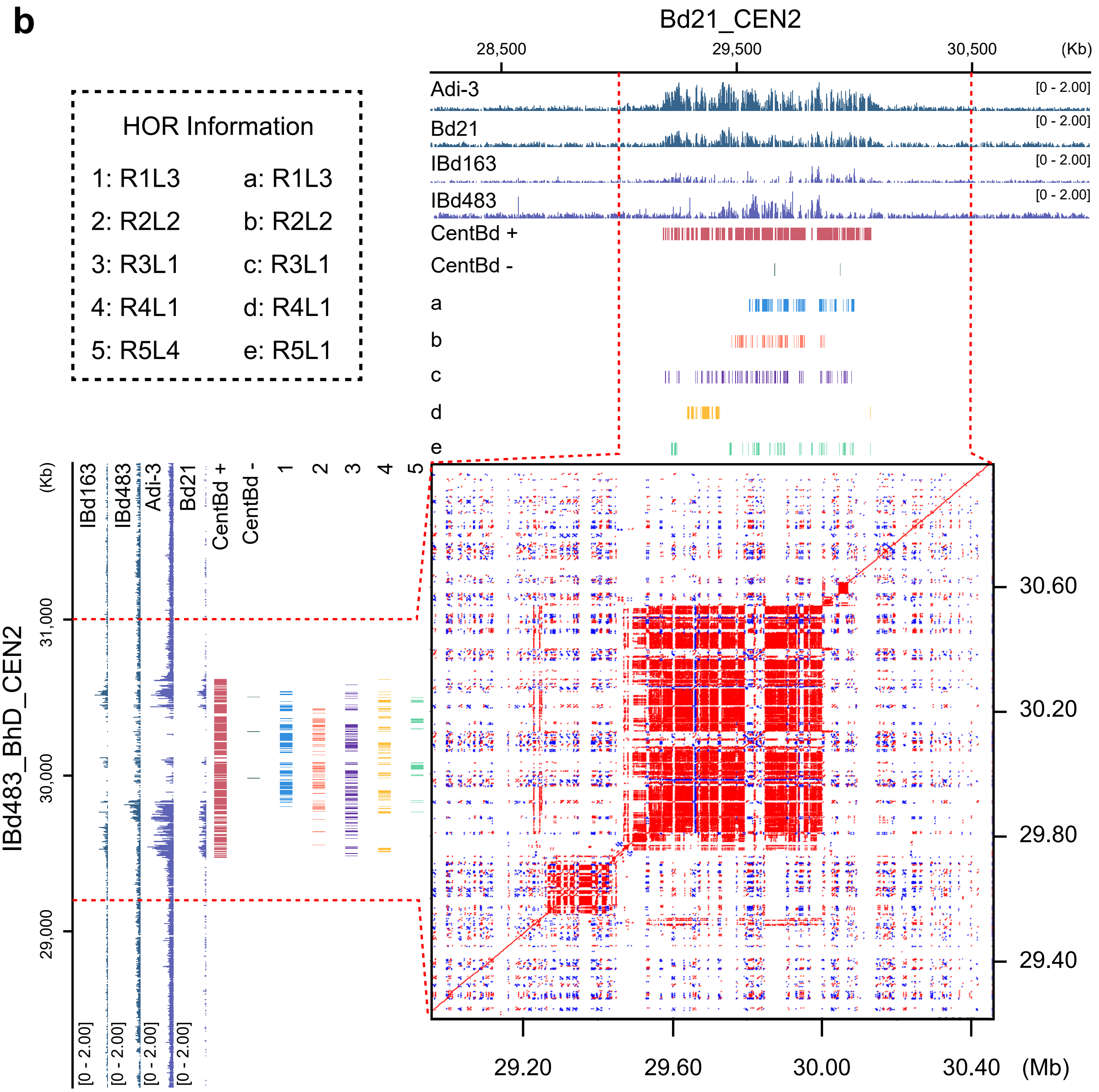

b
Bd21_CEN2
28,500
29,500
30,500
(Kb)
Adi-3
Bd21
IBd163
IBd483
[0 - 2.00]
CentBd +
CentBd -
a
b
c
d
e
HOR Information
1: R1L3
2: R2L2
3: R3L1
4: R4L1
5: R5L4
a: R1L3
b: R2L2
c: R3L1
d: R4L1
e: R5L1
IBd483_BhD_CEN2
(Kb)
31,000
30,000
29,000
1
2
3
4
5
30.60
30.20
29.80
29.40
29.20
29.60
30.00
30.40
(Mb)

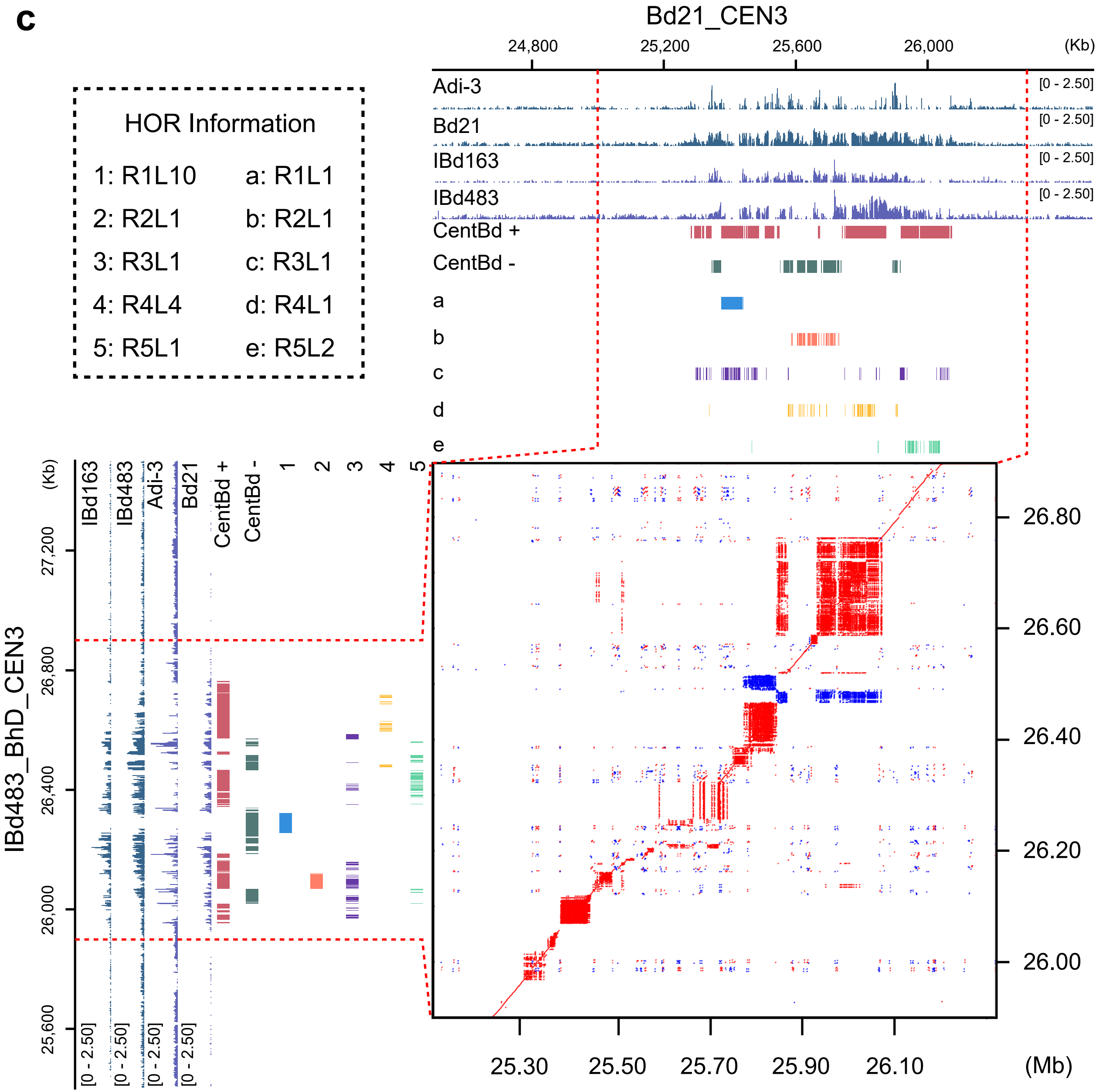
c
Bd21_CEN3
24,800
25,200
25,600
26,000
(Kb)
Adi-3
Bd21
IBd163
IBd483
[0 - 2.50]
CentBd +
CentBd -
a
b
c
d
e
HOR Information
1: R1L10
2: R2L1
3: R3L1
4: R4L4
5: R5L1
a: R1L1
b: R2L1
c: R3L1
d: R4L1
e: R5L2
IBd483_BhD_CEN3
(Kb)
27,200
26,800
26,400
26,000
25,600
1
2
3
4
5
26.80
26.60
26.40
26.20
26.00
25.30
25.50
25.70
25.90
26.10
(Mb)

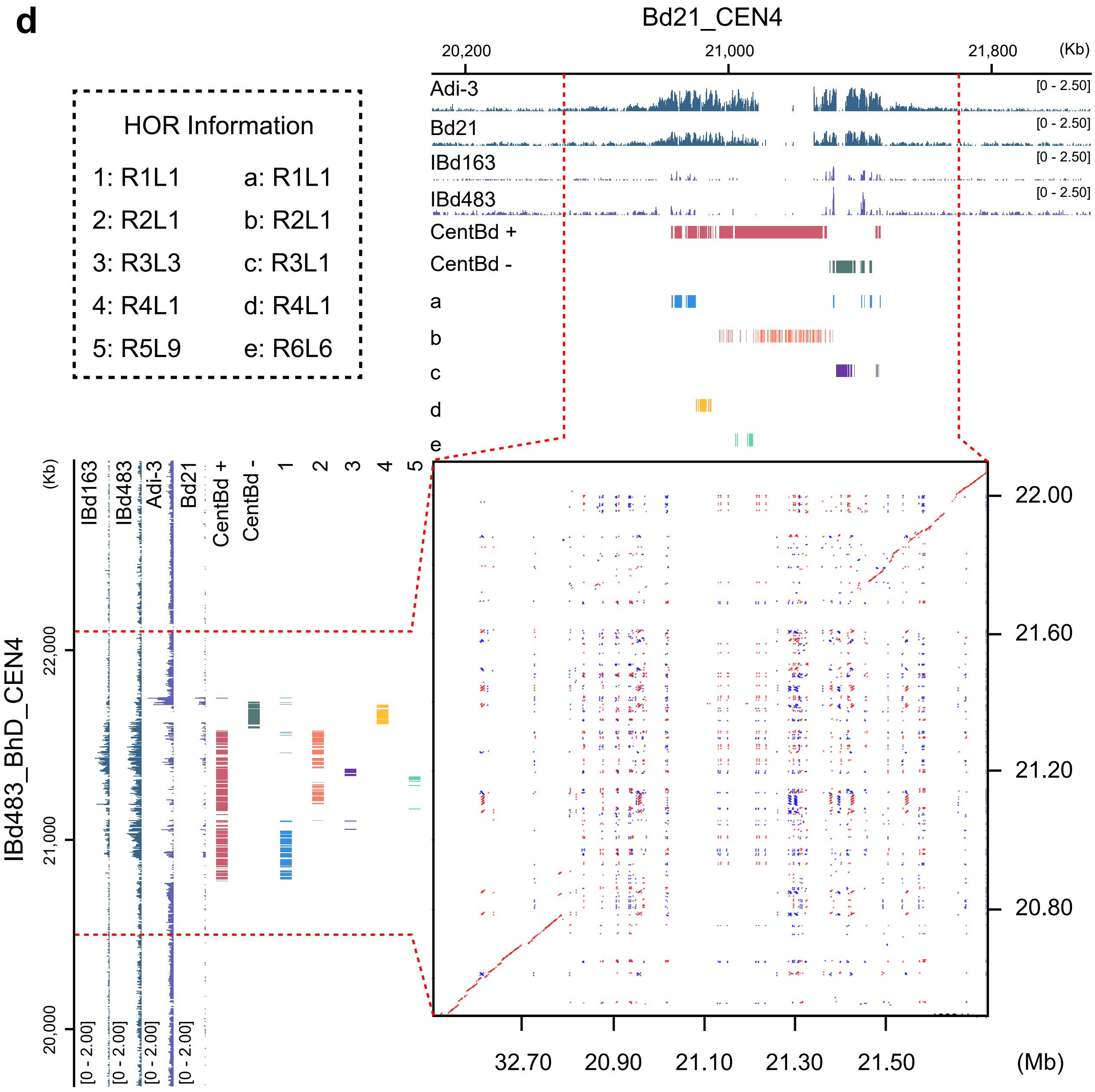

d
Bd21_CEN4
20,200
21,000
21,800
(Kb)
Adi-3
Bd21
IBd163
IBd483
CentBd +
CentBd -
a
b
c
d
e
[0 - 2.50]
HOR Information
1: R1L1
2: R2L1
3: R3L3
4: R4L1
5: R5L9
a: R1L1
b: R2L1
c: R3L1
d: R4L1
e: R6L6
IBd483_BhD_CEN4
(Kb)
22,000
21,000
20,000
IBd163
IBd483
Adi-3
Bd21
CentBd +
CentBd -
1
2
3
4
5
[0 - 2.00]
22.00
21.60
21.20
20.80
32.70
20.90
21.10
21.30
21.50
(Mb)

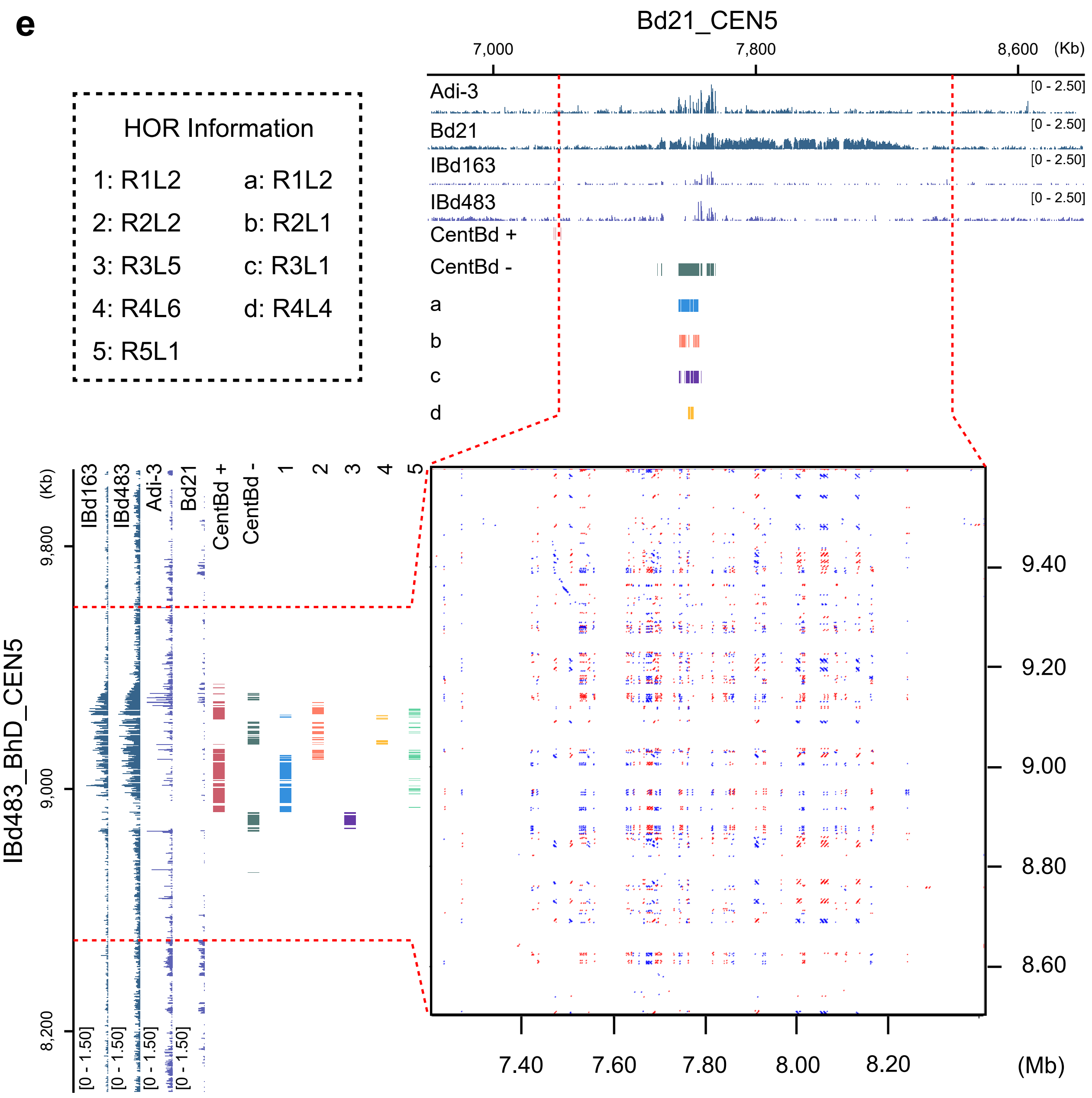

e
Bd21_CEN5
7,000
7,800
8,600
(Kb)
Adi-3
Bd21
IBd163
IBd483
CentBd +
CentBd -
a
b
c
d
[0 - 2.50]
HOR Information
1: R1L2
2: R2L2
3: R3L5
4: R4L6
5: R5L1
a: R1L2
b: R2L1
c: R3L1
d: R4L4
IBd483_BhD_CEN5
(Kb)
9,800
9,000
8,200
IBd163
IBd483
Adi-3
Bd21
CentBd +
CentBd -
1
2
3
4
5
[0 - 1.50]
9.40
9.20
9.00
8.80
8.60
7.40
7.60
7.80
8.00
8.20
(Mb)

Fig. S21 Characterization of centromere sequence, structure and CENH3 enrichments between the homologous chromosome pairs from diploid to tetraploid in D sub/genome.

a Bd21-CEN1 vs. IBd483-BhD-CEN1. **b** Bd21-CEN2 vs. IBd483-BhD-CEN2. **c** Bd21-CEN3 vs. IBd483-BhD-CEN3. **d** Bd21-CEN4 vs. IBd483-BhD-CEN4. **e** Bd21-CEN5 vs. IBd483-BhD-CEN5. CENH3 ChIP-seq mapping coverage from Adi-3, Bd21, IBd163 and IBd483 to the Bd21-CEN genome with the coordinate at top. The mapping coverage from IBd163, IBd483, Adi-3 and Bd21 to the IBd483-CEN genome with coordinate at left side. The satellite monomer and HOR array annotated on different centromeres (Track 5–11). Dot plots comparing the homologous centromere pairs between diploid to tetraploid using a search window of 156-bp. Red and blue indicate forward- and reverse-strand similarity, respectively.

a

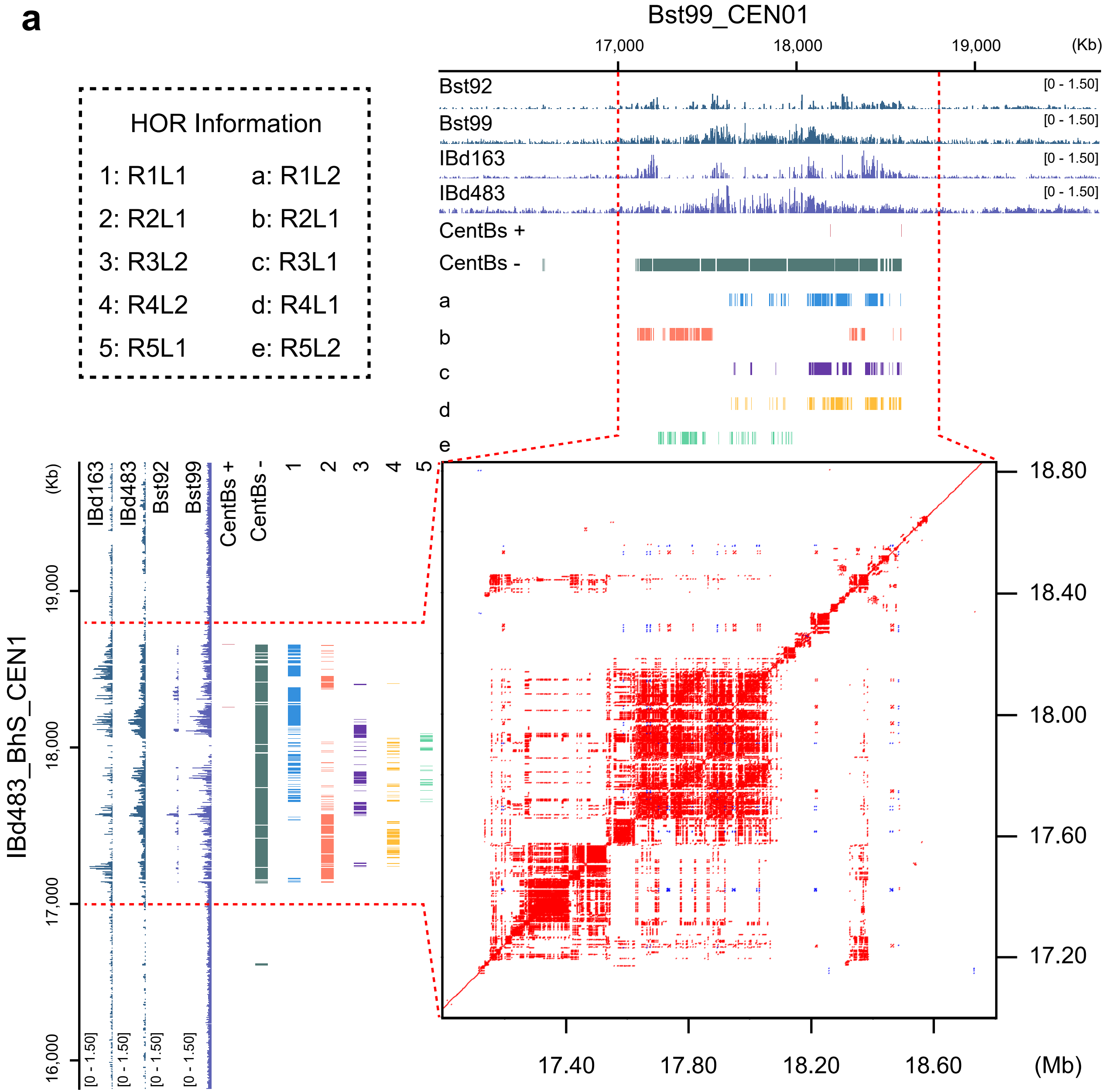

Bst99_CEN01
17,000
18,000
19,000
(Kb)
Bst92
Bst99
IBd163
IBd483
[0 - 1.50]
CentBs +
CentBs -
a
b
c
d
e
HOR Information
1: R1L1
a: R1L2
2: R2L1
b: R2L1
3: R3L2
c: R3L1
4: R4L2
d: R4L1
5: R5L1
e: R5L2
IBd483_BhS_CEN1
16,000
17,000
18,000
19,000
(Kb)
IBd163
IBd483
Bst92
Bst99
CentBs +
CentBs -
1
2
3
4
5
18.80
18.40
18.00
17.60
17.20
17.40
17.80
18.20
18.60
(Mb)

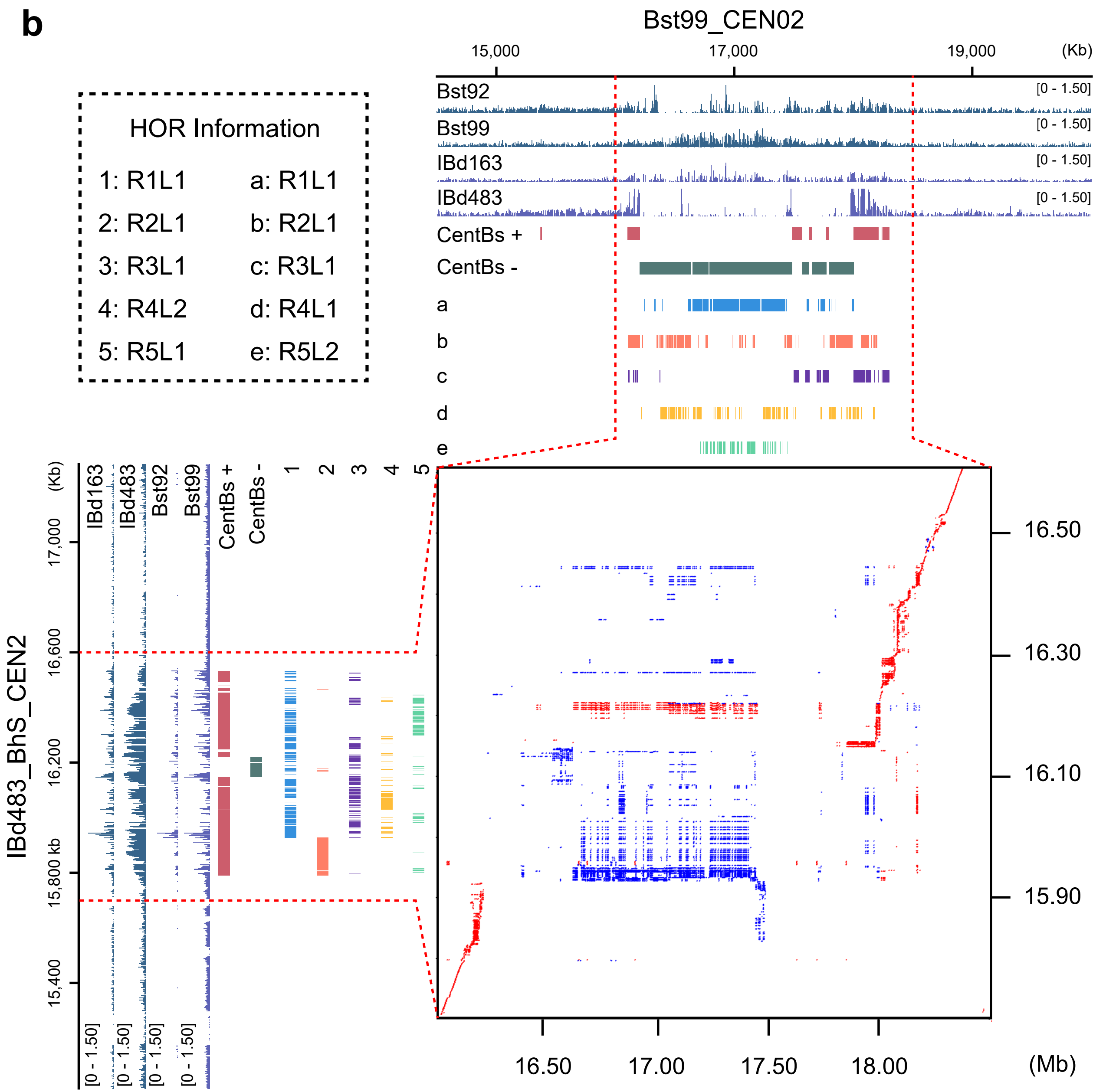
b
Bst99_CEN02
15,000
17,000
19,000
(Kb)
Bst92
Bst99
IBd163
IBd483
[0 - 1.50]
CentBs +
CentBs -
a
b
c
d
e
HOR Information
1: R1L1
a: R1L1
2: R2L1
b: R2L1
3: R3L1
c: R3L1
4: R4L2
d: R4L1
5: R5L1
e: R5L2
IBd483_BhS_CEN2
17,000
16,600
16,200
15,800 kb
15,400
(Kb)
IBd163
IBd483
Bst92
Bst99
CentBs +
CentBs -
1
2
3
4
5
16.50
16.30
16.10
15.90
16.50
17.00
17.50
18.00
(Mb)

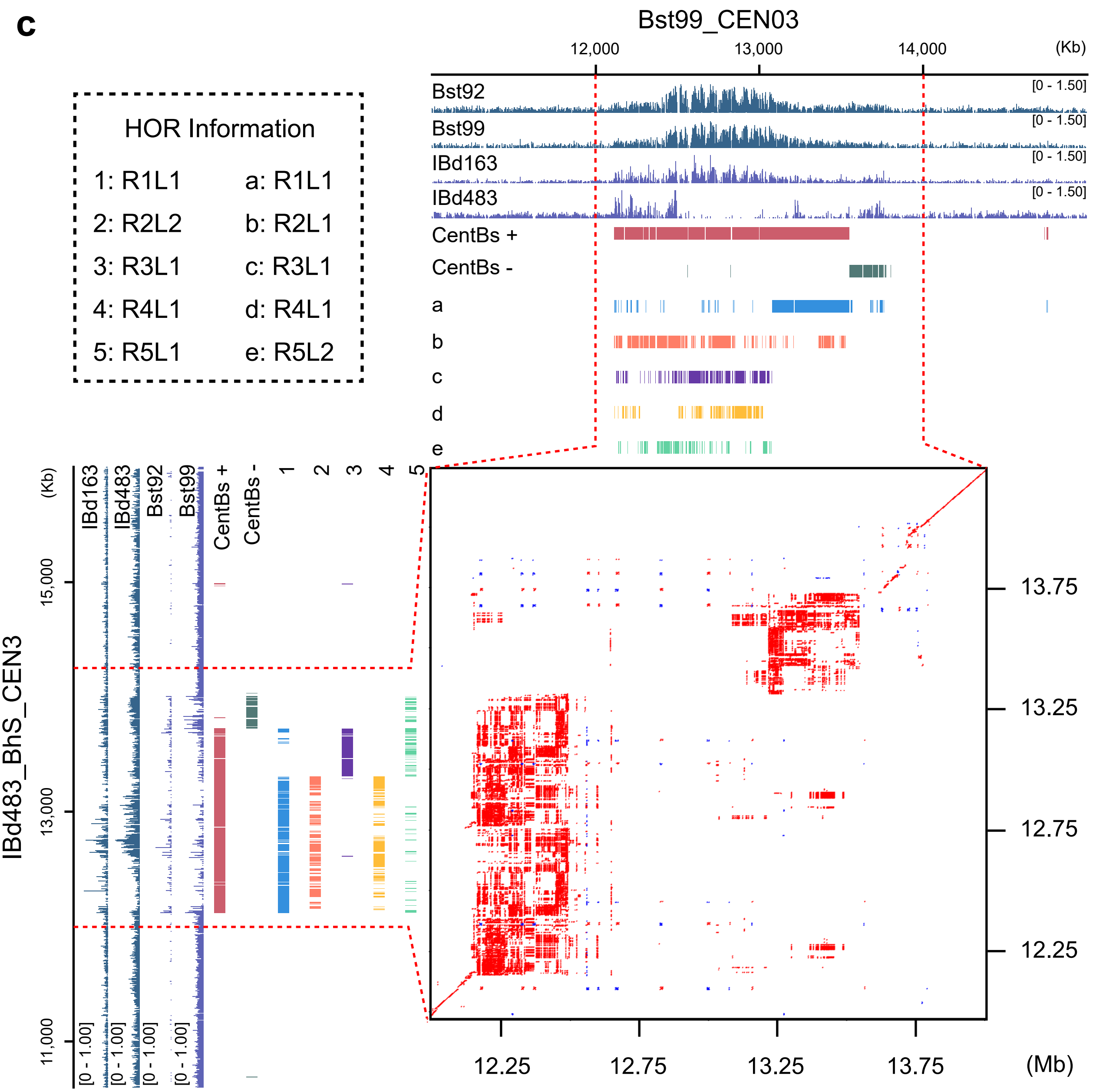
c
Bst99_CEN03
12,000
13,000
14,000
(Kb)
HOR Information
1: R1L1
2: R2L2
3: R3L1
4: R4L1
5: R5L1
a: R1L1
b: R2L1
c: R3L1
d: R4L1
e: R5L2
Bst92
Bst99
IBd163
IBd483
[0 - 1.50]
CentBs +
CentBs -
a
b
c
d
e
IBd483_BhS_CEN3
(Kb)
15,000
13,000
11,000
IBd163
IBd483
Bst92
Bst99
CentBs +
CentBs -
1
2
3
4
5
[0 - 1.00]
13.75
13.25
12.75
12.25
(Mb)

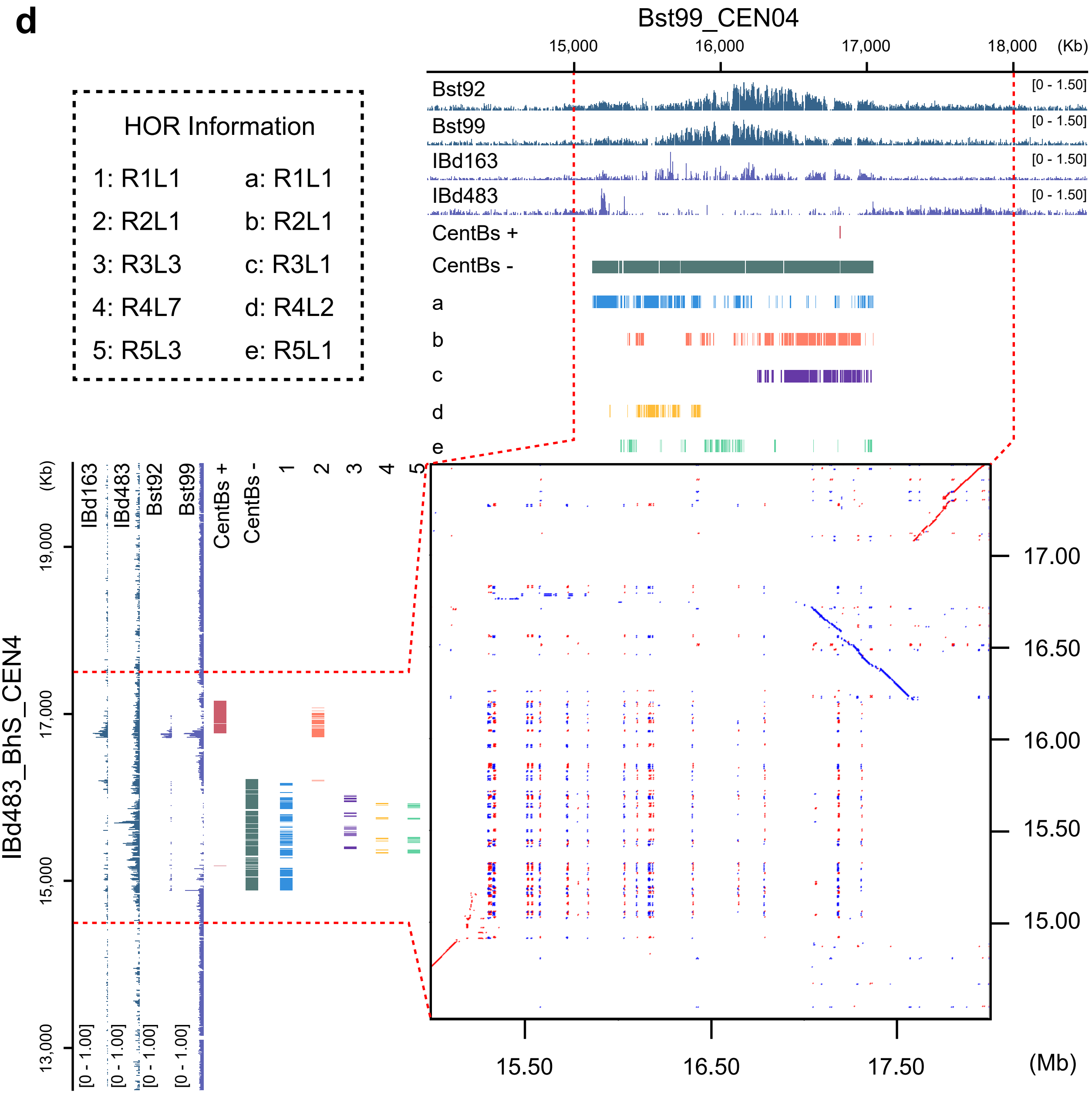
d
Bst99_CEN04
15,000
16,000
17,000
18,000
(Kb)
Bst92
Bst99
IBd163
IBd483
[0 - 1.50]
CentBs +
CentBs -
a
b
c
d
e
HOR Information
1: R1L1
2: R2L1
3: R3L3
4: R4L7
5: R5L3
a: R1L1
b: R2L1
c: R3L1
d: R4L2
e: R5L1
IBd483_BhS_CEN4
(Kb)
19,000
17,000
15,000
13,000
IBd163
IBd483
Bst92
Bst99
CentBs +
CentBs -
1
2
3
4
5
[0 - 1.00]
17.00
16.50
16.00
15.50
15.00
15.50
16.50
17.50
(Mb)

e

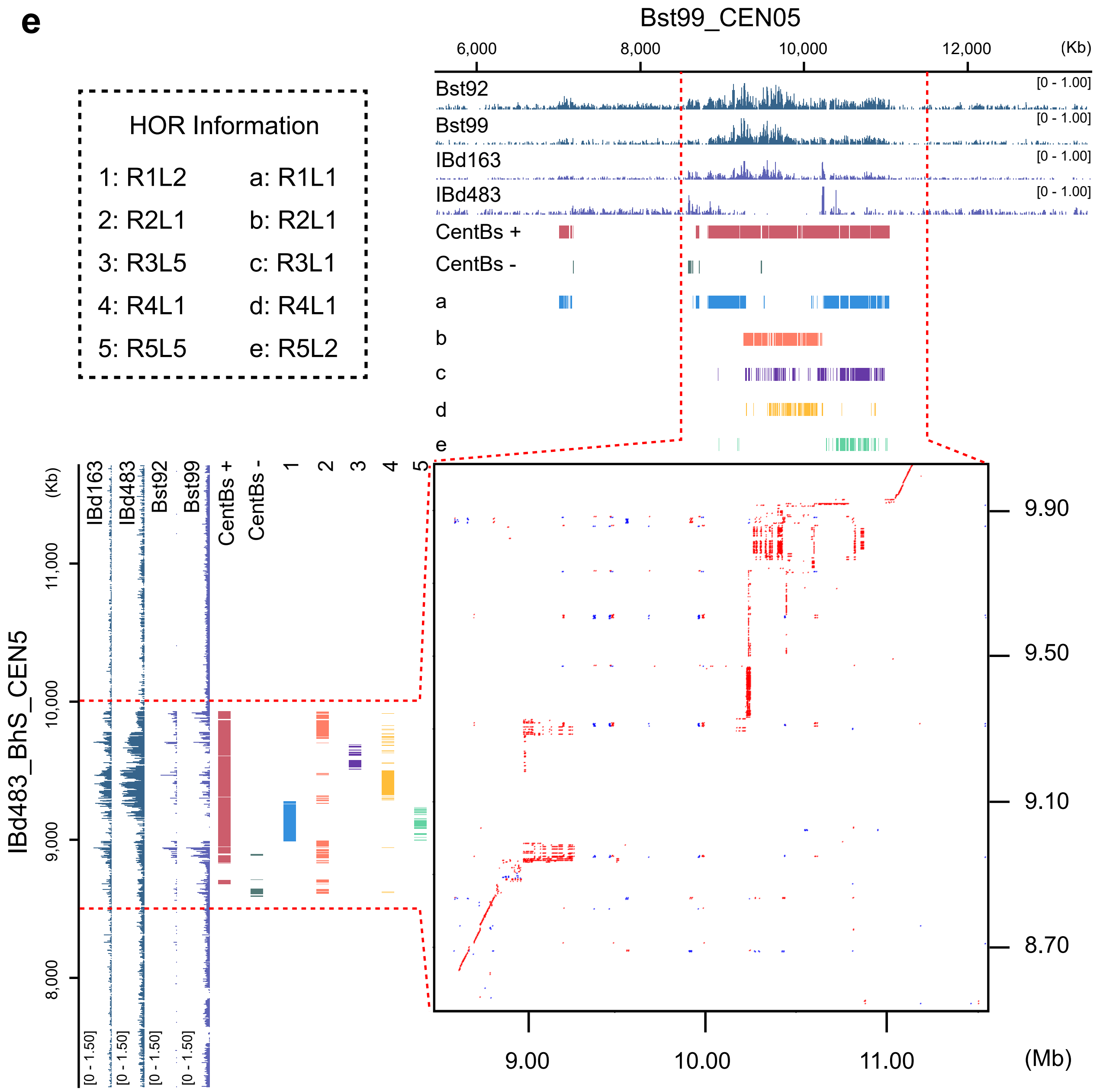

Bst99_CEN05
6,000
8,000
10,000
12,000
(Kb)
Bst92
Bst99
IBd163
IBd483
[0 - 1.00]
CentBs +
CentBs -
a
b
c
d
e
HOR Information
1: R1L2
2: R2L1
3: R3L5
4: R4L1
5: R5L5
a: R1L1
b: R2L1
c: R3L1
d: R4L1
e: R5L2
IBd483_BhS_CEN5
(Kb)
8,000
9,000
10,000
11,000
IBd163
IBd483
Bst92
Bst99
CentBs +
CentBs -
1
2
3
4
5
[0 - 1.50]
9.90
9.50
9.10
8.70
9.00
10.00
11.00
(Mb)

f

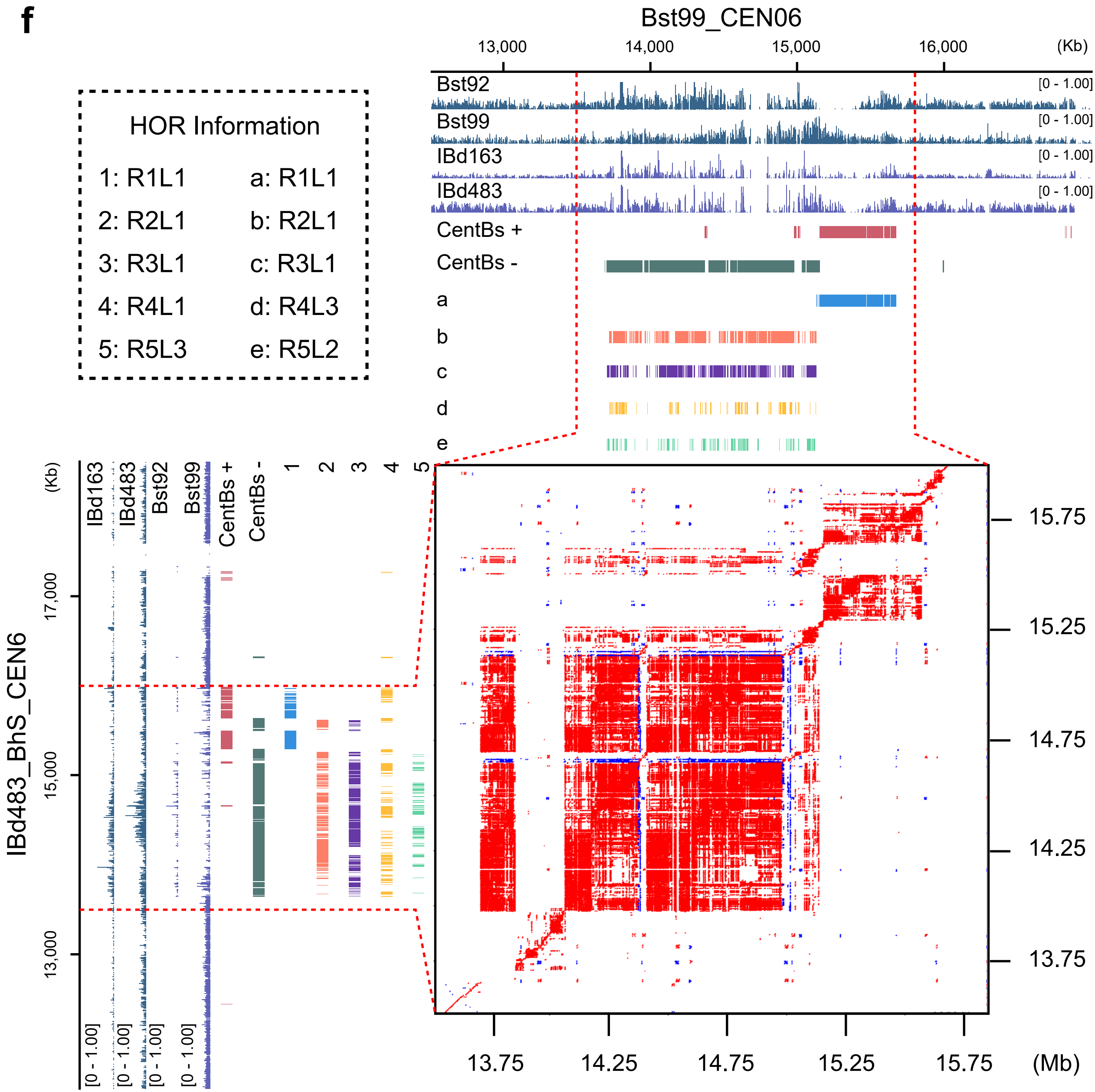
Bst99_CEN06
13,000
14,000
15,000
16,000
(Kb)
Bst92
Bst99
IBd163
IBd483
[0 - 1.00]
CentBs +
CentBs -
a
b
c
d
e
HOR Information
1: R1L1
a: R1L1
2: R2L1
b: R2L1
3: R3L1
c: R3L1
4: R4L1
d: R4L3
5: R5L3
e: R5L2
IBd483_BhS_CEN6
(Kb)
17,000
15,000
13,000
IBd163
IBd483
Bst92
Bst99
CentBs +
CentBs -
1
2
3
4
5
15.75
15.25
14.75
14.25
13.75
(Mb)

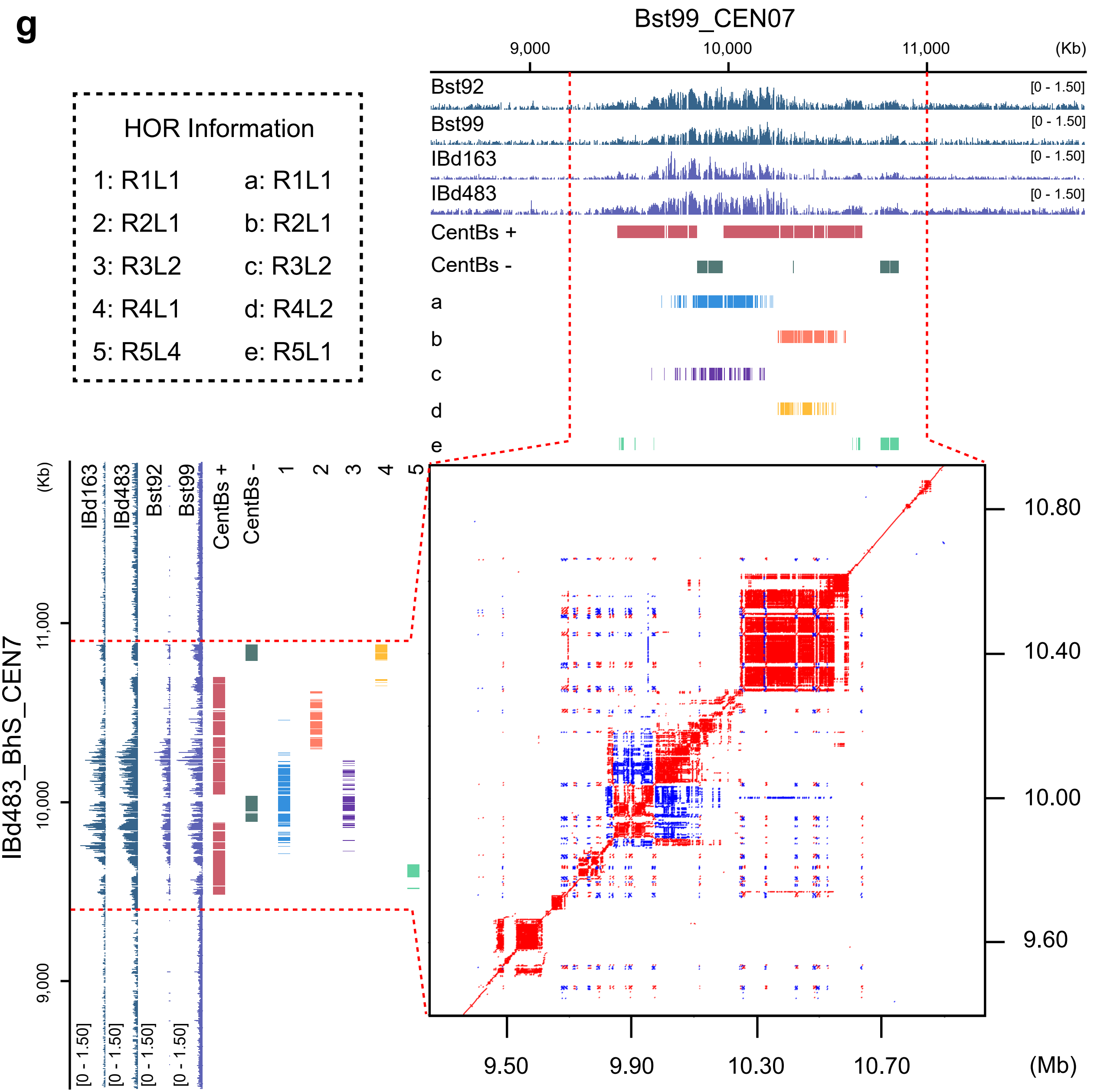

g
Bst99_CEN07
9,000
10,000
11,000
(Kb)
Bst92
Bst99
IBd163
IBd483
[0 - 1.50]
CentBs +
CentBs -
a
b
c
d
e
HOR Information
1: R1L1
2: R2L1
3: R3L2
4: R4L1
5: R5L4
a: R1L1
b: R2L1
c: R3L2
d: R4L2
e: R5L1
IBd483_BhS_CEN7
(Kb)
11,000
10,000
9,000
1
2
3
4
5
10.80
10.40
10.00
9.60
9.50
9.90
10.30
10.70
(Mb)

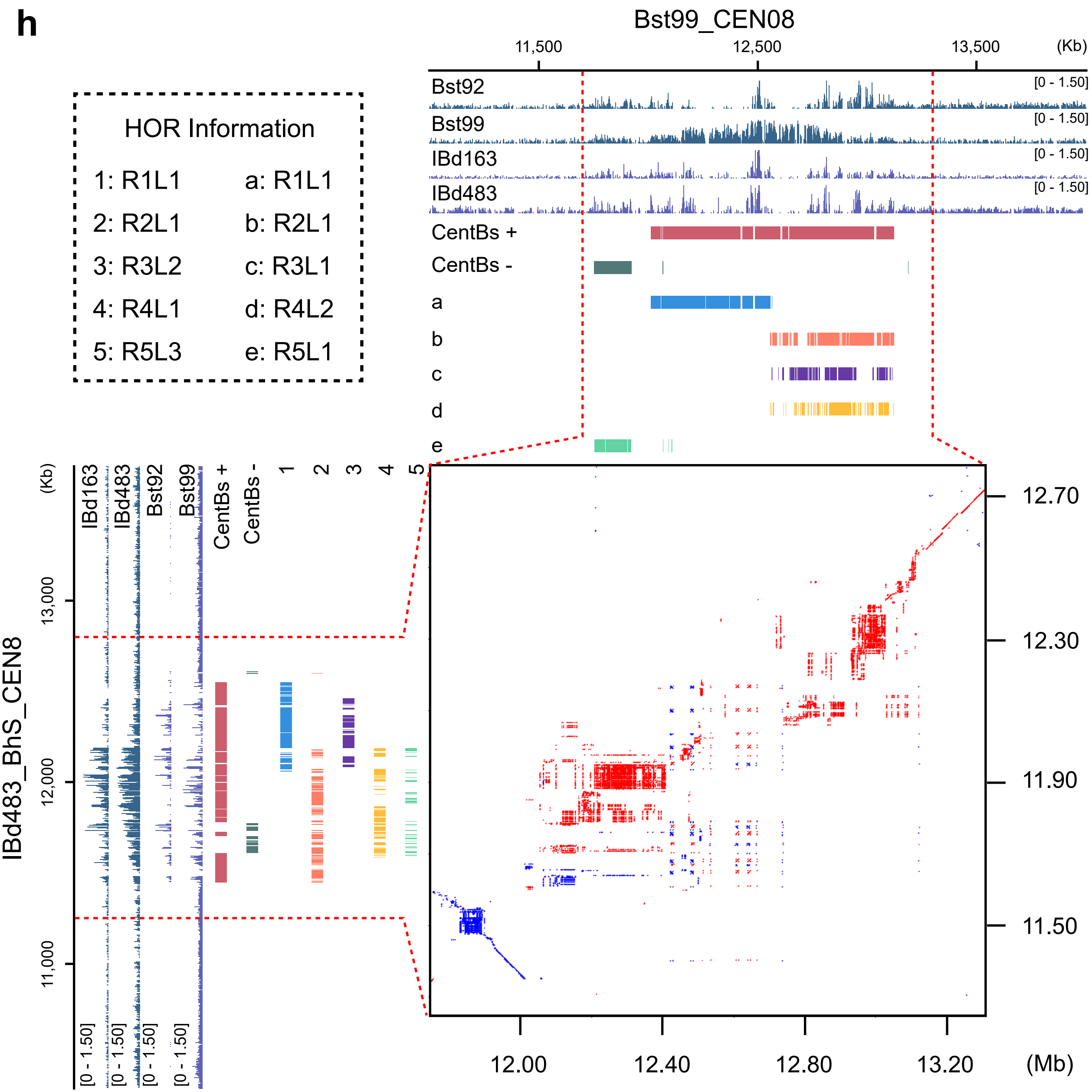

h
Bst99_CEN08
11,500
12,500
13,500
(Kb)
Bst92
Bst99
IBd163
IBd483
[0 - 1.50]
CentBs +
CentBs -
a
b
c
d
e
HOR Information
1: R1L1
a: R1L1
2: R2L1
b: R2L1
3: R3L2
c: R3L1
4: R4L1
d: R4L2
5: R5L3
e: R5L1
IBd483_BhS_CEN8
(Kb)
13,000
12,000
11,000
IBd163
IBd483
Bst92
Bst99
CentBs +
CentBs -
1
2
3
4
5
12.70
12.30
11.90
11.50
12.00
12.40
12.80
13.20
(Mb)

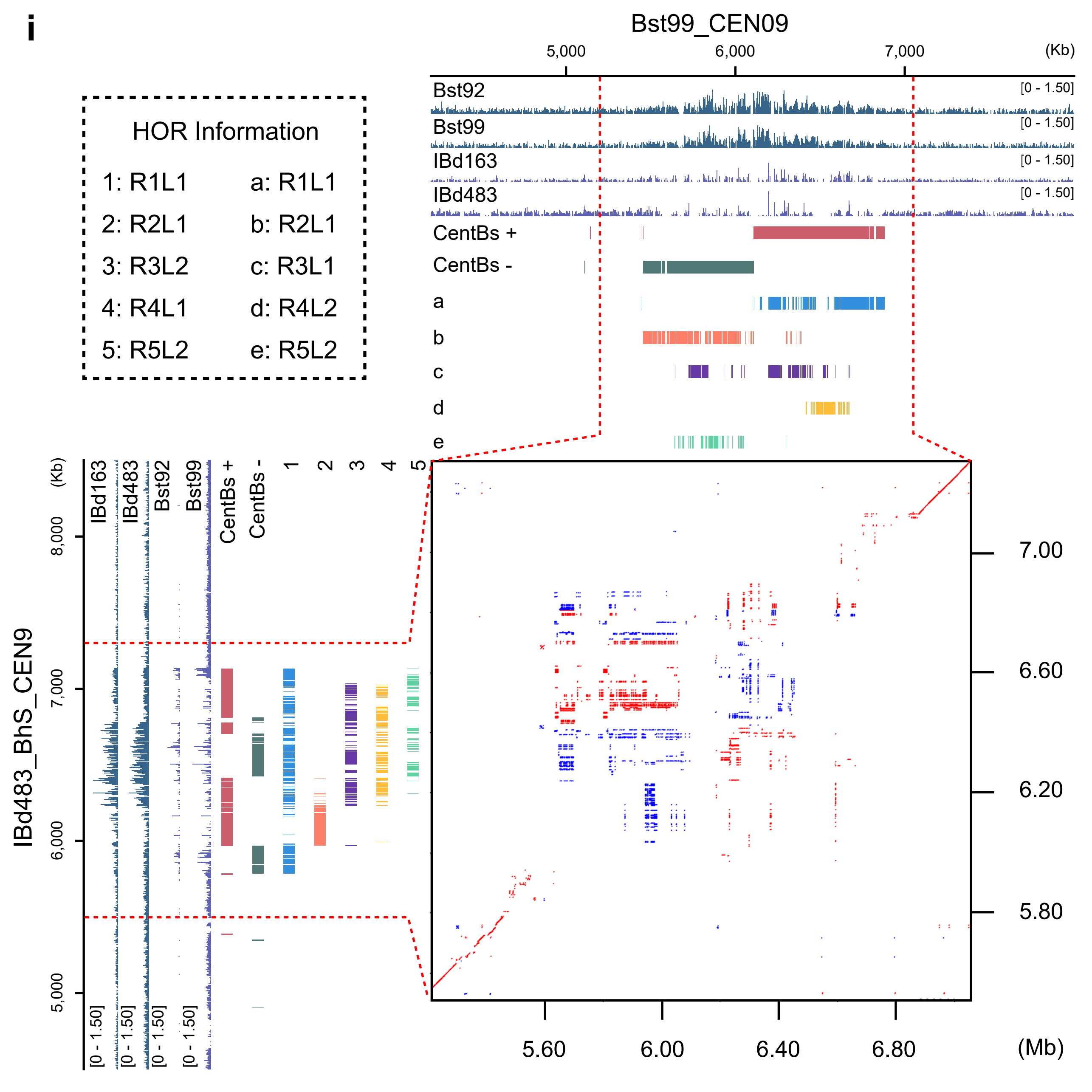
i
Bst99_CEN09
5,000
6,000
7,000
(Kb)
Bst92
Bst99
IBd163
IBd483
[0 - 1.50]
CentBs +
CentBs -
a
b
c
d
e
HOR Information
1: R1L1
2: R2L1
3: R3L2
4: R4L1
5: R5L2
a: R1L1
b: R2L1
c: R3L1
d: R4L2
e: R5L2
IBd483_BhS_CEN9
IBd163
IBd483
Bst92
Bst99
CentBs +
CentBs -
1
2
3
4
5
(Kb)
8,000
7,000
6,000
5,000
7.00
6.60
6.20
5.80
5.60
6.00
6.40
6.80
(Mb)

j

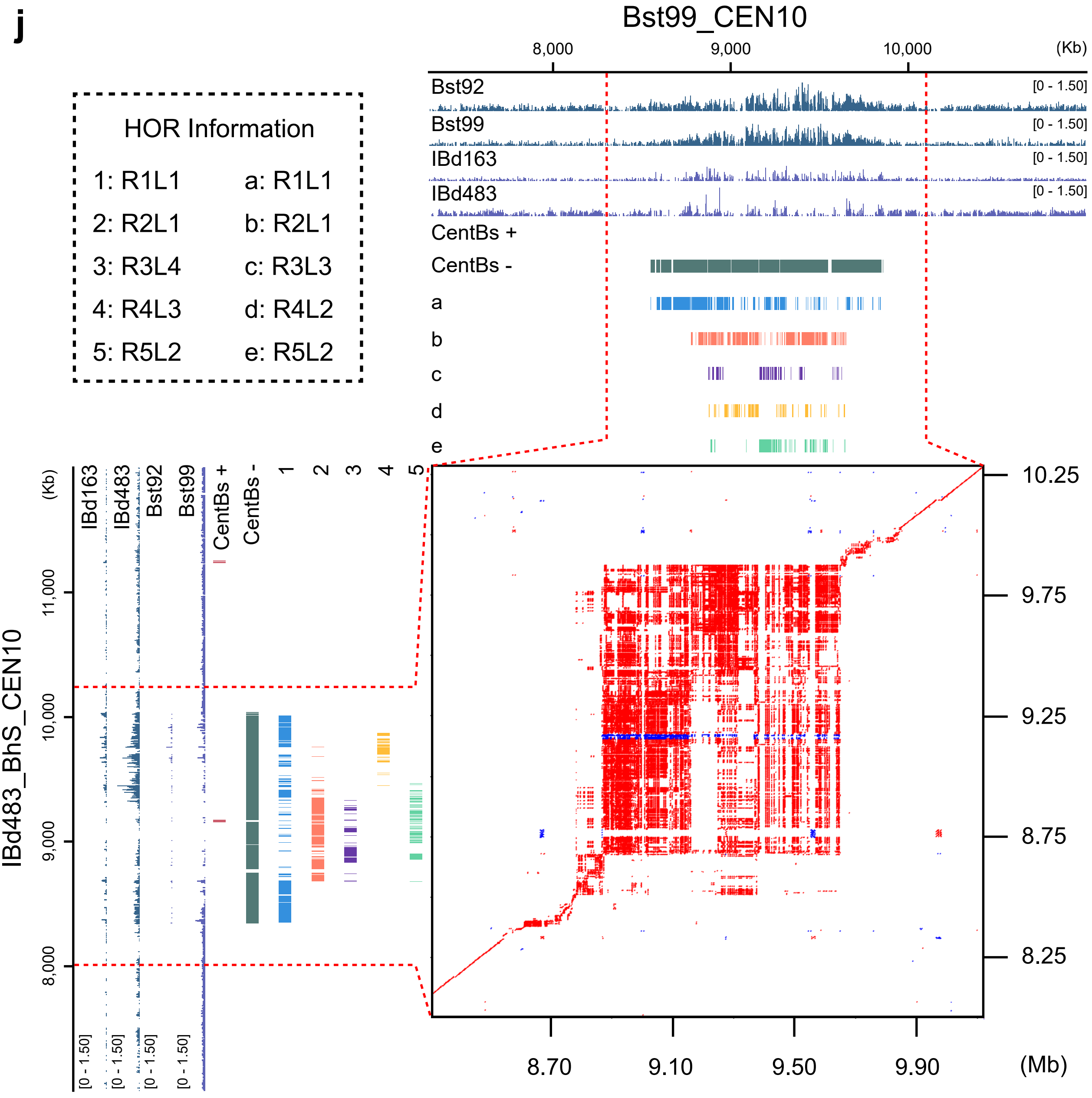

Bst99_CEN10
8,000
9,000
10,000
(Kb)
Bst92
Bst99
IBd163
IBd483
[0 - 1.50]
CentBs +
CentBs -
a
b
c
d
e
HOR Information
1: R1L1
2: R2L1
3: R3L4
4: R4L3
5: R5L2
a: R1L1
b: R2L1
c: R3L3
d: R4L2
e: R5L2
IBd483_BhS_CEN10
(Kb)
11,000
10,000
9,000
8,000
IBd163
IBd483
Bst92
Bst99
CentBs +
CentBs -
1
2
3
4
5
10.25
9.75
9.25
8.75
8.25
8.70
9.10
9.50
9.90
(Mb)

Fig. S22 Characterization of centromere sequence, structure and CENH3 enrichments between the homologous chromosome pairs from diploid to tetraploid in S sub/genomes.

a Bst99-CEN01 vs. IBd483-BhS-CEN1. **b** Bst99-CEN02 vs. IBd483-BhS-CEN2. **c** Bst99-CEN03 vs. IBd483-BhS-CEN3. **d** Bst99-CEN04 vs. IBd483-BhS-CEN4. **e** Bst99-CEN05 vs. IBd483-BhS-CEN5. **f** Bst99-CEN06 vs. IBd483-BhS-CEN6. **g** Bst99-CEN07 vs. IBd483-BhS-CEN7. **h** Bst99-CEN08 vs. IBd483-BhS-CEN8. **i** Bst99-CEN09 vs. IBd483-BhS-CEN9. **j** Bst99-CEN10 vs. IBd483-BhS-CEN10. CENH3 ChIP-seq mapping coverage from Bst92, Bst99, IBd163 and IBd483 to the Bst99-CEN genome with the coordinate at top. The mapping coverage from IBd163, IBd483, Bst92 and Bst99 to the IBd483-CEN genome with coordinate at left side. The satellite monomer and HOR array annotated on different centromeres (Track 5–11). Dot plots comparing the homologous centromere pairs between diploid to tetraploid using a search window of 156-bp. Red and blue indicate forward- and reverse-strand similarity, respectively.

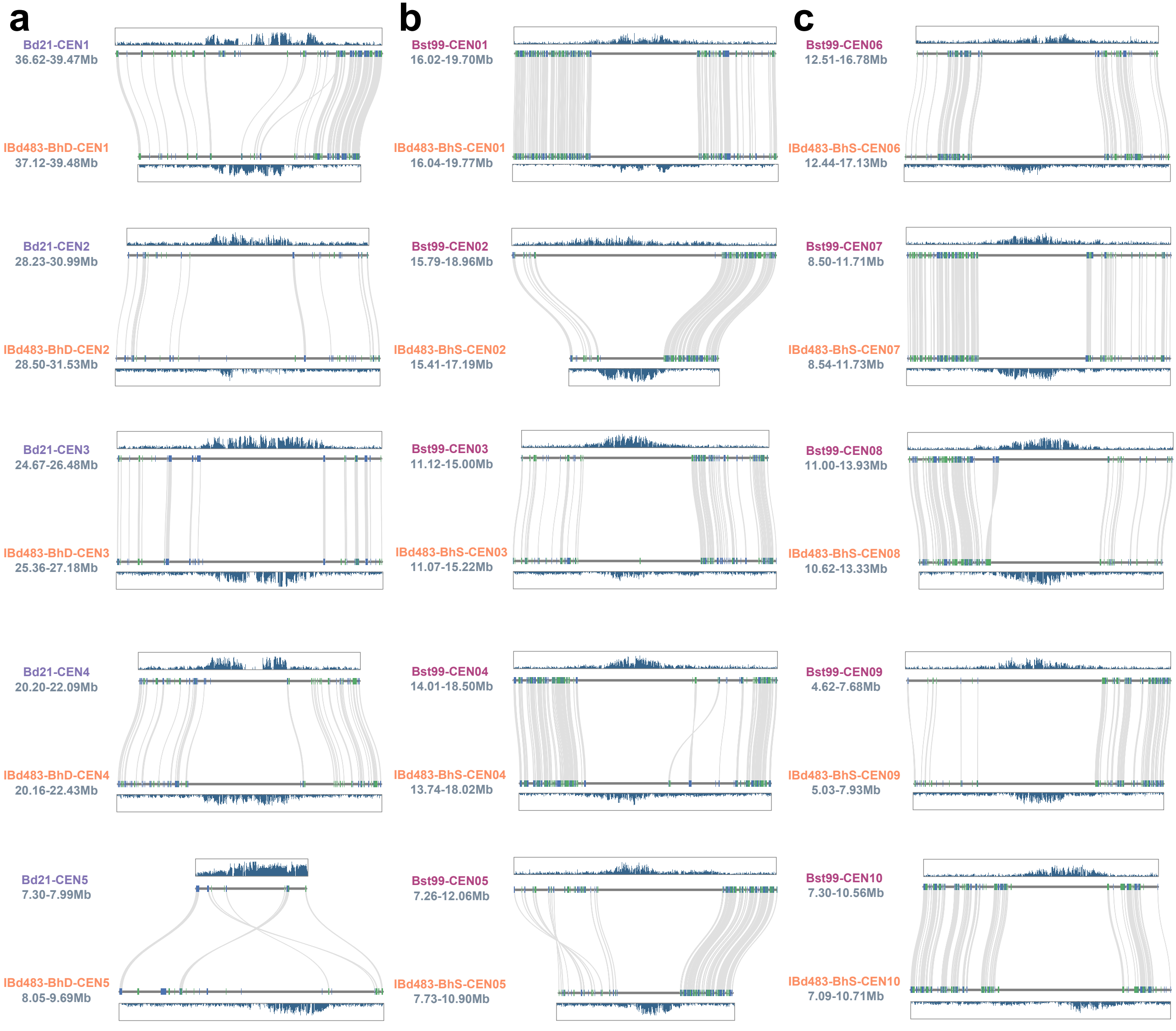

a
Bd21-CEN1
36.62-39.47Mb
IBd483-BhD-CEN1
37.12-39.48Mb
Bd21-CEN2
28.23-30.99Mb
IBd483-BhD-CEN2
28.50-31.53Mb
Bd21-CEN3
24.67-26.48Mb
IBd483-BhD-CEN3
25.36-27.18Mb
Bd21-CEN4
20.20-22.09Mb
IBd483-BhD-CEN4
20.16-22.43Mb
Bd21-CEN5
7.30-7.99Mb
IBd483-BhD-CEN5
8.05-9.69Mb
b
Bst99-CEN01
16.02-19.70Mb
IBd483-BhS-CEN01
16.04-19.77Mb
Bst99-CEN02
15.79-18.96Mb
IBd483-BhS-CEN02
15.41-17.19Mb
Bst99-CEN03
11.12-15.00Mb
IBd483-BhS-CEN03
11.07-15.22Mb
Bst99-CEN04
14.01-18.50Mb
IBd483-BhS-CEN04
13.74-18.02Mb
Bst99-CEN05
7.26-12.06Mb
IBd483-BhS-CEN05
7.73-10.90Mb
c
Bst99-CEN06
12.51-16.78Mb
IBd483-BhS-CEN06
12.44-17.13Mb
Bst99-CEN07
8.50-11.71Mb
IBd483-BhS-CEN07
8.54-11.73Mb
Bst99-CEN08
11.00-13.93Mb
IBd483-BhS-CEN08
10.62-13.33Mb
Bst99-CEN09
4.62-7.68Mb
IBd483-BhS-CEN09
5.03-7.93Mb
Bst99-CEN10
7.30-10.56Mb
IBd483-BhS-CEN10
7.09-10.71Mb

Fig. S23 Synteny alignments between homologous centromeres between diploid and tetraploid chromosomes.

a Bd21 vs. IBd483. **b-c** Bst99 vs. IBd483. The blue bars: the density of read mapping from CENH3 ChIP-Seq. The grey lines: the synteny gene pairs between Bd21/Bst99 and IBd483. The gray interval information represents the interval position selected for each chromosome.

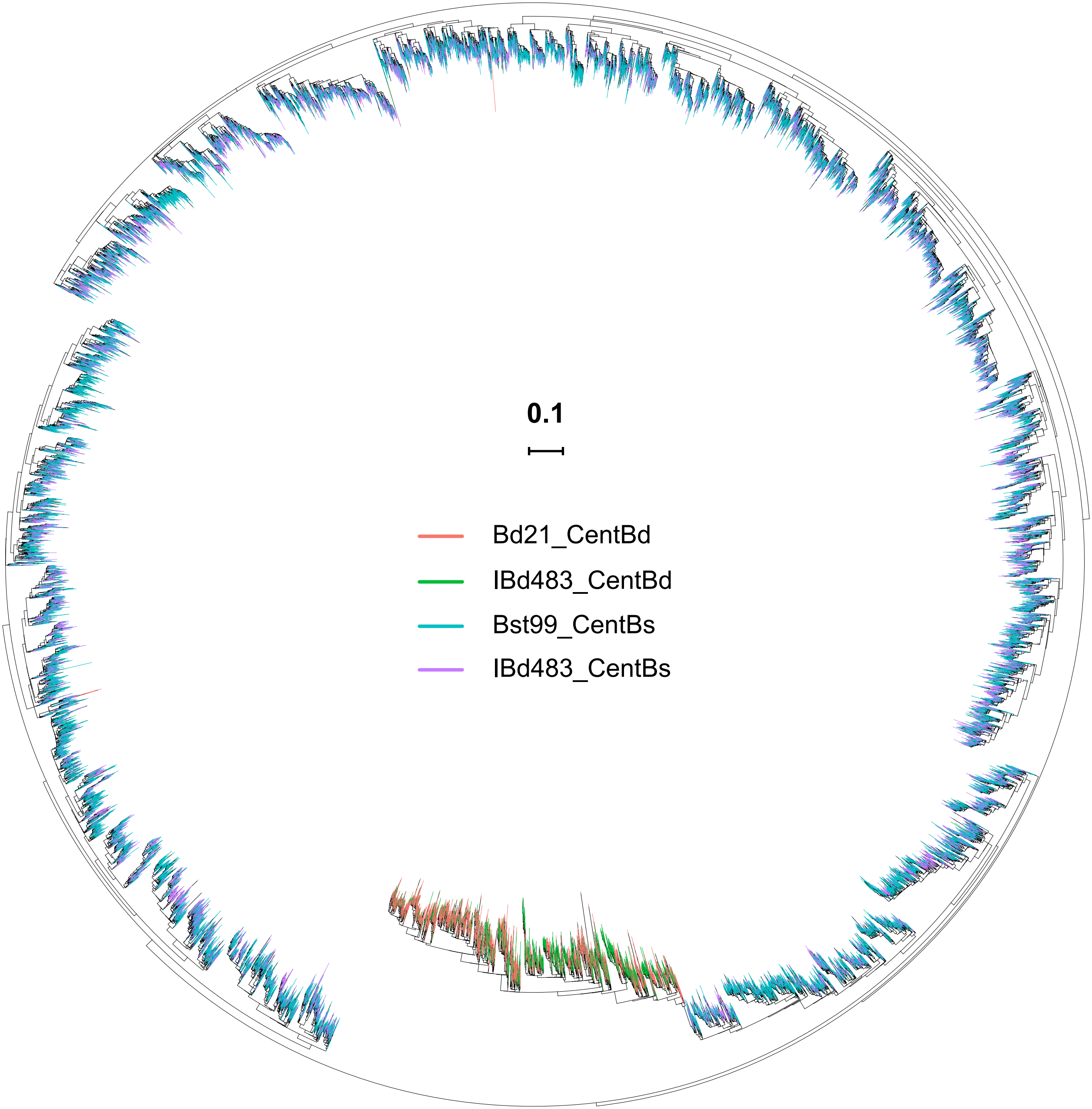
0.1
Bd21_CentBd
IBd483_CentBd
Bst99_CentBs
IBd483_CentBs

Fig. S24 Phylogenetic tree for CentBd or CentBs satellite repeats from each assembled *Brachypodium* sub/genome.

Phylogenetic tree for CentBd or CentBs satellite repeats from Bd21-CEN (light red), Bst99-CEN (cyan), BhD (green) and BhS (purple) subgenome of IBd483-CEN assemblies.

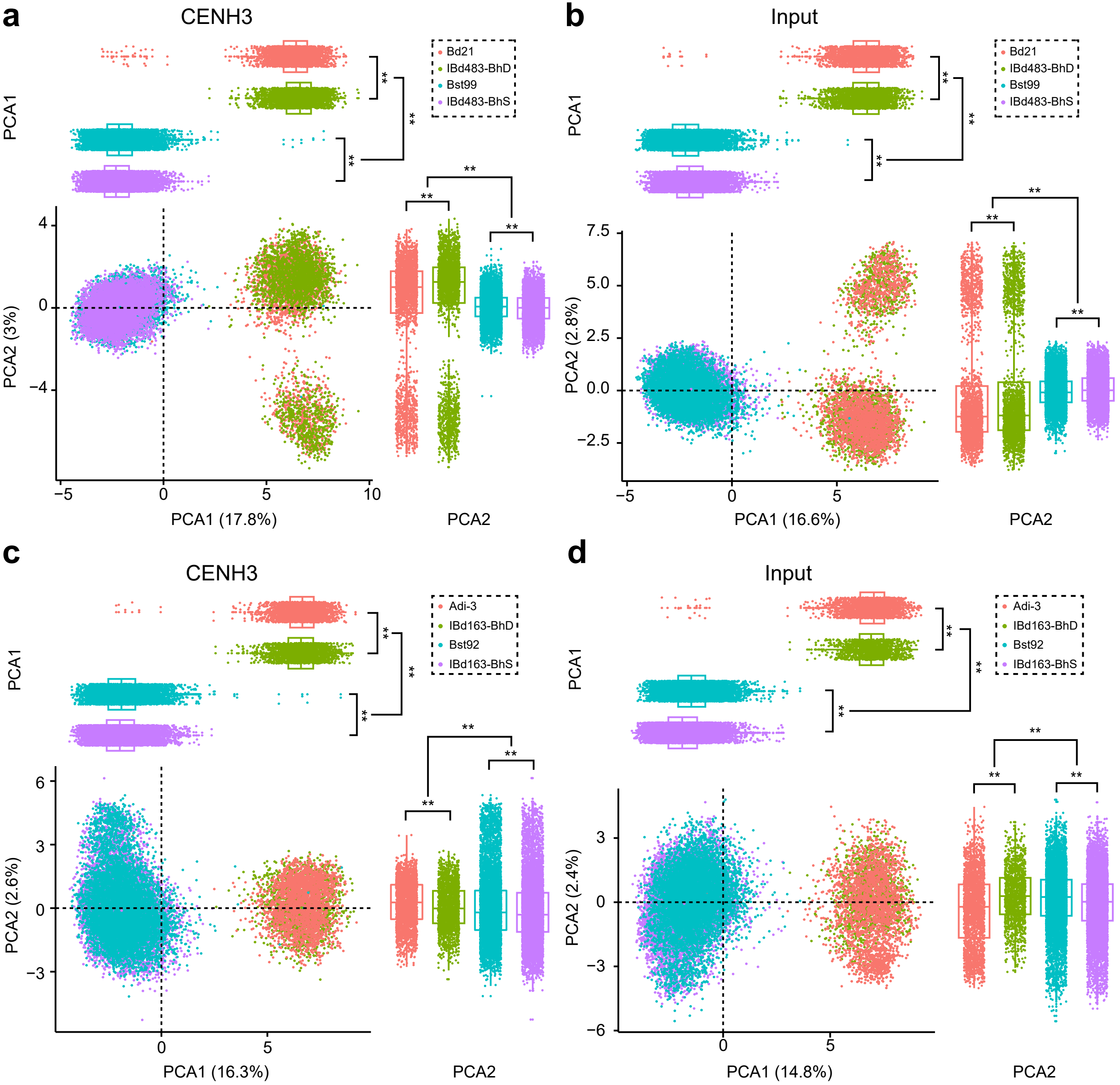
a
CENH3
Bd21
IBd483-BhD
Bst99
IBd483-BhS
PCA1
PCA2 (3%)
PCA1 (17.8%)
PCA2
b
Input
PCA2 (2.8%)
PCA1 (16.6%)
c
CENH3
Adi-3
IBd163-BhD
Bst92
IBd163-BhS
PCA2 (2.6%)
PCA1 (16.3%)
d
Input
PCA2 (2.4%)
PCA1 (14.8%)

Fig. S25 Comparisons of centromeric satellite repeats in *Brachypodium* sub/genomes between diploid and tetraploid chromosomes.

a-b IBd483, Bd21, Bst99. **c-d** IBd163, Adi-3, Bst92. PCA projection on principal components 1 and 2 of the normalized 5-mer frequency vectors for 10,000 randomly selected sequencing fragments covering satellite repeats from different lines of ChIP-seq or Input-seq samples. Each point represents an individual copy of the satellite repeat and is colored according to its genome or assigned subfamily based on hierarchical classification. Box plots on the top or right of the PCA projection show the overall distribution of PC1 and PC2 scores between different groups (T-test, ** $P<0.01$).

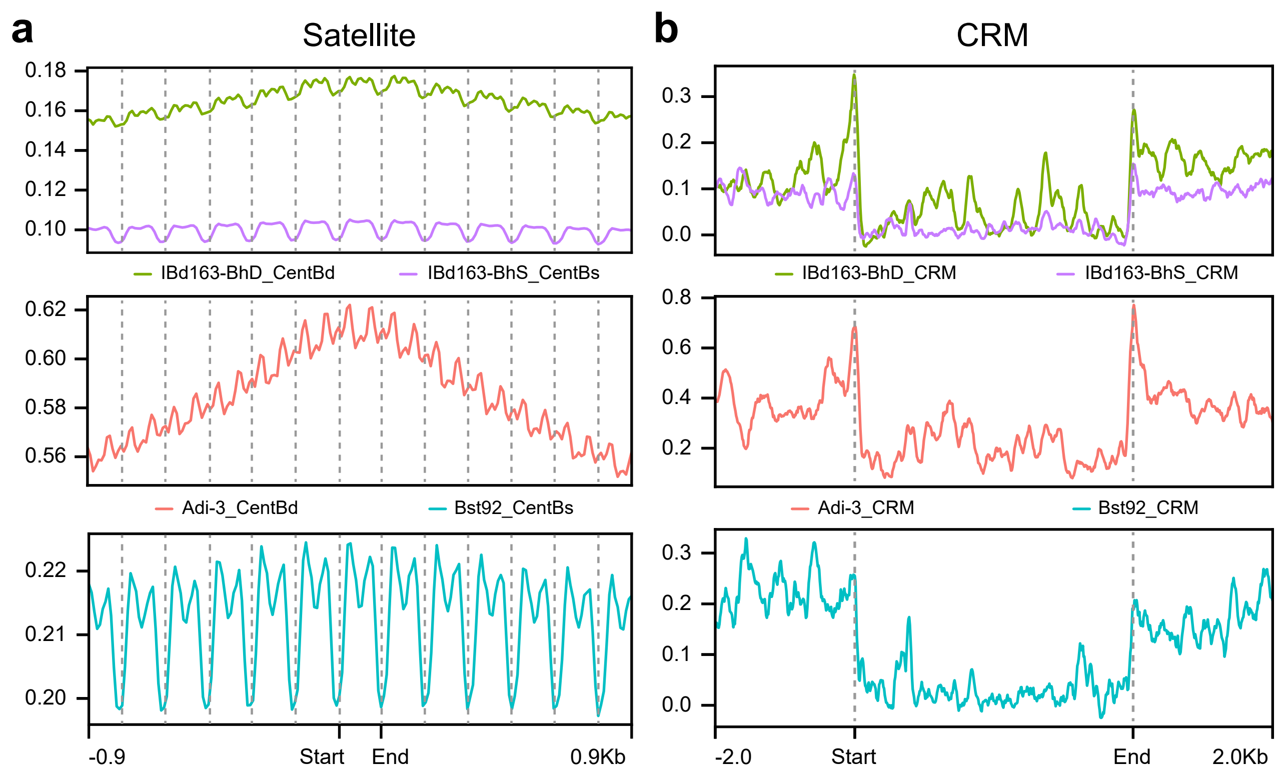

a
Satellite
0.18
0.16
0.14
0.12
0.10
IBd163-BhD_CentBd
IBd163-BhS_CentBs
0.62
0.60
0.58
0.56
Adi-3_CentBd
Bst92_CentBs
0.22
0.21
0.20
-0.9
Start
End
0.9Kb
b
CRM
0.3
0.2
0.1
0.0
IBd163-BhD_CRM
IBd163-BhS_CRM
0.8
0.6
0.4
0.2
Adi-3_CRM
Bst92_CRM
0.3
0.2
0.1
0.0
-2.0
Start
End
2.0Kb

Fig. S26 Characterization of CENH3 nucleosomes positioning on CentBd and CentBs satellite repeats in different *Brachypodium* genome.

a-b Metaprofiles of CENH3 signals (log_2(ChIP/Input)) from IBd163, Adi-3, and Bst92 around CentBd/CentBs **(a)** and the intact centromeric CRM repeats **(b)** between the D and S sub/genomes. The peaks reflect the distribution of CENH3 nucleosomes across the centromeric repetitive sequences. Different colored lines represent the CENH3 nucleosome distributions on CentBd/CRM repeat of Adi-3 (light red, Adi-3_CentBd/CRM), IBd163-BhD subgenome (light green, IBd163-BhD_CentBd/CRM) or on CentBs/CRM repeat of Bst92 (cyan, Bst92_CentBs/CRM), IBd483-BhS subgenome (purple, Ibd483-BhS_CentBd/CRM). The vertical axis represents the enrichment density of CENH3 ChIP-seq.

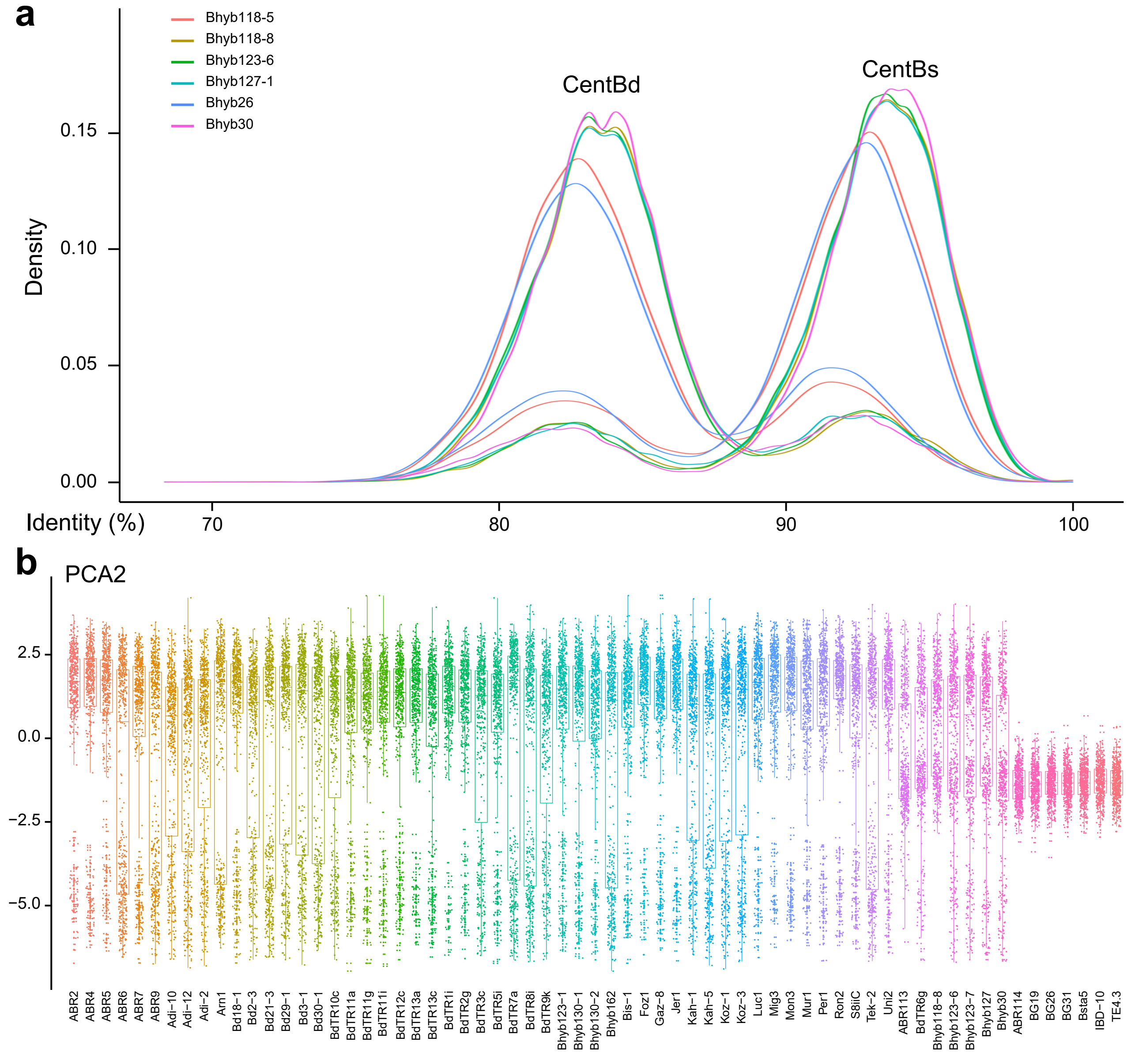
a
Bhyb118-5
Bhyb118-8
Bhyb123-6
Bhyb127-1
Bhyb26
Bhyb30
CentBd
CentBs
Density
0.15
0.10
0.05
0.00
Identity (%)
70
80
90
100
b
PCA2
2.5
0.0
-2.5
-5.0
ABR2
ABR4
ABR5
ABR6
ABR7
ABR9
Adi-10
Adi-12
Adi-2
Arn1
Bd18-1
Bd2-3
Bd21-3
Bd29-1
Bd3-1
Bd30-1
BdTR10c
BdTR11a
BdTR11g
BdTR11i
BdTR12c
BdTR13a
BdTR13c
BdTR1i
BdTR2g
BdTR3c
BdTR5i
BdTR7a
BdTR8i
BdTR9k
Bhyb123-1
Bhyb130-1
Bhyb130-2
Bhyb162
Bis-1
Foz1
Gaz-8
Jer1
Kah-1
Kah-5
Koz-1
Koz-3
Luc1
Mig3
Mon3
Mur1
Per1
Ron2
S8iiC
Tek-2
Uni2
ABR113
BdTR6g
Bhyb118-8
Bhyb123-6
Bhyb123-7
Bhyb127
Bhyb30
ABR114
BG19
BG26
BG31
Bsta5
IBD-10
TE4.3

Fig. S27 Characterization of centromeric repeat sequences in *Brachypodium pan-genome.*

a Sequence identity of merged fragments from WGS-seq reads to the CentBd or CentBs consensus sequence, as sampled in the pan-genomes of *B. hybridum* from different lines.

b Box plots show the overall distribution of PC2 scores among the individuals within each *Brachypodium* species.

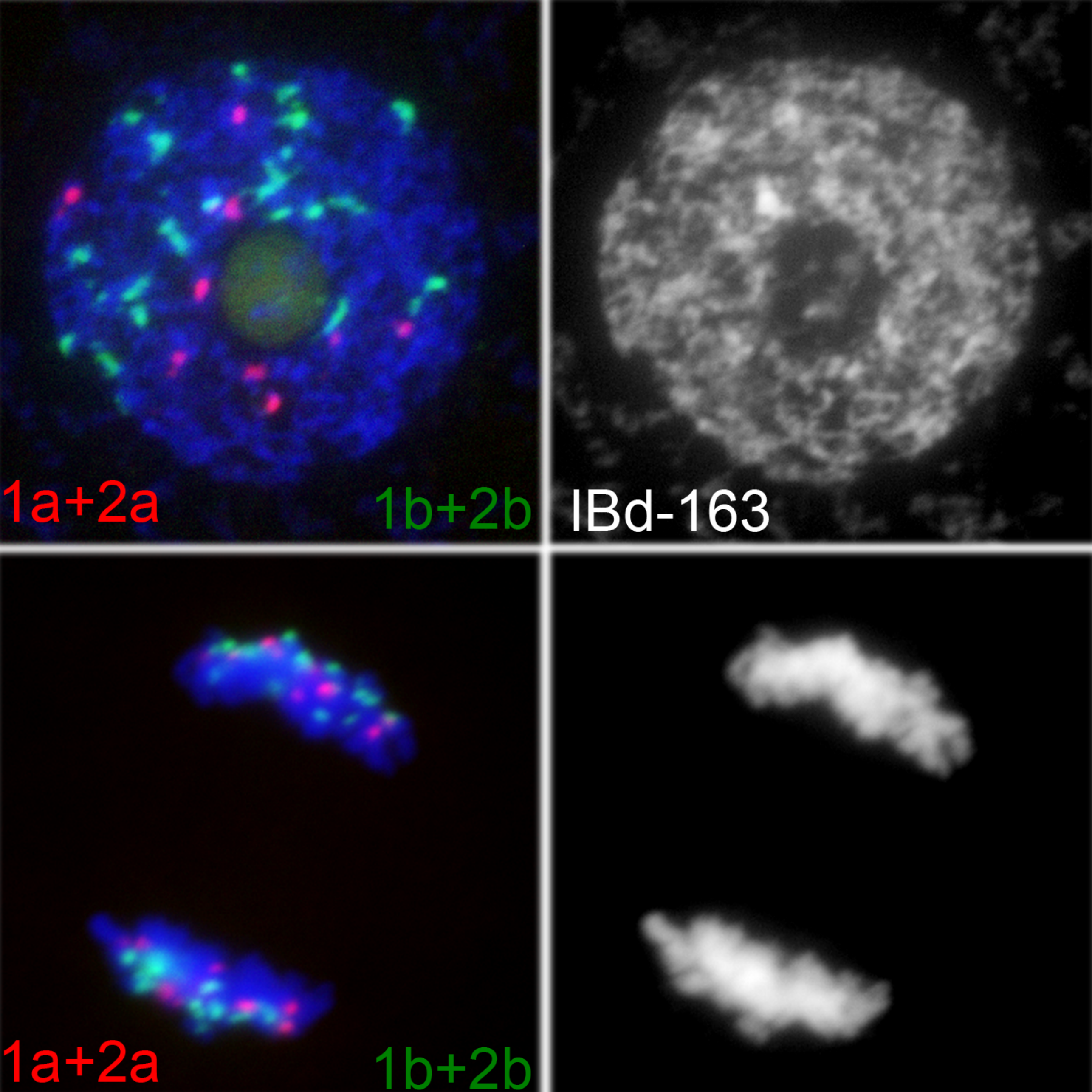
1a+2a
1b+2b
IBd-163
1a+2a
1b+2b

Fig. S28 Centromere behavior during early prophase I in IBd-163 line of *B. hybridum*.

a Zygotene. **b** Anaphase I. CentBd satellite repeats are colored in red and CentBs satellite repeats are colored in green. Blue indicates chromosomes counterstained with DAPI. Bar = 10 μm.
