# Supplementary material for: Three near-complete genome assemblies reveal substantial centromere dynamics from diploid to tetraploid in Brachypodium genus
Source: Genome Biol. 2024 Mar 4;25:63. doi: 10.1186/s13059-024-03206-w (PMC10910784; doi:10.1186/s13059-024-03206-w)
Supplement: Supplementary file 3 — Additional file 3. Review history. [file 13059_2024_3206_MOESM3_ESM.docx]

Review History

**First round of review**

**Reviewer 1**

**Were you able to assess all statistics in the manuscript, including the appropriateness of statistical tests used?**

No, I do not feel adequately qualified to assess the statistics.

**Comments to author:**

Chen and colleagues conducted a study comparing centromere diversity in Brachypodium hybridum and its two diploid ancestors. Previous research by Vogel et al. (2010) and Gordon et al. (2020) has highlighted the occurrence of large-scale genomic rearrangements during polyploidization in both Brachypodium hybridum and its diploid progenitors. For example, the research findings reveal that in Brachypodium hybridum, there are nested insertions of whole chromosomes, specifically in the centromeric regions, which contribute to the formation of chromosomes Bd1-Bd3 from B. distachyon. Additionally, chromosome Bd4 shows syntenic relationships with two B. stacei chromosomes (Bs5 and Bs10) as well as three rice chromosomes (Os9, Os11, and Os12). To gain a better understanding of Brachypodium polyploidization, Chen and colleagues could focus on comparing the centromere diversity associated with these unique genome architectures. This focused examination would likely yield further valuable insights into the intriguing process of Brachypodium polyploidization.

Major comments are as follows:

1. The genome assemblies generated from this study have increased novel sequences in comparison with previous genome sequencing results (Gordon et al., 2020). A comprehensive characterization of these sequencing differences between this work and previous studies could highlight the novelty of this study. For instance, are there any differences in genes or TEs?

2. The authors compared a single polyploid centromere to individual centromeres of the diploid progenitors and concluded the diversity of centromere sequences. The comparison is relatively simple. And it is hard to reach novel insights into the Brachypodium evolution. The authors could follow the genome comparison in the previous study (Gordon et al., 2020), systematically comparing the genomes of O. sativa, B. stacei, B. distachyon and B. hybridium.

3. Similarly, the analysis of satellite arrays and CRM elements could be conducted in the genomes of O. sativa, B. stacei, B. distachyon and B. hybridium following the genome comparison in the previous study (Gordon et al., 2020).

4. Centromere variations are usually associated with the DNA methylation level. The variations the authors observed might be associated with DNA methylation levels.

5. Again, the origin and evolution of centromeric satellite repeats should be analysed in all the genomes of O. sativa, B. stacei, B. distachyon and B. hybridium.

Minor comments:

1. The manuscript was piled up with very long descriptions where it lacked the key messages for each subsection. This is also why the authors could conduct their analysis following the evolutionary route of Brachypodium.

2. The figure legends are not clear, a lot of information should be added.

**Reviewer 2**

**Were you able to assess all statistics in the manuscript, including the appropriateness of statistical tests used?**

Yes, and I have assessed the statistics in my report.

**Comments to author:**

The authors have assembled almost complete genomes of three related Brachypodium species and compared the centromere sequences among them. Their data provided a great oppotunity to study the centromere evolution during polyploidization and will attract general interests of researchers in the area of genetics and evolution. However, I have difficulty reading through their MS as many paragraphs are full of trivial details but lack any conclusive or guiding sentences at the beginning or end of the paragraphs or sections. Maybe, this is because I am not expert in centromere. But I think it will have much wider impact if the MS can let the readers with little background knowledge on centromere also enjoy reading it.

In addition, I am confused about their statement about the presence of many repetive elements only on one strand of DNA but absence on the other strand. In theory, the DNA double helix should follow the AT/GC matching rule and any repeat sequences on one strand should also be found on the other but just in another form.

Also, maybe a cartoon summarizing their findings on centromere evolution will also help readers to clarify the relationships among different sections and get a better understanding of the whole paper.

**Reviewer 3**

**Were you able to assess all statistics in the manuscript, including the appropriateness of statistical tests used?**

Yes, and I have assessed the statistics in my report.

**Comments to author:**

The authors constructed 3 nearly complete genomes which allowed them to characterize the centromeric regions in detail. They showed that the centromeres in the allotetraploid species Brachypodium hybridum were highly conserved when compared to the diploid progenitors. Overall, the scientific conclusions were well supported by the data. However, I have serious concerns about the labelling of species, lack of acknowledgement of the source of germplasm and sequences, and, most concerning, the inclusion of two unpublished genomes, Brachypodium sylvaticum and B. mexicanum that are still under data embargo. At best these are multiple innocent errors and a lack of familiarity with standard practices and at worst attempts to obfuscate and the source of the data/germplasm. I will address these individually below.

The authors have used cryptic names for the species and genome assemblies throughout the manuscript. This not only makes it hard to understand which genomes they are talking about it also obscures the origin of the genomes. For example, it appears that the Bdi21 genome generated comes from the community standard line Bd21. They should use Bd21 as the name and add whatever descriptor they want for the assembly. Similarly, they refer to the current reference genomes for B. distachyon accession Bd21, B. hybridum accession ABR113 and B. stacei accession ABR114 with the filenames Bdistachyon_314_v3.0 , Bhybridum_463_v1.0 and Bstacei_316_v1.0, respectively, of the genomes they downloaded. They should use Bd21, ABR113 and ABR114 as the names and note the assembly version. It may be helpful to readers to include any file names or links to the genomes in the methods. In table S19 many of the accession names are incorrect. They may have used names that were in NCBI or another database that were incorrect. Consulting the papers they were published in would reveal the errors. In addition, they include two entries for line TE4.3. There is only one TE4.3 it appears that the second entry is simply a duplicate sequence. They also list line BdTR6g-VSM. The line should be BdTR6g. The -VSM in two line names appears to be a sequence sample designation. If they wish to include sample information, they should have a separate column for that. Here is a list of incorrect names with the corrections:

Adi_10 Adi-10

Adi_12 Adi-12

Adi_2 Adi-2

Bd18_1 Bd18-1

Bd2_3 Bd2-3

Bd21_3 Bd21-3

Bd29_1 Bd29-1

Bd3_1 Bd3-1

Bis_1 Bis-1

Gaz_8 Gaz-8

Kah_1 Kah-1

Kah_5 Kah-5

Koz_1 Koz-1

Koz_3 Koz-3

Tek_2 Tek-2

BdTR6g-VSM BdTR6g

TE4_VSM TE4.3

They have not cited the source of the germplasm used. I notice that the B. hybridum and B. stacei lines came from Israel, but I did not see a reference or a collaborator who collected the seeds. They should include the source of all germplasm used in the materials and methods and provide a citation or indicate that they have permission of the collector. This is standard practice in the scientific community. This should also be included in Table s19.

They should also cite the sources of the sequences used and not just indicate the source as NCBI or some other database. For example, I believe they used sequences from the Brachypodium pangenome paper by Gordon et al., and the Brachypodium genome paper.

They included whole genome analysis of two unpublished genomes, B. sylvaticum and B. mexicanum. Thes genomes are still under restricted use as described on the genome pages in Phytozome. They would have had to see these restrictions when they downloaded the sequences from Phytozome. If they want to include these analyses, they should contact the project leaders listed on the Phytozome genome pages for these organisms:

https://phytozome-next.jgi.doe.gov/info/Bmexicanum_v2_1

https://phytozome-next.jgi.doe.gov/info/BsylvaticumAin_1_v2_1

They did not do a de-novo annotation of non-TE genes. Rather, they used a lift over from the existing reference genomes. This will miss genes for the B. hybridum and B. stacei genomes due to intraspecific variation. While this is irrelevant for their centromere analysis, they should note this limitation.

They have an excessive number of supplementary tables and figures. I encourage them to reduce the number to those needed to convey the main findings. For example, they really don't need k-mer graphs to estimate genome sizes for these well-studied species.

Specific comments

They should mention if IBd483 is a newer or older type of B. hybridum.

Figure S4 a&b it looks like the numbering of some chromosomes are inverted because the orientation of the line on the dot plots are going in different directions. They should fix this.

Figure S14 is very hard to understand.

Fig S22 remove the black lines or make them under the colored lines so you can see the trends easier. Currently, you have to zoom way in to see the colors.

Page 2 line 26 Fix the sentence containing "resulting the frequency"

Page 3 line 27 delete "arising from wide hybridization" because polyploids can also arise from genome duplication without a wide cross, autopolyploidy.

Page 8 line 25 change descripted to described.

Page 9 line 19 awkward sentence

Page 9 line 35 add "the" between of and satellite.

Page 22 change comprehensive to comprehensively.

Page 22 line 54 define "natural conditions".

Page 23 line 31 define "certain amount".

Page24 line 19 add reference for Bd21 genome.

Page 28 line 19 delete "Metadata of".

Figure 1a the innermost synteny plot is useless. Either delete or add individual colors for different chromosomes. Also, it appears they only indicate synteny between certain genomes/subgenomes. They should mention this.

Figure 3e I am not sure what the Q scale means.

Figure 4 g-h it is unclear top me what this is.

Figure 5 b I'm not sure what the y axes designate.

Table 1 the listed BUSCO scores may be of a little since they did a lift over annotation. However, if anything they would be improved with a proper annotation.

**Authors Response**

**Point-by-point responses to the reviewers’ comments:**

**Reviewer #1:**

Chen and colleagues conducted a study comparing centromere diversity in Brachypodium hybridum and its two diploid ancestors. Previous research by Vogel et al. (2010) and Gordon et al. (2020) has highlighted the occurrence of large-scale genomic rearrangements during polyploidization in both Brachypodium hybridum and its diploid progenitors. For example, the research findings reveal that in Brachypodium hybridum, there are nested insertions of whole chromosomes, specifically in the centromeric regions, which contribute to the formation of chromosomes Bd1-Bd3 from B. distachyon. Additionally, chromosome Bd4 shows syntenic relationships with two B. stacei chromosomes (Bs5 and Bs10) as well as three rice chromosomes (Os9, Os11, and Os12). To gain a better understanding of Brachypodium polyploidization, Chen and colleagues could focus on comparing the centromere diversity associated with these unique genome architectures. This focused examination would likely yield further valuable insights into the intriguing process of Brachypodium polyploidization.
***Reply: We thank the reviewer for the valuable and constructive comments and suggestions. In fact, the presence of nested insertions of whole chromosomes into centromeres or other chromosome rearrangements from a common ancestor occurred in diploid Brachypodium species. Chromosome structure have not undergone large-scale changes during Brachypodium polyploidization process (Additional file 1: Fig. S3). We have further conducted a comprehensive comparison of the centromere diversity within these unique genome architecture of Brachypodium and O. sativa genomes. Subsequent to the divergence of new species from a common ancestor through processes like nested insertions of whole chromosomes, we found rapid interspecies centromere evolutions and complete turnover accompanied with or after speciation.*** ***Please find our detailed responses below.***

Major comments are as follows:
1. The genome assemblies generated from this study have increased novel sequences in comparison with previous genome sequencing results (Gordon et al., 2020). A comprehensive characterization of these sequencing differences between this work and previous studies could highlight the novelty of this study. For instance, are there any differences in genes or TEs?

***Reply: Thank you for your suggestion. We agree with you that a detailed comparison between our new assemblies and previously reported genomes would highlight the novelty of this study. Firstly, when compared to earlier genome assemblies, our new genomes introduced approximately 22.59 Mb, 4.43 Mb, and 16.53 Mb of novel sequences within or near the centromeres (Additional file 1: Fig S2). The contig N50 values of our newly assembled genomes are 26.5, 2010, and 77.9 fold longer than those of the earlier versions, respectively. The gap numbers of previous genomes exhibit a greater number of gaps compared to our newly assembled genomes (Additional file 1: Table S3). These advantages could be attributed to the development of sequencing technology, particularly the adaptation of the highly accurate long-read HiFi sequencing and Hi-C techniques. Secondly, we re-annotated the TEs for the previous genomes using the same pipeline. We found that the numbers of intact TEs in our newly assembled genomes were much higher than those in previous genomes (Additional file 1: Table S5), which also indicates our newly assembled genomes have better quality. We still think the earlier genomes by Gordon et al. as valuable resources for our community, especially in terms of the detailed gene annotations. However, our focus is on conducting a comprehensive comparative analysis of previously inaccessible centromere regions across species with diverse polyploidy levels. Thirdly, we identified inversions and translocations through whole genome alignment using minimap2 tool (https://github.com/lh3/minimap2). We found numbers of inversions and translocations between ours and Gordon et at. [1,2] (Additional file 1: Fig S2). And we double-checked the chromosome interactions from our Hi-C data, the orders and orientations of the sequence of inversions and translocation in* *our genomes were accurate. In addition, based on a more* *sensitive alignment of genomes (https://github.com/baoxingsong/anchorwave), we detected insertions and deletions by Assemblytics (https://github.com/marianattestad/assemblytics). Massive small insertions and deletions were detected, and tended to appear in centromere regions (Additional file 1: Fig S2). Nevertheless, we are not sure if these differences were caused by the different qualities of ours and previously assembles or the line-specific. Finally, we decided not to include SV results in the article.***

***Fig. S2 Syntenic alignment and structural variations comparisons of new genome assemblies and their previous reference genomes.***

***a-c*** *Alignments showing collinearity (gray), inversion (orange) and translocation (green) structural variations between Bd21 (v3.1) chromosomes and those of Bd21-CEN genome* ***(a)****, ABR114 (v1.1) chromosomes and those of Bst99-CEN genome (****b****), ABR113 (v1.1) chromosomes and those of IBd483-CEN genome (****c****). Distributions of insertion length were plotted (blue) along the chromosome in each genome. Centromere position was indicated with red box.*

***Reference:***

*(1). Gordon SP, Contreras-Moreira B, Levy JJ, Djamei A, Czedik-Eysenberg A, Tartaglio VS, Session A, Martin J, Cartwright A, Katz A, et al: Gradual polyploid genome evolution revealed by pan-genomic analysis of Brachypodium hybridum and its diploid progenitors. Nat Commun 2020, 11:3670.*

*(2). Gordon SP, Contreras-Moreira B, Woods DP, Des Marais DL, Burgess D, Shu S, Stritt C, Roulin AC, Schackwitz W, Tyler L, et al: Extensive gene content variation in the Brachypodium distachyon pan-genome correlates with population structure. Nat Commun 2017, 8:2184.*

2. The authors compared a single polyploid centromere to individual centromeres of the diploid progenitors and concluded the diversity of centromere sequences. The comparison is relatively simple. And it is hard to reach novel insights into the Brachypodium evolution. The authors could follow the genome comparison in the previous study (Gordon et al., 2020), systematically comparing the genomes of O. sativa, B. stacei, B. distachyon and B. hybridium.

***Reply: Thank you for your suggestion. The chromosomal structure did not undergone large-scale changes during Brachypodium polyploidization process (Additional file 1: Fig. S3), and we have indeed compared centromere dynamics during the polyploidization process (Figure 2 and 4). We further conducted a comprehensive comparison of the centromere diversity within these unique genome architectures across diploid Brachypodium and O. sativa genomes. Earlier research had highlighted the presence of nested insertions of whole chromosomes, particularly within centromeric regions, contributing to the formation of novel chromosomes in Brachypodium [1, 2]. However, through the examination of centromere dot-plots, we observed an absence of centromere sequence and structural conservations between these shared unique chromosome pairs (Bd1-Bs7-Os6; Bd2-Bs8-Os5; Bd3-Bs3-Os10; Bd4-Bs5/Bs10-Os11; Bd5-Bs9-Os4, Additional file 1: Fig. S10), suggesting the rapid interspecies centromere evolutions and complete turnover are accompanied with or after speciation. These results are consist with the understanding that centromeres represent the most intricate genome structure and evolved dramatically, generating complex genetic diversity over the course of evolution.***

***In fact, genomic variations and adaptations within centromeres during polyploidization process is important to the stability of polyploid, and is also a significant biological concern in the evolutionary field. Our studies goes beyond the simple comparison of a single polyploid centromere against the individual centromeres of the diploid progenitors. We also delved into the variations of satellite repeats using the pan-genome data across different polyploidy levels. Our findings unveil shared characteristics of centromere evolution in tetraploid Brachypodium, and provide novel insights into the dynamic (epi)genetics of centromeres. The turnover of centromeres are closely associated between homologous chromosomal pairs from diploid to tetraploid.*** ***These profound findings contribute to a richer understanding of the intricate dynamics that govern centromere evolution in polyploidy and its implications for the broader field of evolution and speciation. These results are thoroughly added in the discussion of the manuscript.***

***Fig. S10 Dotplot comparing the syntenic centromeres among O. sativa, B. stacei, and B. distachyon genome using a search window of 156-bp.***

***a*** *CEN1 (B. distachyon)-CEN07 (B. stacei)-Os6 (O. sativa);* ***b*** *CEN2-CEN08-Os5;* ***c*** *CEN3-CEN03-Os10;* ***d*** *CEN4-CEN05/10-Os11;* ***e*** *CEN5-CEN09-Os4.*

***Reference:***

*(1). International Brachypodium Initiative. Genome sequencing and analysis of the model grass Brachypodium distachyon. Nature. 2010, 11;463(7282):763-8.*

*(2). Gordon SP, Contreras-Moreira B, Levy JJ, Djamei A, Czedik-Eysenberg A, Tartaglio VS, Session A, Martin J, Cartwright A, Katz A, et al: Gradual polyploid genome evolution revealed by pan-genomic analysis of Brachypodium hybridum and its diploid progenitors. Nat Commun 2020, 11:3670.*

3. Similarly, the analysis of satellite arrays and CRM elements could be conducted in the genomes of O. sativa, B. stacei, B. distachyon and B. hybridium following the genome comparison in the previous study (Gordon et al., 2020).
***Reply: Thank you for your suggestion. We have added further comparisons of the corresponding centromeres among O. sativa, B. stacei, and B. distachyon genomes. Our phylogenetic analysis of satellite arrays and CRM elements has yielded similar results (Additional file 1: Fig. S12). Notably, we did not identify any direct relationships of centromeric repeat sequences between these unique centromere architectures. For example, Bd4 is syntenic with two B. stacei chromosomes, Bs5 and Bs10. CentBd satellites or CR elements from Bd4 did not clustered together with those CentBs or CR repeats from Bs5/Bs10 chromosomes. Furthermore, the relationship between B. distachyon chromosomes Bd1 to Bd3 and the corresponding B. stacei chromosomes can be explained by a series of nested insertions of whole ancestral chromosome equivalents into centromeric regions (Bd1: Bs7→Bs6→Bs2; Bd2: Bs8→Bs1; Bd3: Bs3→Bs4). The results also revealed no closer associations for these corresponding chromosomes. These repetitive sequences clustered together based on their species, suggesting that each species* *acquired private centromere* *satellite and CR populations.***

***These findings underscore the notion that subsequent to the divergence of new species from a common ancestor through processes like nested insertions of whole chromosomes or other chromosome rearrangements, centromere sequences undergo complete turnover.*** ***This study also unveils the distinctive attributes of centromeres across genomes, suggesting the emergence of a distinct repertoire of centromeric repeats exclusive to each species.***

***Fig. S12 Satellite repeat (a) and retrotransposon element (b) library analyzed by syntenic centromeres between O. sativa and Brachypodium species.***

*Syntenic centromeres between O. sativa and Brachypodium species were listed in Fig. S10. The phylogenic tree colored by different chromosomes was generated by neighbor-joining methods.*

4. Centromere variations are usually associated with the DNA methylation level. The variations the authors observed might be associated with DNA methylation levels.
***Reply: Thank you for your suggestion. Studies in Arabidopsis have yielded insights into the distinct methylation patterns within centromeric satellite repeats [1]. Specifically, these results have illuminated the prevalence of CG-context methylation in centromeres, which stands in contrast to the methylation landscape outside of CG context. This has been demonstrated for the complex interplay between CENH3 spacer regions, DNA methylation, and the diverse CEN180 variants. The intriguing correlation has raised the possibility that CENH3-nucleosomes might exert an influence on epigenetic modifications and the variation of satellite sequences [1]. Our examination extends to the differentiation of satellite repeats across the Brachypodium genus. Notably, we have observed slight different positioning of CENH3 nucleosomes between CentBd and CentBs satellite repeats from D and S sub/genomes. We also considered that DNA methylation patterns likely be different between these differentiated satellite sequences among the Brachypodium genus. However, the precise causal relationship between these variations in nucleosome positioning, the divergence of satellite sequences, and potential distinct DNA methylation remains enigmatic.***

***Our primary focus is on conducting a comprehensive comparative analysis of previously inaccessible centromere regions across species with varying polyploidy levels. Since these experiments would take several months to complete, we hope that the reviewer agrees that they are beyond the scope of the current manuscript. To fortify the depth of our discussion, we have supplemented the manuscript with an expanded discourse on the potential associations that could be drawn between centromere variations and the levels of DNA* *methylation.***

***Reference:***

*(1). Naish M, Alonge M, Wlodzimierz P, Tock AJ, Abramson BW, Schmucker A, Mandakova T, Jamge B, Lambing C, Kuo P, et al: The genetic and epigenetic landscape of the Arabidopsis centromeres. Science 2021, 374:eabi7489.*

5. Again, the origin and evolution of centromeric satellite repeats should be analysed in all the genomes of O. sativa, B. stacei, B. distachyon and B. hybridium.
***Reply: Thank you for your suggestion. The origins and evolutionary trajectories of centromeric satellite repeats are undoubtedly pivotal inquiries in our understanding of genome evolution. Previous investigations have delved into this subject through comparative analysis of tandem repeats across a multitude of species, yielding valuable insights into the dynamic evolution of centromeres. However, the findings from these sequence homology-based phylogenetic analysis have unveiled limited evidence of satellite sequence conservation beyond approximately 50 million years of divergence [1].***

***Our research, focusing on the centromeric satellite repeats within closely related Brachypodium genomes (including B. stacei, B. distachyon, B. hybridium, and B. mexicanum), has contributed to this discourse. We have found that diverged centromeric satellite repeats among these sub/genomes, indications of the emergence and accumulation of species-specific centromeres that trace back to a common origin during the evolution of the Brachypodium genus. To expand our lens to encompass a broader evolutionary context, we embarked on the scrutiny of satellite repeat sequences across various species (including O. sativa, B. stacei, B. distachyon; Additional file 1: Fig. S12). Our analysis has substantiated the notion of species-specific centromere satellites emergence and accumulation, underpinned by a shared ancestral foundation during the evolution of species. However, the intricate journey that underlies the origin and evolutionary pathways of centromeric satellite repeats remains an enigma. As we focus on the comprehensive comparison of hitherto inaccessible centromeric regions across closely related species and polyploidy with unprecedented base-pair resolution. The detailed unraveling of the origin and evolution of satellite repeats at larger species scales based on the T2T genomes is another topic for future research and could potentially be explored in a separate manuscript.***

***Reference:***

*1. Melters DP, Bradnam KR, Young HA, Telis N, May MR, Ruby JG, Sebra R, Peluso P, Eid J, Rank D, Garcia JF, DeRisi JL, Smith T, Tobias C, Ross-Ibarra J, Korf I, Chan SW. Comparative analysis of tandem repeats from hundreds of species reveals unique insights into centromere evolution. Genome Biol. 2013 Jan 30;14(1):R10. doi: 10.1186/gb-2013-14-1-r10. PMID: 23363705; PMCID: PMC4053949.*

**Minor comments:**
1. The manuscript was piled up with very long descriptions where it lacked the key messages for each subsection. This is also why the authors could conduct their analysis following the evolutionary route of Brachypodium.
***Reply: Thank you for your suggestion. We have added some conclusive and guiding sentences at the beginning or end of certain paragraphs.***

2. The figure legends are not clear, a lot of information should be added.
***Reply: Thank you for your suggestion. We have added more information in the figure legends.***

Reviewer #2: The authors have assembled almost complete genomes of three related Brachypodium species and compared the centromere sequences among them. Their data provided a great opportunity to study the centromere evolution during polyploidization and will attract general interests of researchers in the area of genetics and evolution. However, I have difficulty reading through their MS as many paragraphs are full of trivial details but lack any conclusive or guiding sentences at the beginning or end of the paragraphs or sections. Maybe, this is because I am not expert in centromere. But I think it will have much wider impact if the MS can let the readers with little background knowledge on centromere also enjoy reading it.

***Reply: We thank the reviewer for their positive comments. We have incorporated some conclusive or guiding sentences at the beginning or end of certain paragraphs or sections throughout the manuscript.***

In addition, I am confused about their statement about the presence of many repetive elements only on one strand of DNA but absence on the other strand. In theory, the DNA double helix should follow the AT/GC matching rule and any repeat sequences on one strand should also be found on the other but just in another form.
***Reply: Centromeric satellite arrays consist of extensive stretches of tandemly organized repeated sequences, oriented in a head-to-tail manner. In instances where satellite array 1 exhibits a head-to-tail orientation on the forward strand, it is possible for satellite array 2, situated elsewhere, to display a reversed orientation. This entails that the complementary reverse DNA strand will maintain identical head-to-tail alignments in comparison to the forward strand of satellite array 1, as illustrated in the accompanying figure. It's important to emphasize that our assertion regarding the presence of numerous repetitive elements on one DNA strand does not imply their absence on the complementary strand; instead, they are present but exhibit a reversed direction.***

Also, maybe a cartoon summarizing their findings on centromere evolution will also help readers to clarify the relationships among different sections and get a better understanding of the whole paper.
***Reply: Thank you for your suggestions. We have added a cartoon to summarizing our finding as Figure 6 in the manuscript.***

**Fig. 6 Evolution model of the dynamic centromeres in Brachypodium genus.**

**Reviewer #3:**

The authors constructed 3 nearly complete genomes which allowed them to characterize the centromeric regions in detail. They showed that the centromeres in the allotetraploid species Brachypodium hybridum were highly conserved when compared to the diploid progenitors. Overall, the scientific conclusions were well supported by the data. However, I have serious concerns about the labelling of species, lack of acknowledgement of the source of germplasm and sequences, and, most concerning, the inclusion of two unpublished genomes, Brachypodium sylvaticum and B. mexicanum that are still under data embargo. At best these are multiple innocent errors and a lack of familiarity with standard practices and at worst attempts to obfuscate and the source of the data/germplasm. I will address these individually below.

***Reply: We are sorry for the innocent errors and a lack of familiarity with standard practices to use the source of the data/germplasm. We have rectified these issues accordingly. The accurate names for the species and genome assemblies have been updated as per standard conventions. Furthermore, we have diligently cited the appropriate sources for all germplasm and unpublished genome data referenced in the manuscript.***

The authors have used cryptic names for the species and genome assemblies throughout the manuscript. This not only makes it hard to understand which genomes they are talking about. It also obscures the origin of the genomes. For example, it appears that the Bdi21 genome generated comes from the community standard line Bd21. They should use Bd21 as the name and add whatever descriptor they want for the assembly. Similarly, they refer to the current reference genomes for B. distachyon accession Bd21, B. hybridum accession ABR113 and B. stacei accession ABR114 with the filenames Bdistachyon_314_v3.0, Bhybridum_463_v1.0 and Bstacei_316_v1.0, respectively, of the genomes they downloaded. They should use Bd21, ABR113 and ABR114 as the names and note the assembly version. It may be helpful to readers to include any file names or links to the genomes in the methods. In table S19 many of the accession names are incorrect. They may have used names that were in NCBI or another database that were incorrect. Consulting the papers they were published in would reveal the errors. In addition, they include two entries for line TE4.3. There is only one TE4.3 it appears that the second entry is simply a duplicate sequence. They also list line BdTR6g-VSM. The line should be BdTR6g. The -VSM in two line names appears to be a sequence sample designation. If they wish to include sample information, they should have a separate column for that. Here is a list of incorrect names with the corrections:
Adi_10 Adi-10
Adi_12 Adi-12
Adi_2 Adi-2
Bd18_1 Bd18-1
Bd2_3 Bd2-3
Bd21_3 Bd21-3
Bd29_1 Bd29-1
Bd3_1 Bd3-1
Bis_1 Bis-1
Gaz_8 Gaz-8
Kah_1 Kah-1
Kah_5 Kah-5
Koz_1 Koz-1
Koz_3 Koz-3
Tek_2 Tek-2
BdTR6g-VSM BdTR6g
TE4_VSM TE4.3

***Reply: Thank you for your suggestion. We have revised the incorrect usage of species and genome assembly names throughout the manuscript and supplementary table.***

They have not cited the source of the germplasm used. I notice that the B. hybridum and B. stacei lines came from Israel, but I did not see a reference or a collaborator who collected the seeds. They should include the source of all germplasm used in the materials and methods and provide a citation or indicate that they have permission of the collector. This is standard practice in the scientific community. This should also be included in Table s19.

***Reply: We apologize for the oversight regarding the omission of the germplasm source citation in the manuscript. To rectify this, we have included detailed information about the sources of all germplasm used in the materials and methods section. Additionally, we have provided proper citations for this Table to ensure the accuracy and credibility of our work.***

They should also cite the sources of the sequences used and not just indicate the source as NCBI or some other database. For example, I believe they used sequences from the Brachypodium pangenome paper by Gordon et al., and the Brachypodium genome paper.
***Reply: Thank you for your suggestion. We have added two citations for the sources of the* *sequences used throughout the manuscript.***

***Reference:***

*(1). Gordon SP, Contreras-Moreira B, Levy JJ, Djamei A, Czedik-Eysenberg A, Tartaglio VS, Session A, Martin J, Cartwright A, Katz A, et al: Gradual polyploid genome evolution revealed by pan-genomic analysis of Brachypodium hybridum and its diploid progenitors. Nat Commun 2020, 11:3670.*

*(2). Gordon SP, Contreras-Moreira B, Woods DP, Des Marais DL, Burgess D, Shu S, Stritt C, Roulin AC, Schackwitz W, Tyler L, et al: Extensive gene content variation in the Brachypodium distachyon pan-genome correlates with population structure. Nat Commun 2017, 8:2184.*

They included whole genome analysis of two unpublished genomes, B. sylvaticum and B. mexicanum. These genomes are still under restricted use as described on the genome pages in Phytozome. They would have had to see these restrictions when they downloaded the sequences from Phytozome. If they want to include these analyses, they should contact the project leaders listed on the Phytozome genome pages for these organisms:
<https://phytozome-next.jgi.doe.gov/info/Bmexicanum_v2_1>
<https://phytozome-next.jgi.doe.gov/info/BsylvaticumAin_1_v2_1>
***Reply: Thank you for your suggestion. We used Bmexicanum_v1_1 genome from Phytozome (***[***https://phytozome-next.jgi.doe.gov/info/Bmexicanum_v1_1***](https://phytozome-next.jgi.doe.gov/info/Bmexicanum_v1_1)***), and now have updated to v2_1 as you provided. The genome is public with unrestricted but citation required. We added the citation [1] as described on the genome pages in our revised manuscript. The B. sylvaticum genome has use restrictions, and we have removed this genome data. It doesn’t impact our conclusion.***

***Reference:***

*(1). Gordon, S. P., Contreras-Moreira, B., Levy, J. J., Djamei, A., Czedik-Eysenberg, A., Tartaglio, V. S., … Vogel, J. P. (2020). Gradual polyploid genome evolution revealed by pan-genomic analysis of Brachypodium hybridum and its diploid progenitors. Nature Communications, 11(1).* [*https://doi.org/10.1038/s41467-020-17302-5*](https://doi.org/10.1038/s41467-020-17302-5)*.*

They did not do a de-novo annotation of non-TE genes. Rather, they used a lift over from the existing reference genomes. This will miss genes for the B. hybridum and B. stacei genomes due to intraspecific variation. While this is irrelevant for their centromere analysis, they should note this limitation.
***Reply: Thank you for your suggestion. We have added the statement of limitations in the result.***

They have an excessive number of supplementary tables and figures. I encourage them to reduce the number to those needed to convey the main findings. For example, they really don't need k-mer graphs to estimate genome sizes for these well-studied species.

***Reply: Thank you for your suggestions. We have removed several figures and tables (Figure S1, Table S1 and Table S10).***

**Specific comments**
They should mention if IBd483 is a newer or older type of B. hybridum.

***Reply: The IBd483 lines used in our study are older type of B. hybridum. We have added it in the methods.***

Figure S4 a&b it looks like the numbering of some chromosomes are inverted because the orientation of the line on the dot plots are going in different directions. They should fix this.
***Reply: Thank you for your suggestions. We have fixed in the manuscript and modified some figures.***

Figure S14 is very hard to understand. ***Reply: We are sorry for the hard understanding of the figure, and we have added more information in the figure legends.***

Fig S22 remove the black lines or make them under the colored lines so you can see the trends easier. Currently, you have to zoom way in to see the colors.
***Reply: Thank you for your suggestions. We have revised the figure.***

Page 2 line 26 Fix the sentence containing "resulting the frequency"
***Reply: Thank you for your suggestions. We have revised the sentence as “and the frequency of retrotransposon invasions drove the degree of inter-species centromere diversifications”.***

Page 3 line 27 delete "arising from wide hybridization" because polyploids can also arise from genome duplication without a wide cross, autopolyploidy.
***Reply: Thank you for your suggestions. We have revised the sentence as “Allopolyploidy, arising from wide hybridization and whole-genome duplication (WGD)”.***

Page 8 line 25 change descripted to described.
***Reply: Thank you for your suggestions. We have revised the word as “described”.***

Page 9 line 19 awkward sentence
***Reply: We have changed the sentence to make the intended meaning more clearly. “We found that there were no pair-wise satellite repeats sharing between the D and S sub/genomes in our three assemblies.”***

Page 9 line 35 add "the" between of and satellite.
***Reply: We have added “the” between of and satellite.***

Page 22 change comprehensive to comprehensively.
***Reply: We have changed it.***

Page 22 line 54 define "natural conditions".
***Reply: Thank you for your suggestions. We have changed the sentence as “The plants were then transferred to the tissue culture room under optimal conditions (16 h day/8h night: 24ºC/18 ºC; 200 µmol/m^2^/s light).”***

Page 23 line 31 define "certain amount".
***Reply: Thank you for your suggestions. We have changed as “3-5 g young fresh leaves tissue”.***

Page24 line 19 add reference for Bd21 genome.
***Reply: Thank you for your suggestions. We have add the reference for Bd21 genome.***

Page 28 line 19 delete "Metadata of".
***Reply: Thank you for your suggestions. We have deleted.***

Figure 1a the innermost synteny plot is useless. Either delete or add individual colors for different chromosomes. Also, it appears they only indicate synteny between certain genomes/subgenomes. They should mention this.
***Reply: We have deleted the innermost synteny plot of figure 1.***

Figure 3e I am not sure what the Q scale means.
***Reply: The “Q scale” refers to the quantile grouping of the satellite sequences. The grouping is based on either their CENH3 occupancy, represented by the log2(ChIP/input) value, or their sequence identity as calculated through comparison with the consensus satellite repeat. We categorized the satellite sequences into five distinct groups. These groups were organized in descending order of CENH3 log2(ChIP/input) values (light red, cyan, blue, green, yellow). The same groups of satellite sequences were subjected to analysis based on their sequence identity.***

Figure 4 g-h it is unclear top me what this is.
***Reply: We are sorry for the unclear. Figure 4g-h shows the metaprofiles of CENH3 ChIP-seq signals around CentBd/CentBs satellite repeats, centromeric intact CRM elements in the three genomes. The peaks reflect a phased distribution of CENH3 nucleosomes across the centromeric repeat sequences.*** ***We also added detailed explanations in the figure legends to enhance the readers' comprehension of the figures and the associated findings.***

Figure 5 b I'm not sure what the y axes designate.
***Reply: Thank you for your suggestions. In Figure 5b, we present the PCA (Principal component analysis) projection on PC1 (first principal component) and PC2 (second principal component) of sequencing fragments encompassing CentBd or CentBs satellite repeats. These repeats are derived from the pan-genomes of distinct lines within the B. hybridum, B. distachyon and B. stacei species. Box plots positioned at the top and right corners of the PCA projection visually represent the overall distribution of PC1 and PC2 scores across sub/genome groups. The y axes denotes the PCA2 value, and we have moved the PC2 (8.6%) from the top to the left side of the y axes, enhancing the clarity in the presentation. We also added these information in the figure legends.***

Table 1 the listed BUSCO scores may be of a little since they did a lift over annotation. However, if anything they would be improved with a proper annotation.

***Reply: Thank you for your suggestions. We have compared the BUSCO scores across various recent genome assemblies of model plants. Notably, our three novel assemblies* *exhibited BUSCO scores ranging from 98.6%–99.5%. These scores stand in direct comparison with values observed in other genome assemblies (ranging from 97.8% to 99.88%), encompassing assemblies from 1 Arabidopsis, 3 rice, and 1 E. rufipilus [1].***

***Our primary emphasis was on conducting a comprehensive comparative analysis of previously uncharted centromere regions across species with varying polyploidy levels. Consequently, we did not encompass gene investigations. We conducted a lift-over gene annotation strategy, wherein we mapped models from previously well annotated reference genomes [2, 3]. We have also incorporated this limitations in the manuscript.***

***Reference:***

*1. Wang T, Wang B, Hua X, Tang H, Zhang Z, Gao R, Qi Y, Zhang Q, Wang G, Yu Z, et al: A complete gap-free diploid genome in Saccharum complex and the genomic footprints of evolution in the highly polyploid Saccharum genus. Nat Plants 2023, 9:554-571.*

*2. Gordon SP, Contreras-Moreira B, Levy JJ, Djamei A, Czedik-Eysenberg A, Tartaglio VS, Session A, Martin J, Cartwright A, Katz A, et al: Gradual polyploid genome evolution revealed by pan-genomic analysis of Brachypodium hybridum and its diploid progenitors. Nat Commun 2020, 11:3670.*

*3. Gordon SP, Contreras-Moreira B, Woods DP, Des Marais DL, Burgess D, Shu S, Stritt C, Roulin AC, Schackwitz W, Tyler L, et al: Extensive gene content variation in the Brachypodium distachyon pan-genome correlates with population structure. Nat Commun 2017, 8:2184.*

**Second round of review**

**Reviewer 1**

The authors have made efforts to improve the manuscript. Regarding the responses to comments 2 and 3, further illustrations of results are necessary to provide a clear view of the syntenic relationships.

1. Comment to the response to comment 2

The authors observed the absence of centromere sequences and structural conservations between these shared unique pairs of chromosomes. They could add the detailed syntenic relationship maps of O. sativa, B. stacei, B. distachyon, and B. hybridium. For instance, the authors could modify the previously reported syntenic relationship figure (please see Figure 2a in Gordon et al., 2020) by highlighting which centromeres were lost, divergent, and conserved. It is also important to add the tetraploid B. hybridium into the whole relationship figure. It will be clear to illustrate the different evolutionary paths between the centromeres and other chromosome regions.

2. Comment to the response to comment 3

The Fig. S12 is not color-friendly. The alignment of chromosomes into linear lines makes it very difficult to track the syntenic relationships. The authors should illustrate the syntenic relationships for satellite arrays and CRM elements with clear information on rearrangements, turnovers, divergences, and conservations.

3. Comments to the responses to the minor comment 2.

The figure legends still lack quite a lot of information. For instance, in Fig. S10, it is not clear what data were dot-plotted. It is not clear why a search window of 156 bp was used. It is also unclear what statistical methods were used to determine the similarity. The fonts in many figures were too small, such as Fig. S13. I suggest the authors could generate figures with high quality along with clear and detailed information.

**Reviewer 2**

My concerns have been addressed by the authors and I have no more comments on the manuscript.

**Reviewer 3**

The authors must remove the B. mexicanum genome analysis or contact the PI of the project to arrange a collaboration. This is an unpublished genome. The authors correctly note that the Phytozome genome page lists the genome as freely available and lists a citation. However, this is an obvious error and I have asked the curators to fix it and it will be fixed soon. Just reading the title of the citation reveals that the paper does not describe B. mexicanum. Did the authors even read the cited paper? Did it not raise any red flags when B. mexicanum was not mentioned once in the paper? Not even after a reviewer tells them it has not been published? The authors do not seem to recognize the fact generators of genomes put time and effort into generating them and would like the opportunity to publish first or be consulted as to the appropriateness of any whole genome analyses. Genomes are posted as early release to benefit the scientific community by facilitating researchers studying a few genes or to spur collaborations using whole genome analyses. I have been involved in many of these projects and have been a strong advocate of early release. I am appalled by this blatant attempt to exploit and obvious error. When a reviewer points out that a genome has not been published, the authors should take this allegation of scientific misconduct seriously. It appears that these authors are trying to exploit an error rather than do the right thing.

**Authors Response**

**Point-by-point responses to the reviewers’ comments:**

Reviewer #1:

The authors have made efforts to improve the manuscript. Regarding the responses to comments 2 and 3, further illustrations of results are necessary to provide a clear view of the syntenic relationships.

1. Comment to the response to comment 2

The authors observed the absence of centromere sequences and structural conservations between these shared unique pairs of chromosomes. They could add the detailed syntenic relationship maps of O. sativa, B. stacei, B. distachyon, and B. hybridium. For instance, the authors could modify the previously reported syntenic relationship figure (please see Figure 2a in Gordon et al., 2020) by highlighting which centromeres were lost, divergent, and conserved. It is also important to add the tetraploid B. hybridium into the whole relationship figure. It will be clear to illustrate the different evolutionary paths between the centromeres and other chromosome regions.

***Reply: Thank you for your suggestion. We have modified the previously reported syntenic chromosome relationship maps of O. sativa, B. stacei, B. distachyon, and B. hybridum (Gordon et al., 2020) by highlighting the centromere regions (Fig S10). The syntenic chromosome relationships reveal that Brachypodium chromosome evolved from an ancestral chromosome through a series of nested insertions of whole chromosomes, fission* *of two* *chromosomes, or collinearity of chromosome. These results clearly demonstrate centromere divergences between rice and B. stacei or between B. stacei and B. distachyon, suggesting post-speciation turnover of centromeres. Furthermore, the centromere loss was illustrated during the nested insertions of chromosomes or fusion of chromosomes between O. sativa and Brachypodium genus. Conserved centromeres were shown between Bd21 or Bst99 chromosomes with those chromosomes of D- and S- subgenomes of B. hybridum, respectively. Other chromosome regions exhibit a predominantly collinear pattern in the syntenic relationship maps, indicating different evolutionary paths between the centromeres and chromosome-arm regions.***

***Fig. S10 Syntenic chromosome relationships and centromere evolution among O. sativa, B. distachyon, B. stacei, and B. hybridum genome.***

2. Comment to the response to comment 3

The Fig. S12 is not color-friendly. The alignment of chromosomes into linear lines makes it very difficult to track the syntenic relationships. The authors should illustrate the syntenic relationships for satellite arrays and CRM elements with clear information on rearrangements, turnovers, divergences, and conservations.

***Reply: Thank you for your suggestion. We have regenerated the alignment figure and split it according to the syntenic chromosomes relationships among O. sativa, B. stacei, and B. distachyon. Comparative analysis of centromere sequences (satellite arrays and CRM elements) of syntenic chromosomes indicated significant divergence in centromere sequences across these species. This finding underscores the occurrence of rapid interspecies centromere evolution and complete turnover during or after speciation.***

3. Comments to the responses to the minor comment 2.

The figure legends still lack quite a lot of information. For instance, in Fig. S10, it is not clear what data were dot-plotted. It is not clear why a search window of 156 bp was used. It is also unclear what statistical methods were used to determine the similarity. The fonts in many figures were too small, such as Fig. S13. I suggest the authors could generate figures with high quality along with clear and detailed information.

***Reply: We have recreated these figures with improved quality and included detailed information in the legends. We have also added the region information for the dot-plotted centromere of each chromosome in the figure legends of the revised manuscript. The choice of 156-bp search window was based on the approach used in previous Arabidopsis centromere dot plots, which utilized a unit-size of centromere satellite repeat (Naish et al., 2021). We used the redotable program, which generates a dot plot based on word-based alignment with a specified length and a perfect match between the two sequences (***[***https://www.bioinformatics.babraham.ac.uk/projects/redotable/).***](https://www.bioinformatics.babraham.ac.uk/projects/redotable/).%20) ***When the residues of both sequences are identical, a dot is drawn at the corresponding position in the plot. Furthermore. We have regenerated Figures S8, S12, S13, S14 and S15, and have enlarged the fonts while providing additional information in the legends.***

***Reference:***

*Naish M, Alonge M, Wlodzimierz P, Tock AJ, Abramson BW, Schmucker A, Mandakova T, Jamge B, Lambing C, Kuo P, et al: The genetic and epigenetic landscape of the Arabidopsis centromeres. Science 2021, 374:eabi7489.*

Reviewer #2:

My concerns have been addressed by the authors and I have no more comments on the manuscript.

***Reply: Thank you for your valuable comments on our manuscript.***

Reviewer #3: The authors must remove the B. mexicanum genome analysis or contact the PI of the project to arrange a collaboration. This is an unpublished genome. The authors correctly note that the Phytozome genome page lists the genome as freely available and lists a citation. However, this is an obvious error and I have asked the curators to fix it and it will be fixed soon. Just reading the title of the citation reveals that the paper does not describe B. mexicanum. Did the authors even read the cited paper? Did it not raise any red flags when B. mexicanum was not mentioned once in the paper? Not even after a reviewer tells them it has not been published? The authors do not seem to recognize the fact generators of genomes put time and effort into generating them and would like the opportunity to publish first or be consulted as to the appropriateness of any whole genome analyses. Genomes are posted as early release to benefit the scientific community by facilitating researchers studying a few genes or to spur collaborations using whole genome analyses. I have been involved in many of these projects and have been a strong advocate of early release. I am appalled by this blatant attempt to exploit and obvious error. When a reviewer points out that a genome has not been published, the authors should take this allegation of scientific misconduct seriously. It appears that these authors are trying to exploit an error rather than do the right thing.

***Reply: Thank you for your suggestions. We feel it is necessary to make a declaration. Firstly, we absolutely respect the tremendous efforts of the reviewer to assemble the genome B. mexicanum. Secondly, we genuinely had no knowledge that this was an artificial error. The Phytozome genome list still indicated that B. mexicanum genome was publicly available when we returned our revised manuscript. We are more than willing to rectify this misunderstanding and are open to discussing the potential collaboration. On the other hand, after thoroughly discussions with all co-authors and a comprehensive review of our results, we all believe that it would have no impact on our results if we remove the related results of B. mexicanum.***

**Third round of review**

**Reviewer 1**

The authors have fully addressed my comments.

**Reviewer 3**

The authors have addressed all of my concerns.
